# Classification and nomenclature of all human homeobox genes

**DOI:** 10.1186/1741-7007-5-47

**Published:** 2007-10-26

**Authors:** Peter WH Holland, H Anne F Booth, Elspeth A Bruford

**Affiliations:** 1 Department of Zoology, University of Oxford, South Parks Road, Oxford, OX1 3PS, UK; 2 HUGO Gene Nomenclature Committee, European Bioinformatics Institute (EMBL-EBI), Wellcome Trust Genome Campus, Hinxton, Cambridgeshire, CB10 1SA, UK

## Abstract

**Background:**

The homeobox genes are a large and diverse group of genes, many of which play important roles in the embryonic development of animals. Increasingly, homeobox genes are being compared between genomes in an attempt to understand the evolution of animal development. Despite their importance, the full diversity of human homeobox genes has not previously been described.

**Results:**

We have identified all homeobox genes and pseudogenes in the euchromatic regions of the human genome, finding many unannotated, incorrectly annotated, unnamed, misnamed or misclassified genes and pseudogenes. We describe 300 human homeobox loci, which we divide into 235 probable functional genes and 65 probable pseudogenes. These totals include 3 genes with partial homeoboxes and 13 pseudogenes that lack homeoboxes but are clearly derived from homeobox genes. These figures exclude the repetitive *DUX1 *to *DUX5 *homeobox sequences of which we identified 35 probable pseudogenes, with many more expected in heterochromatic regions. Nomenclature is established for approximately 40 formerly unnamed loci, reflecting their evolutionary relationships to other loci in human and other species, and nomenclature revisions are proposed for around 30 other loci. We use a classification that recognizes 11 homeobox gene 'classes' subdivided into 102 homeobox gene 'families'.

**Conclusion:**

We have conducted a comprehensive survey of homeobox genes and pseudogenes in the human genome, described many new loci, and revised the classification and nomenclature of homeobox genes. The classification scheme may be widely applicable to homeobox genes in other animal genomes and will facilitate comparative genomics of this important gene superclass.

## Background

Homeobox genes are characterized by the possession of a particular DNA sequence, the homeobox, which encodes a recognizable although very variable protein domain, the homeodomain [1,2]. Most homeodomains are 60 amino acids in length, although exceptions are known. Many homeodomain proteins are transcription factors with important roles in embryonic patterning and cell differentiation, and several have been implicated in human diseases and congenital abnormalities [3].

The homeobox genes have been variously subdivided into superclasses, classes, subclasses or groups, although there has been much inconsistency in the use of these terms. The most commonly recognized groupings are the ANTP, PRD, LIM, POU, HNF, SINE, TALE, CUT, PROS and ZF groups (or variants of these names), although these are not always given equal rank in classification schemes [1,2,4-8]. There is more consensus in classification at a lower level, just above the level of the gene, where very similar genes are grouped into gene families. Widely recognized gene families include Dlx, Evx, Msx, Cdx, En, Otx, Pitx, Otx and Emx (or variants of these names), amongst many others, although there is variation particularly concerning how many gene families are used for the HOX, PAX and NK homeobox genes. Despite the numerous discrepancies, the common principle of classification is the same. The goal of any scheme is to mirror evolutionary diversification, so that 'closely related' genes are placed in the same gene family, and related gene families are placed in the same gene class or other higher grouping. It should be borne in mind, however, that the pathway of evolutionary diversification is never completely known for any large and complex set of genes.

The initial analyses of the draft human genome sequence published in 2001 included estimates of the number of human homeobox genes. Venter et al [9] found 160 homeobox genes, containing 178 homeobox sequences, using large-scale automated classification; while the IHGSC team [10] gave a much higher estimate of 267 homeobox genes. Both were based on draft coverage of the human genome and would be expected to be missing some genes, as well as confusing pseudogenes with genes. In the same year, Banerjee-Basu and Baxevanis [8] presented an analysis of 129 human homeodomain sequences, but this was far from a comprehensive survey. More recently, there have been two more accurate surveys of homeobox genes in the human genome. Nam and Nei [11] found 230 homeobox genes, containing 257 homeobox sequences. Ryan et al [7] found 228 homeodomain sequences in the NCBI RefSeq database of October 2004. Our analyses (described here) revealed many homeobox genes that were incorrectly annotated, named or classified and many homeobox pseudogenes that had previously been missed. We report a complete survey of homeobox loci in the euchromatic regions of the human genome, appropriate gene nomenclature and a consistent classification scheme.

## Results and Discussion

### How many homeobox genes and pseudogenes?

Using exhaustive database screening, followed by manual examination of sequences, we identified 300 homeobox loci in the human genome. Distinguishing which of these loci are functional genes and which are non-functional pseudogenes was difficult in some cases. Most loci classified as pseudogenes in this study are integrated reverse-transcribed transcripts, readily recognized by their dispersed genomic location, complete lack of intron sequences, and (in some cases) 3' homopolymeric run of adenine residues. A small minority are duplicated copies of genes, recognized by physical linkage to their functional counterparts and the same (or similar) exon-intron arrangement. In general, retrotransposed gene copies are non-functional (and therefore pseudogenes) from the moment of integration because they lack 5' promoter regions necessary for transcription. However, such sequences can occasionally acquire new promoters and become functional as 'retrogenes'. Duplicated gene copies often possess 5'promoter regions (as they are often encompassed by the duplication event); most degenerate to pseudogenes due to redundancy in a process known as non-functionalization, however some can be preserved as functional genes through sub- or neo-functionalization. Thus, in both instances, reliable indicators of non-functionality were sought in order to assign pseudogene status, notably frameshift mutations, premature stop codons and non-synonymous substitutions at otherwise conserved sites in the original coding region.

We currently estimate that the 300 human homeobox loci comprise 235 functional genes and 65 pseudogenes (Table 1). These figures include three functional genes that possess partial homeobox sequences (*PAX2*, *PAX5 *and *PAX8*) and retrotransposed pseudogenes that correspond to only part of the original transcript, whether or not it includes the homeobox region or indeed any of the original coding region. Consequently, 13 retrotransposed pseudogenes that lack homeobox sequences are included (*NANOGP11*, *TPRX1P1*, *TPRX1P2*, *POU5F1P7*, *POU5F1P8*, *IRX4P1*, *TGIF2P2*, *TGIF2P3*, *TGIF2P4*, *CUX2P1*, *CUX2P2*, *SATB1P1*, *ZEB2P1*). We do not include *PAX1*, *PAX9 *and *CERS1*; these are functional genes without homeobox motifs, albeit closely related to true homeobox genes (the other PAX and CERS genes).

**Table 1:** Numbers of human genes, pseudogenes and gene families in each homeobox gene class. The human homeobox gene superclass contains a total of 235 probable functional genes and 65 probable pseudogenes. These are divided between 102 gene families, which are in turn divided between eleven gene classes.

**Class**	**Subclass**	**Number of gene families**	**Number of genes**	**Number of pseudogenes**
ANTP	HOXL	14	52	0
	NKL	23	48	19^b^
PRD	PAX	3	7^a^	0
	PAXL	28	43	24^c, d^
LIM		6	12	0
POU		7	16	8^e^
HNF		2	3	0
SINE		3	6	0
TALE		6	20	10^f^
CUT		3	7	3^g^
PROS		1	2	0
ZF		5	14	1^h^
CERS		1	5^i^	0

***Totals***		***102***	***235*^*a*^**	***65*^*b-h*^**

^a^Includes *PAX2*, *PAX5 *and *PAX8 *that have a partial homeobox; excludes *PAX1 *and *PAX9 *that lack a homeobox.

^b^Includes *NANOGP11 *that lacks a homeobox.

^c^Excludes intronless and repetitive *DUX1 *to *DUX5 *sequences.

^d^Includes *TPRX1P1 *and *TPRX1P2 *that lack a homeobox.

^e^Includes *POU5F1P7 *and *POU5F1P8 *that lack a homeobox.

^f^Includes *IRX4P1*, *TGIF2P2*, *TGIF2P3 *and *TGIF2P4 *that lack a homeobox.

^g^Includes *CUX2P1*, *CUX2P2 *and *SATB1P1 *that lack a homeobox.

^h^Includes *ZEB2P1 *that lacks a homeobox.

^i^Excludes *CERS1 *that lacks a homeobox.

The total number of homeobox sequences in the human genome is higher than 300 for two reasons. First, several genes and pseudogenes possess more than one homeobox sequence, notably members of the Dux (double homeobox), Zfhx and Zhx/Homez gene families. Second, we have excluded a set of sequences related to human *DUX4 *(*DUX1 *to *DUX5*), which have become part of 3.3 kb repetitive DNA elements present in multiple copies in the genome [12-14]. Few of these tandemly-repeated sequences are likely to be functional as expressed proteins, and all were probably derived by retrotransposition from functional DUX gene transcripts (see below). The fact that they are not included in the total count, therefore, is likely to have limited bearing on understanding the diversity and normal function of human homeobox genes. Hence, our figure of 300 homeobox loci is the most useful current estimate of the repertoire of human homeobox genes and pseudogenes.

### Classification

We propose a simple classification scheme for homeobox genes, based on two principal ranks: gene class and gene family. A gene class contains one or more gene families, which in turn will contain one or more genes. In a few cases, it is useful to erect an intermediate rank between these levels, and for this we use the term subclass. For the entire set of homeobox genes, we use the term superclass.

For the rank of gene family, we use a specific evolutionary-based definition based on common practice in the field of comparative genomics and developmental biology. We define a gene family as a set of genes derived from a single gene in the most recent common ancestor of bilaterian animals (here defined as the latest common ancestor of *Drosophila *and human). This definition has been made explicitly in previous work [2,6] but is actually a principle that has been in widespread, but rather inconsistent, use for over a decade [15]. For example, amongst the homeobox genes, the En (engrailed) gene family was originally defined to include human *EN1 *and *EN2*, plus *Drosophila en *and *inv *[16]; these four genes arose by independent duplication from a single gene in the most recent common ancestor of insects and vertebrates. Moving outside the homeobox genes, this principle is also widespread; for example, the Hh (hedgehog) gene family was defined to include mouse *Shh*, *Dhh *and *Ihh*, plus *Drosophila hh *[17]. To clarify boundaries between gene families, we conducted molecular phylogenetic analyses of human homeodomain sequences, using a range of protostome and occasionally cnidarian homeodomain sequences as outgroups (Additional files 1 and 2).

While the gene family definition described above is generally workable for homeobox genes, by necessity there are some exceptions. One type of exception relates to genes with an unknown ancestral number. For example, there is uncertainty as to whether there were one or two Dlx (distal-less) genes in the most recent common ancestor of bilaterians; however it is common practice to refer to a single Dlx gene family [18]. Thus, we stick with convention for this set of genes. There is similar uncertainty over the ancestral number of Irx (iroquois) genes [19], and again we treat these as a single gene family. The HOX genes are an interesting case as their precise number in the most recent common ancestor of bilaterians is unknown due to lack of phylogenetic resolution between 'central' genes [20]. Here we divide the HOX genes into seven gene families: the 'anterior' Hox1 and Hox2 gene families, the 'group 3' Hox3 gene family, the 'central' Hox4, Hox5 and Hox6-8 gene families, and the 'posterior' Hox9-13 gene family. Another type of exception relates to 'orphan' genes. These are genes that have been found in one species (for example human) but not in other species, or at least not in a wide diversity of Metazoa. Some of these will be ancient genes that have been secondarily lost from the genomes of some species, in which case these comply with our evolutionary definition of a gene family made above. Others, however, will be rapidly evolving genes that originated from another homeobox gene and then diverged to such an extent that their origins are unclear [21]. Whenever origins are unclear, we must define a new gene family to encompass those genes, even though they may not date back to the latest common ancestor of bilaterians. In these cases, the gene family is erected to recognize a set of distinct genes on the basis of DNA and protein sequence, rather than on evolutionary origins.

Using the aforementioned criteria, we recognize 102 homeobox gene families in the human genome (Table 1). We are aware that other homeobox gene families exist in bilaterians but have been lost from humans (for example, Nk7, Ro, Hbn, Repo and Cmp; [7]), and we recognize that some gene family boundaries will alter as new information is obtained. Nonetheless, at the present time the 102 gene families provide a sound framework for the study of human homeobox genes.

**Figure 1:**
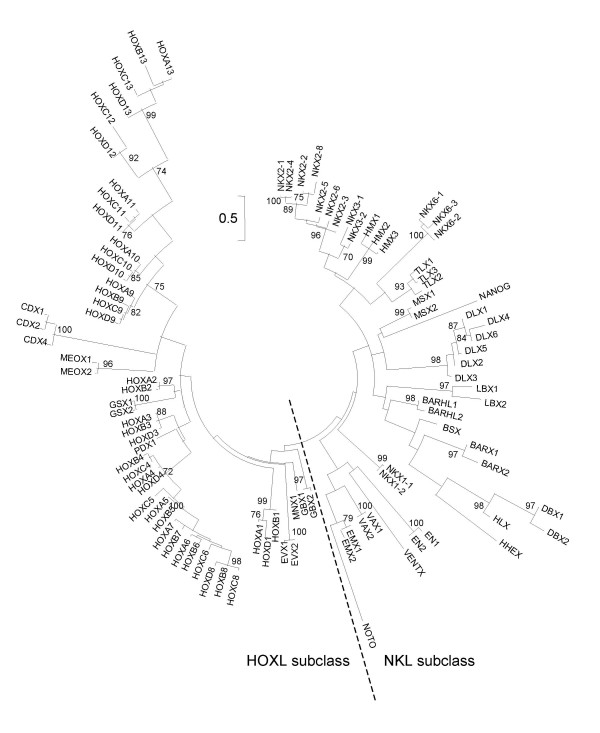
**Maximum likelihood phylogenetic tree of human ANTP-class homeodomains**. Arbitrarily rooted phylogenetic tree of human ANTP-class homeodomains constructed using the maximum likelihood method. Bootstrap values supporting internal nodes with over 70% are shown. Homeodomain sequences derived from pseudogenes are excluded. The proposed division between the HOXL and NKL subclasses is indicated. The position of *EN1 *and *EN2 *is unstable; this tree places them in the NKL subclass, whereas neighbor-joining analysis of the same dataset places them at the base of the two subclasses (Additional file 3). Interrelationships of genes in the Nk4 and Nk2.2 families are also unstable (in this tree and Additional file 3 respectively); in these cases synteny within and between genomes clearly resolves gene families. Detailed relationships between different gene families should not be inferred from this tree.

**Figure 2:**
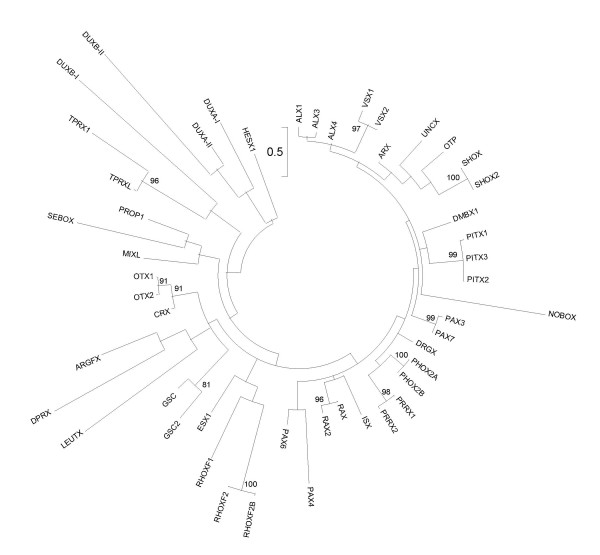
**Maximum likelihood phylogenetic tree of human PRD-class homeodomains**. Arbitrarily rooted phylogenetic tree of human PRD-class homeodomains constructed using the maximum likelihood method. Bootstrap values supporting internal nodes with over 70% are shown. Homeodomain sequences derived from pseudogenes are excluded, as are the partial homeodomains of PAX2, PAX5 and PAX8, and the HOPX homeodomain because its extremely divergent sequence destabilizes the overall tree topology. Roman numeral suffixes are used to distinguish multiple homeodomains encoded by a single Dux-family gene. In this tree Dux-family homeodomains are not monophyletic, even within the same gene; however, monophyly is recovered by neighbor-joining analysis (Additional file 4). Detailed relationships between different gene families should not be inferred from this tree.

**Figure 3:**
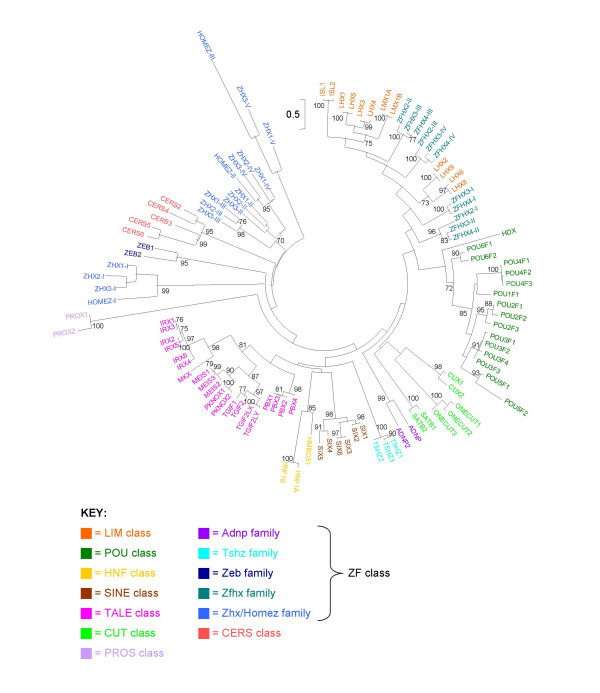
**Maximum likelihood phylogenetic tree of human homeodomains excluding ANTP and PRD classes**. Arbitrarily rooted phylogenetic tree of human homeodomains excluding the ANTP and PRD classes constructed using the maximum likelihood method. Bootstrap values supporting internal nodes with over 70% are shown. Homeodomain sequences derived from pseudogenes are excluded. Roman numeral suffixes are used to distinguish multiple homeodomains encoded by a single gene. Classes and/or families are color coded as shown in the key. The LIM and ZF classes are not recovered as two distinct monophyletic groups, a result also found by neighbor-joining analysis (Additional file 5). The multiple homeodomains of Zfhx-family proteins and Zhx/Homez-family proteins are also dispersed in the tree, presumably artefactually. Detailed relationships between different gene families should not be inferred from this tree.

It is much more difficult to propose a rigorous evolutionary definition for the rank of gene class. Every attempt to classify genes above the level of gene family involves a degree of arbitrariness. We define gene classes by taking two principal criteria into account. First, gene classes should ideally be monophyletic assemblages of gene families. To identify probable monophyletic groups of gene families, we conducted molecular phylogenetic analyses of homeodomain sequences, and looked for sets of gene families that group together stably, regardless of the precise composition of the dataset used (Figures 1, 2, 3; Additional files 3, 4, 5). Some gene families were difficult to place from sequence data alone, and were found in different gene classes (or subclasses) depending on the precise dataset analyzed or the phylogenetic method employed. This is perhaps not surprising as trees that encompass many homeobox genes can only be built with a short sequence alignment (the homeodomain); under these conditions, phylogenetic trees can only be used as a guide to possible classification, not the absolute truth. In ambiguous cases, we used the chromosomal location of genes to guide possible resolution between alternative hypotheses. Second, some homeobox gene classes can be characterized by the presence of additional protein domains outside of the homeodomain [2]. Recognized protein domains associated with homeodomains include the PRD domain, LIM domain, POU-specific domain, POU-like domain, SIX domain, various MEINOX-related domains, the CUT domain, PROS domain, and various ZF domains [2].

Using the aforementioned criteria, we recognize eleven homeobox gene classes in the human genome: ANTP, PRD, LIM, POU, HNF, SINE, TALE, CUT, PROS, ZF and CERS (Table 1). There is no expectation that the eleven gene classes will be of similar size, simply because some classes will have undergone more expansion by gene duplication than others. In the human genome, the ANTP and PRD classes are much larger than the other classes. Although gene classes should ideally be monophyletic, it is possible that the ZF homeobox gene class, characterized by the presence of zinc finger motifs in most of its members, is polyphyletic (Figure 3; Additional file 5). In other words, domain shuffling may have brought together a homeobox sequence and a zinc finger sequence on more than one occasion. The same may also be true for the LIM class; alternatively the apparent polyphyly of LIM-class homeodomains could be a consequence of LIM domain loss or artefactual placement of some ZF-class homeodomains in phylogenetic analyses (Figure 3; Additional file 5).

In theory, it is possible to recognize higher level associations above the level of the gene class, because the diversification of homeobox genes will have taken place by a continual series of gene duplication events. We do not propose names for hierarchical levels above the rank of class, and consider that gene name, gene family and gene class (and occasionally subclass) convey sufficient information for most purposes.

We use a consistent convention for writing gene classes and gene families. We present the names of all gene classes in abbreviated non-italicized upper case – for example, the ANTP and PRD classes – to avoid confusion with gene symbols (*Antp *and *prd*) or indeed gene names (*Antennapedia *and *paired*). In contrast, we present the names of all gene families in non-italicized title case; for example, the Cdx, En and Gsc gene families. We have used this style consistently in recent work [6,21-23] and note that several other authors have done likewise [4,7,24]. We suggest that this style, and most of these gene family names, can be used in other bilaterian genomes. Extending the scheme to non-bilaterians is more difficult, however, and awaits clarification of the relationship between the homeobox genes of sponges, placozoans, cnidarians and bilaterians [7,25].

### The ANTP homeobox class

**Figure 4:**
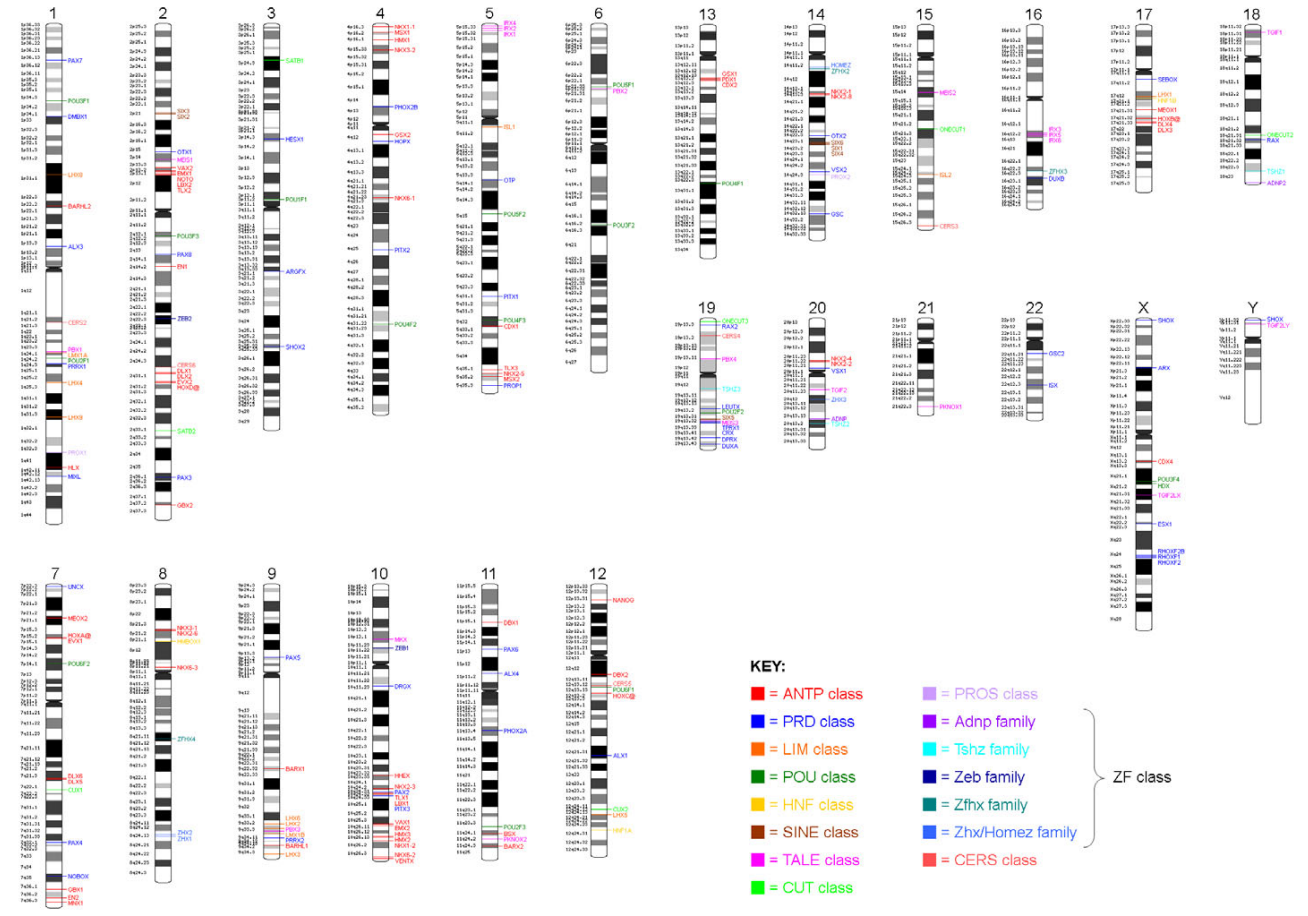
**Chromosomal distribution of human homeobox genes**. Ideograms of human chromosomes showing the locations of human homeobox genes. Hox clusters are each shown as a single line for simplicity. Probable pseudogenes are not shown. Genes are color coded according to their class or family (see key). Map positions were obtained through the Ensembl Genome Browser.

The ANTP class derives its name from the *Antennapedia *(*Antp*) gene, one of the HOX genes within the ANT-C homeotic complex of *Drosophila melanogaster*. The human genome has 39 HOX genes, arranged into four Hox clusters. Here we divide the HOX genes into seven gene families: Hox1, Hox2, Hox3, Hox4, Hox5, Hox6-8 and Hox9-13. The HOX genes are not the only ANTP-class genes, and we recognize a total of 37 gene families in this class (Table 1). We divide these 37 gene families between two subclasses that are relatively well-supported in phylogenetic analyses: the HOXL and the NKL subclasses (Figure 1; Additional file 3). As previously discussed, the subclasses are largely consistent with the chromosomal positions of genes [26,27]. The HOXL (HOX-Like or HOX-Linked) genes primarily map to two fourfold paralogous regions: the Hox paralogon (2q, 7p/q, 12q and 17q) and the ParaHox paralogon (4q, 5q, 13q and Xq) (Figure 4). The NKL (NK-Like or NK-Linked) genes are more dispersed, but there is a concentration on the NKL or MetaHox paralogon (2p/8p, 4p, 5q and 10q) (Figure 4). Somewhat aberrantly, the Dlx and En gene families group with the NKL subclass in phylogenetic analyses (Figure 1; Additional file 3), but with the HOXL subclass on the basis of chromosomal positions (Figure 4).

Most of the 37 gene families in the ANTP class have been clearly defined before. We draw attention here to several cases that could cause confusion. Other details can be found in Table 2.

**Table 2:** Human ANTP class homeobox genes and pseudogenes

Human ANTP-class homeobox genes and pseudogenes					
**HOXL subclass**					
**Family**	**Gene symbol**	**Gene name**	**Location**	**Entrez gene ID**	**Previous symbols**

**Cdx**	*CDX1*	caudal type homeobox 1	5q32	1044	
	*CDX2*	caudal type homeobox 2	13q12.2	1045	CDX3
	*CDX4*	caudal type homeobox 4	Xq13.2	1046	
**Evx**	*EVX1*	even-skipped homeobox 1	7p15.2	2128	
	*EVX2*	even-skipped homeobox 2	2q31.1	344191	
**Gbx**	*GBX1*	gastrulation brain homeobox 1	7q36.1	2636	
	*GBX2*	gastrulation brain homeobox 2	2q37.2	2637	
**Gsx**	*GSX1*	GS homeobox 1	13q12.2	219409	GSH1
	*GSX2*	GS homeobox 2	4q12	170825	GSH2
**Hox1**	*HOXA1*	homeobox A1	7p15.2	3198	HOX1F
	*HOXB1*	homeobox B1	17q21.32	3211	HOX2I
	*HOXD1*	homeobox D1	2q31.1	3231	HOX4G
**Hox2**	*HOXA2*	homeobox A2	7p15.2	3199	HOX1K
	*HOXB2*	homeobox B2	17q21.32	3212	HOX2H
**Hox3**	*HOXA3*	homeobox A3	7p15.2	3200	HOX1E
	*HOXB3*	homeobox B3	17q21.32	3213	HOX2G
	*HOXD3*	homeobox D3	2q31.1	3232	HOX4A
**Hox4**	*HOXA4*	homeobox A4	7p15.2	3201	HOX1D
	*HOXB4*	homeobox B4	17q21.32	3214	HOX2F
	*HOXC4*	homeobox C4	12q13.13	3221	HOX3E
	*HOXD4*	homeobox D4	2q31.1	3233	HOX4B
**Hox5**	*HOXA5*	homeobox A5	7p15.2	3202	HOX1C
	*HOXB5*	homeobox B5	17q21.32	3215	HOX2A
	*HOXC5*	homeobox C5	12q13.13	3222	HOX3D
**Hox6-8**	*HOXA6*	homeobox A6	7p15.2	3203	HOX1B
	*HOXB6*	homeobox B6	17q21.32	3216	HOX2B
	*HOXC6*	homeobox C6	12q13.13	3223	HOX3C
	*HOXA7*	homeobox A7	7p15.2	3204	HOX1A
	*HOXB7*	homeobox B7	17q21.32	3217	HOX2C
	*HOXB8*	homeobox B8	17q21.32	3218	HOX2D
	*HOXC8*	homeobox C8	12q13.13	3224	HOX3A
	*HOXD8*	homeobox D8	2q31.1	3234	HOX4E
**Hox9-13**	*HOXA9*	homeobox A9	7p15.2	3205	HOX1G
	*HOXB9*	homeobox B9	17q21.32	3219	HOX2E
	*HOXC9*	homeobox C9	12q13.13	3225	HOX3B
	*HOXD9*	homeobox D9	2q31.1	3235	HOX4C
	*HOXA10*	homeobox A10	7p15.2	3206	HOX1H
	*HOXC10*	homeobox C10	12q13.13	3226	HOX3I
	*HOXD10*	homeobox D10	2q31.1	3236	HOX4D, HOX4E
	*HOXA11*	homeobox A11	7p15.2	3207	HOX1I
	*HOXC11*	homeobox C11	12q13.13	3227	HOX3H
	*HOXD11*	homeobox D11	2q31.1	3237	HOX4F
	*HOXC12*	homeobox C12	12q13.13	3228	HOX3F
	*HOXA13*	homeobox A13	7p15.2	3209	HOX1J
	*HOXB13*	homeobox B13	17q21.32	10481	
	*HOXC13*	homeobox C13	12q13.13	3229	HOX3G
	*HOXD13*	homeobox D13	2q31.1	3239	HOX4I
**Mnx**	*MNX1*	motor neuron and pancreas homeobox 1	7q36.3	3110	HLXB9, HB9, HOXHB9
**Meox**	*MEOX1*	mesenchyme homeobox 1	17q21.31	4222	MOX1
	*MEOX2*	mesenchyme homeobox 2	7p21.1	4223	MOX2, GAX
**Pdx**	*PDX1*	pancreatic and duodenal homeobox 1	13q12.2	3651	IPF1, IUF1, IDX1, STF1

**NKL subclass**					

**Barhl**	*BARHL1*	BarH-like homeobox 1	9q34.13	56751	
	*BARHL2*	BarH-like homeobox 2	1p22.2	343472	
**Barx**	*BARX1*	BARX homeobox 1	9q22.32	56033	
	*BARX2*	BARX homeobox 2	11q24.3	8538	
**Bsx**	*BSX*	brain specific homeobox	11q24.1	390259	
**Dbx**	*DBX1*	developing brain homeobox 1	11p15.1	120237	
	*DBX2*	developing brain homeobox 2	12q12	440097	
**Dlx**	*DLX1*	distal-less homeobox 1	2q31.1	1745	
	*DLX2*	distal-less homeobox 2	2q31.1	1746	TES1
	*DLX3*	distal-less homeobox 3	17q21.33	1747	
	*DLX4*	distal-less homeobox 4	17q21.33	1748	DLX7, DLX8, DLX9, BP1
	*DLX5*	distal-less homeobox 5	7q21.3	1749	
	*DLX6*	distal-less homeobox 6	7q21.3	1750	
**Emx**	*EMX1*	empty spiracles homeobox 1	2p13.2	2016	
	*EMX2*	empty spiracles homeobox 2	10q26.11	2018	
**En**	*EN1*	engrailed homeobox 1	2q14.2	2019	
	*EN2*	engrailed homeobox 2	7q36.3	2020	
**Hhex**	*HHEX*	hematopoietically expressed homeobox	10q23.33	3087	HEX, PRH, PRHX
**Hlx**	*HLX*	H2.0-like homeobox	1q41	3142	HLX1, HB24
**Lbx**	*LBX1*	ladybird homeobox 1	10q24.32	10660	LBX1H, HPX6
	*LBX2*	ladybird homeobox 2	2p13.1	85474	
**Msx**	*MSX1*	msh homeobox 1	4p16.2	4487	HOX7
	*MSX2*	msh homeobox 2	5q35.2	4488	HOX8, MSH
	*MSX2P1*	msh homeobox 2 pseudogene	17q23.2	55545	HPX5, MSX2P
**Nanog**	*NANOG*	Nanog homeobox	12p13.31	79923	
	*NANOGP1*	Nanog homeobox pseudogene 1	12p13.31	404635	NANOG2
	*NANOGP2*	Nanog homeobox pseudogene 2	2q36.1	414131	NANOGP4
	*NANOGP3*	Nanog homeobox pseudogene 3	6p12.1	340217	
	*NANOGP4*	Nanog homeobox pseudogene 4	7p15.1	414132	NANOGP2
	*NANOGP5*	Nanog homeobox pseudogene 5	9q31.1	414133	
	*NANOGP6*	Nanog homeobox pseudogene 6	10q24.2	414134	
	*NANOGP7*	Nanog homeobox pseudogene 7	14q32.12	414130	NANOGP3
	*NANOGP8*	Nanog homeobox pseudogene 8	15q14	388112	NANOGP1
	*NANOGP9*	Nanog homeobox pseudogene 9	Xq12	349386	NANOGP6
	*NANOGP10*	Nanog homeobox pseudogene 10	Xp11.3	349372	NANOGP5
	*NANOGP11*	Nanog homeobox pseudogene 11	6q25.2	414135	
**Nk1**	*NKX1-1*	NK1 homeobox 1	4p16.3	54279	NKX1.1, HSPX153, HPX153
	*NKX1-2*	NK1 homeobox 2	10q26.13	390010	NKX1.2, C10orf121
**Nk2.1**	*NKX2-1*	NK2 homeobox 1	14q13.3	7080	NKX2.1, NKX2A, TTF1, TITF1
	*NKX2-4*	NK2 homeobox 4	20p11.22	4823	NKX2.4, NKX2D
**Nk2.2**	*NKX2-2*	NK2 homeobox 2	20p11.22	4821	NKX2.2, NKX2B
	*NKX2-8*	NK2 homeobox 8	14q13.3	26257	NKX2.8, NKX2H
**Nk3**	*NKX3-1*	NK3 homeobox 1	8p21.2	4824	NKX3.1, NKX3A
	*NKX3-2*	NK3 homeobox 2	4p15.33	579	NKX3.2, NKX3B, BAPX1
**Nk4**	*NKX2-3*	NK2 homeobox 3	10q24.2	159296	NKX2.3, NKX2C, NKX4-3, CSX3
	*NKX2-5*	NK2 homeobox 5	5q35.1	1482	NKX2.5, NKX2E, NKX4-1, CSX, CSX1
	*NKX2-6*	NK2 homeobox 6	8p21.2	137814	NKX2.6, NKX4-2, CSX2
**Nk5/Hmx**	*HMX1*	H6 family homeobox 1	4p16.1	3166	NKX5-3, H6
	*HMX2*	H6 family homeobox 2	10q26.13	3167	NKX5-2, H6L
	*HMX3*	H6 family homeobox 3	10q26.13	340784	NKX5-1
**Nk6**	*NKX6-1*	NK6 homeobox 1	4q21.23	4825	NKX6.1, NKX6A
	*NKX6-2*	NK6 homeobox 2	10q26.3	84504	NKX6.2, NKX6B, GTX
	*NKX6-3*	NK6 homeobox 3	8p11.21	157848	NKX6.3
**Noto**	*NOTO*	notochord homeobox	2p13.2	344022	
**Tlx**	*TLX1*	T-cell leukemia homeobox 1	10q24.32	3195	HOX11, TCL3
	*TLX2*	T-cell leukemia homeobox 2	2p13.1	3196	HOX11L1, NCX
	*TLX3*	T-cell leukemia homeobox 3	5q35.1	30012	HOX11L2, RNX
**Vax**	*VAX1*	ventral anterior homeobox 1	10q26.11	11023	
	*VAX2*	ventral anterior homeobox 2	2p13.3	25806	
**Ventx**	*VENTX*	VENT homeobox	10q26.3	27287	VENTX2, HPX42B
	*VENTXP1*	VENT homeobox pseudogene 1	Xp21.3	139538	VENTX2P1, NA88A
	*VENTXP2*	VENT homeobox pseudogene 2	13q31.1	347975	VENTX2P2
	*VENTXP3*	VENT homeobox pseudogene 3	12q21.1	349814	VENTX2P3
	*VENTXP4*	VENT homeobox pseudogene 4	3p24.2	152101	VENTX2P4
	*VENTXP5*	VENT homeobox pseudogene 5	8p12	442384	
	*VENTXP6*	VENT homeobox pseudogene 6	8q21.11	552879	
	*VENTXP7*	VENT homeobox pseudogene 7	3p24.3	391518	VENTX1, HPX42

Human ANTP class homeobox genes and pseudogenes including full names, chromosomal locations, Entrez Gene IDs and previous symbols. *NANOGP1 *is a duplicate of *NANOG*.

∘ Cdx, Gsx and Pdx gene families. Some authors refer to the Pdx gene family as the Xlox gene family [28]. One gene from each of these families (*CDX2*, *GSX1 *and *PDX1*) forms the ParaHox cluster at 13q12.2 (Figure 4), and clustering of Cdx, Gsx and Pdx genes is ancestral for chordates [28].

∘ Mnx gene family. This gene family name derives from a previous study [29]. The family includes one gene in the human genome: *MNX1 *(formerly *HLXB9*), and two genes in the chicken genome: *Mnx1 *(formerly *HB9*) and *Mnx2 *(formerly *MNR2*). Some authors refer to the Mnx gene family as the Exex gene family due to the *Drosophila *ortholog *exex *[7].

∘ Dlx gene family. It is currently unclear if this gene family is derived from one or more genes in the common ancestor of bilaterians [18]. Phylogenetic analyses place this gene family firmly within the NKL subclass (Figure 1; Additional file 3), but chromosomal positions (on the Hox chromosomes 2, 7 and 17) place it within the HOXL subclass (Figure 4). Here we favor placement of the Dlx gene family within the NKL subclass due to strong phylogenetic support.

∘ En gene family. Phylogenetic analyses place this gene family either within the NKL subclass (maximum likelihood; Figure 1) or close to the division between the NKL and HOXL subclasses (neighbor-joining; Additional file 3). Here we place the En gene family within the NKL subclass, although we note that human *EN2 *maps close to the clear HOXL-subclass genes *GBX1 *and *MNX1 *on chromosome 7 (Figure 4).

∘ Nk2.1 and Nk2.2 gene families. The genes *NKX2-1 *(formerly *TITF1*), *NKX2-4*, *NKX2-2 *and *NKX2-8 *divide into two distinct gene families each with an invertebrate ortholog, not a single Nk2 gene family. *NKX2-1 *and *NKX2-4 *are collectively orthologous to *Drosophila scro *and amphioxus *AmphiNk2-1 *[30,31]; these comprise one gene family: Nk2.1. *NKX2-2 *and *NKX2-8 *are collectively orthologous to *Drosophila vnd *and amphioxus *AmphiNk2-2 *[31,32]; these comprise a second gene family: Nk2.2.

∘ Nk4 gene family. The genes *NKX2-3*, *NKX2-5 *and *NKX2-6 *form a gene family, quite distinct from other human genes that confusingly share the prefix *NKX2*. These three genes are actually orthologs of *Drosophila tin *(formerly *NK4*); they are not orthologs of *Drosophila vnd *(formerly *NK2*) or *scro *[33]. Therefore, they do not belong to the Nk2.1 or Nk2.2 gene families, but belong to a separate Nk4 gene family. As the three gene names have very extensive current usage, it may be difficult for revised names to be used consistently. In this situation, we don't alter the current names, but raise for discussion the possibility of these genes being renamed to the more logical *NKX4-1 *(*NKX2-5*), *NKX4-2 *(*NKX2-6*) and *NKX4-3 *(*NKX2-3*), or to *CSX1 *(*NKX2-5*), *CSX2 *(*NKX2-6*) and *CSX3 *(*NKX2-3*), based on the alternative name *CSX1 *for *NKX2-5 *[34].

∘ Noto gene family. This gene family falls close to the division between the ANTP and PRD classes in phylogenetic analyses (Additional files 1 and 2). We favor placement within the ANTP class as the human *NOTO *gene is chromosomally linked to the clear ANTP-class (NKL-subclass) genes *EMX1*, *LBX2*, *TLX2 *and *VAX2 *on chromosome 2 (Figure 4), suggesting ancestry by ancient tandem duplication.

Most of the 100 genes in the ANTP class have been adequately named previously. However, several genes were unnamed or misnamed prior to this study. We have updated these as follows.

∘ *GSX2 *[Entrez Gene ID: 170825] is the second of two human members of the Gsx gene family. This previously unnamed gene has clear orthology to mouse *Gsh2*, inferred from sequence identity and synteny. We designate the gene *GSX2 *and revise the nomenclature of the other human member of the family from *GSH1 *to *GSX1 *[Entrez Gene ID: 219409], in accordance with homeobox gene nomenclature convention.

∘ *MNX1 *[Entrez Gene ID: 3110] is the only member of the Mnx gene family in the human genome. This gene was previously known as *HLXB9*; we rename it *MNX1 *because it is not part of a series of at least nine related genes.

∘ *PDX1 *[Entrez Gene ID: 3651] is the only member of the Pdx gene family in the human genome. This gene was previously known as *IPF1*; we rename it *PDX1 *because the majority of published studies use this as the gene symbol.

∘ *BSX *[Entrez Gene ID: 390259] is the only member of the Bsx gene family in the human genome. We designate this previously unnamed gene *BSX *on the basis of clear orthology to the mouse *Bsx *gene, inferred from sequence identity and synteny.

∘ *DBX1 *[Entrez Gene ID: 120237] and *DBX2 *[Entrez Gene ID: 440097] are the only two members of the Dbx gene family in the human genome. We designate these previously unnamed genes *DBX1 *and *DBX2 *on the basis of clear orthology to mouse *Dbx1 *and *Dbx2*, inferred from sequence identity and synteny.

∘ *NKX1-1 *[Entrez Gene ID: 54729] and *NKX1-2 *[Entrez Gene ID: 390010] are the only two members of the Nk1 gene family in the human genome. These genes were previously known as *HSPX153 *and *C10orf121 *respectively; we rename them *NKX1-1 *and *NKX1-2 *on the basis of clear orthology to mouse *Nkx1-1 *and *Nkx1-2*, inferred from sequence identity and synteny.

∘ *NKX2-1 *[Entrez Gene ID: 7080] is the first of two human members of the Nk2.1 gene family. This gene was previously known as *TITF1*; we rename it *NKX2-1 *to show that it is a member of the Nk2.1 gene family.

∘ *NKX2-6 *[Entrez Gene ID: 137814] is the third of three human members of the Nk4 gene family. We designate this previously unnamed gene *NKX2-6 *on the basis of clear orthology to mouse *Nkx2-6*, inferred from sequence identity and synteny, although nomenclature revision for the entire Nk4 gene family should be discussed (see above).

∘ *NKX3-2 *[Entrez Gene ID: 579] is the second of two human members of the Nk3 gene family. This gene was previously known as *BAPX1*; we rename it *NKX3-2 *to show that it is a member of the Nk3 gene family.

∘ *NKX6-3 *[Entrez Gene ID: 157848] is the third of three human members of the Nk6 gene family. We designate this previously unnamed gene *NKX6-3 *on the basis of clear orthology to mouse *Nkx6-3*, inferred from sequence identity and synteny.

∘ *VENTX *[Entrez Gene ID: 27287] is the only functional member of the Ventx gene family in the human genome. This gene was previously known as *VENTX2*. We remove the numerical suffix from this gene symbol because we discovered that the sequence formerly known as *VENTX1 *is actually a retrotransposed pseudogene derived from this gene. Accordingly, we also replace the *VENTX1 *symbol with *VENTXP7 *(see below).

In contrast to the previous descriptions of probable functional genes, there has been much less research on pseudogenes within the ANTP class. Eleven pseudogenes derived from the human *NANOG *gene have been described previously [22], while four pseudogenes in the Ventx gene family have been reported following routine annotation of the human genome. We have identified two additional Ventx-family pseudogenes (*VENTXP5 *and *VENTXP6*), and also found two cases of pseudogenes that were originally mistaken for functional genes (*MSX2P1 *and *VENTXP7*). In all cases, we have clarified the origins and organization of these pseudogenes. This research brings the total number of ANTP-class pseudogenes in the human genome to 19.

∘ *MSX2P1 *[Entrez Gene ID: 55545]. A short cDNA sequence [EMBL: X74862] related to the Msx gene family was reported previously [35]; the former Entrez Gene record labeled *HSHPX5 *was based on this sequence. This locus was later provisionally called *MSX4*, as it was distinct from human *MSX1 *and *MSX2*, and by synteny it was clearly not the ortholog of mouse *Msx3 *[27]. It is now clear that this locus was formed by retrotransposition of mRNA from *MSX2 *and hence we name it *MSX2P1*. The genomic sequence of *MSX2P1 *can now be accessed via the Reference Sequence collection [RefSeq: NR_002307]. The pseudogene shares 91% sequence identity with *MSX2 *mRNA, lacks intronic sequence, and has remnants of a 3' poly(A) tail. It is intriguing, but probably coincidental, that the *MSX2P1 *pseudogene has integrated at 17q23.2, close to several ANTP-class genes (HOXB cluster, *MEOX1*, *DLX3 *and *DLX4*).

∘ *NANOGP1 *[Entrez Gene ID: 404635]. We follow Booth and Holland [22] and classify *NANOGP1 *as a pseudogene that arose by tandem duplication of *NANOG*. The alternative view, argued by Hart et al [36], is that this locus is a functional gene, and should be named *NANOG2*. There is evidence for transcription of this locus in human embryonic stem cells [36], and for selection-driven conservation of the open reading frame [37], but as yet no clear evidence for function.

∘ *NANOGP8 *[Entrez Gene ID: 388112]. We follow Booth and Holland [22] and classify *NANOGP8 *as a retrotransposed pseudogene. The alternative view, argued by Zhang et al [38], is that this locus is a functional retrogene. There is evidence for transcription and translation of this locus in cancer cell lines and tumors [38], but no evidence yet for a role in normal tissues.

∘ *VENTXP1 *[Entrez Gene ID: 139538], *VENTXP2 *[Entrez Gene ID: 347975], *VENTXP3 *[Entrez Gene ID: 349814] and *VENTXP4 *[Entrez Gene ID: 152101]. These four *VENTX *retrotransposed pseudogenes have been reported previously, and were originally known as *VENTX2P1 *to *VENTX2P4*. The correction of the *VENTX2 *gene symbol to simply *VENTX *(see above) means that each of the pseudogene names should also change; we rename them *VENTXP1 *to *VENTXP4*. *VENTXP1 *is transcribed but due to mutations it can no longer encode a homeodomain protein; it can however encode an antigenic peptide (NA88A) responsible for T-cell stimulation in response to melanoma [39].

∘ *VENTXP5 *[Entrez Gene ID: 442384]. We designate this previously unnamed sequence *VENTXP5 *because it is clearly a retrotransposed pseudogene of *VENTX*. The genomic sequence of *VENTXP5 *can now be accessed via the Reference Sequence collection [RefSeq: NG_005091]. The pseudogene shares 83% identity with *VENTX *mRNA (after masking of an Alu element in the parental mRNA sequence), lacks intronic sequence, and has remnants of a 3' poly(A) tail.

∘ *VENTXP6 *[Entrez Gene ID: 552879]. We designate this previously unannotated sequence *VENTXP6 *because it is clearly a retrotransposed pseudogene of *VENTX*. Its lack of annotation may reflect the fact that it is located within an intron of an unrelated and well characterized gene, *STAU2*. The genomic sequence of *VENTXP6 *can now be accessed via the Reference Sequence collection [RefSeq: NG_005090]. The pseudogene shares 87% identity with *VENTX *mRNA (after masking of an Alu element in the parental mRNA sequence) and lacks intronic sequence.

∘ *VENTXP7 *[Entrez Gene ID: 391518]. A short cDNA sequence [EMBL: X74864] was reported previously and named *HPX42 *[35]. This was later renamed the *VENTX1 *gene, after it was found to be related to *Xenopus *Ventx-family genes. Our analysis of the genomic sequence at this locus reveals that it is actually a retrotransposed pseudogene of the *VENTX *gene (formerly *VENTX2*); thus we designate it *VENTXP7*. The genomic sequence of *VENTXP7 *can now be accessed via the Reference Sequence collection [RefSeq: NR_002311]. The pseudogene shares 86% identity with *VENTX *mRNA (after masking of an Alu element in the parental mRNA sequence), lacks intronic sequence, and has remnants of a 3' poly(A) tail.

One other gene could conceivably be included in the ANTP class, but is excluded from our survey. This gene [Entrez Gene ID: 360030; GenBank: AY151139], has been annotated as a homeobox gene and is located just 20 kb from *NANOG*. However, no homeodomain was detected when the deduced protein was analyzed for conserved domains. Also, secondary structure prediction did not predict the expected organisation of alpha helices. Alignment with the NANOG homeodomain reveals identity of the KQ and WF motifs, either side of the same intron position (44/45), but few other shared residues. It is possible, but unproven, that the locus arose by tandem duplication of part, or all, of the *NANOG *homeobox gene. This gene has generated two retrotransposed pseudogenes: one at 2q11.2 and another at 12q24.33.

### The PRD homeobox class

The PRD class derives its name from the *paired *(*prd*) gene of *Drosophila melanogaster*. In previous studies, the PRD class has been subdivided in several different ways, often based on identify of the amino acid at residue 50 in the homeodomain, for example S50, K50 and Q50. These categories are not monophyletic groupings of genes and so can be misleading if we aim for a classification scheme that reflects evolution [5]. Here we divide the PRD class into two subclasses of unequal size: the PAX subclass (containing seven PAX genes, excluding *PAX1 *and *PAX9*), and the PAXL subclass (containing 43 non-PAX genes and many pseudogenes) (Table 1). PAX genes are defined by possession of a conserved paired-box motif, distinct from the homeobox, coding for the 128-amino-acid PRD domain. Of the nine human genes possessing a paired-box (*PAX1 *to *PAX9*), only four also contain a complete homeobox (*PAX3*, *PAX7*, *PAX4 *and *PAX6*). Three genes have a partial homeobox (*PAX2*, *PAX5 *and *PAX8*), while two lack a homeobox entirely (*PAX1 *and *PAX9*). Phylogenetic analyses using PAX genes from a range of species suggest that these are secondary conditions, and that the ancestral PAX gene probably possessed both motifs [40]. The PAX genes do not constitute a single gene family, because it is clear that the latest common ancestor of the Bilateria contained four PAX genes. Three of these are ancestors of the PRD-class homeobox gene families Pax2/5/8, Pax3/7 and Pax4/6; the fourth is the ancestor of *PAX1 *and *PAX9*. Thus the PAX subclass contains three gene families. We divide the PAXL subclass into 28 gene families, although as explained below not all of these date to the base of the Bilateria. Thus, we recognize a total of 31 gene families in the PRD class (Table 1).

Many of the 31 gene families in the PRD class have been clearly defined before. We draw attention here to newly defined gene families and cases that could cause confusion. Other details can be found in Table 3.

**Table 3:** Human PRD class homeobox genes and pseudogenes

Human PRD-class homeobox genes and pseudogenes					
**Family**	**Gene symbol**	**Gene name**	**Location**	**Entrez gene IDc**	**Previous symbols**
**Alx**	*ALX1*	ALX homeobox 1	12q21.31	8092	CART1
	*ALX3*	ALX homeobox 3	1p13.3	257	
	*ALX4*	ALX homeobox 4	11p11.2	6059	
**Argfx**	*ARGFX*	arginine-fifty homeobox	3q13.33	503582	
	*ARGFXP1*	arginine-fifty homeobox pseudogene 1	5q23.2	503583	
	*ARGFXP2*	arginine-fifty homeobox pseudogene 2	17q11.2	503640	
**Arx**	*ARX*	aristaless related homeobox	Xp21.3	170302	ISSX
**Dmbx**	*DMBX1*	diencephalon/mesencephalon brain homeobox 1	1p34.1	127343	MBX, OTX3, PAXB
**Dprx**	*DPRX*	divergent paired-related homeobox	19q13.42	503834	
	*DPRXP1*	divergent paired-related homeobox pseudogene 1	2q32.1	503641	
	*DPRXP2*	divergent paired-related homeobox pseudogene 2	6p21.31	503643	
	*DPRXP3*	divergent paired-related homeobox pseudogene 3	14q13.2	503644	
	*DPRXP4*	divergent paired-related homeobox pseudogene 4	17q11.2	503645	
	*DPRXP5*	divergent paired-related homeobox pseudogene 5	21q22.13	503646	
	*DPRXP6*	divergent paired-related homeobox pseudogene 6	Xp11.4	503647	
	*DPRXP7*	divergent paired-related homeobox pseudogene 7	Xq23	503648	
**Drgx**	*DRGX*	dorsal root ganglia homeobox	10q11.23	644168	DRG11, PRRXL1
**Dux**	*DUXA*	double homeobox A	19q13.43	503835	
	*DUXAP1*	double homeobox A pseudogene 1	2p11.2	503630	
	*DUXAP2*	double homeobox A pseudogene 2	8q22.3	503631	
	*DUXAP3*	double homeobox A pseudogene 3	10q11.21	503632	
	*DUXAP4*	double homeobox A pseudogene 4	10q11.21	503633	
	*DUXAP5*	double homeobox A pseudogene 5	11q23.3	503634	
	*DUXAP6*	double homeobox A pseudogene 6	15q26.1	503635	
	*DUXAP7*	double homeobox A pseudogene 7	20p11.23	503636	
	*DUXAP8*	double homeobox A pseudogene 8	22q11.21	503637	
	*DUXAP9*	double homeobox A pseudogene 9	14qcen	503638	
	*DUXAP10*	double homeobox A pseudogene 10	14q11.2	503639	
	*DUXB*	double homeobox B	16q23.1	100033411	
**Esx**	*ESX1*	ESX homeobox 1	Xq22.2	80712	ESX1L, ESXR1
**Gsc**	*GSC*	goosecoid homeobox	14q32.13	145258	GSC1
	*GSC2*	goosecoid homeobox 2	22q11.21	2928	GSCL
**Hesx**	*HESX1*	HESX homeobox 1	3p14.3	8820	RPX, ANF
**Hopx**	*HOPX*	HOP homeobox	4q12	84525	HOP, OB1, LAGY, NECC1, SMAP31
**Isx**	*ISX*	intestine specific homeobox	22q12.3	91464	RAXLX
**Leutx**	*LEUTX*	Leucine twenty homeobox	19q13.2	342900	
**Mix**	*MIXL*	Mix paired-like homeobox	1q42.12	83881	MIX, MIXL1, MILD1
**Nobox**	*NOBOX*	NOBOX oogenesis homeobox	7q35	135935	OG2, OG2X
**Otp**	*OTP*	orthopedia homeobox	5q14.1	23440	
**Otx**	*OTX1*	orthodenticle homeobox 1	2p15	5013	
	*OTX2*	orthodenticle homeobox 2	14q22.3	5015	
	*OTX2P1*	orthodenticle homeobox 2 pseudogene	9q21.2	100033409	OTX2P
	*CRX*	cone-rod homeobox	19q13.32	1406	OTX3
**Pax2/5/8**	*PAX2*	paired box 2	10q24.31	5076	
	*PAX5*	paired box 5	9p13.2	5079	BSAP
	*PAX8*	paired box 8	2q13	7849	
**Pax3/7**	*PAX3*	paired box 3	2q36.1	5077	HUP2
	*PAX7*	paired box 7	1p36.13	5081	HUP1, PAX7B
**Pax4/6**	*PAX4*	paired box 4	7q32.1	5078	
	*PAX6*	paired box 6	11p13	5080	
**Phox**	*PHOX2A*	paired-like homeobox 2a	11q13.4	401	PMX2A, ARIX
	*PHOX2B*	paired-like homeobox 2b	4p13	8929	PMX2B, NBPhox
**Pitx**	*PITX1*	pituitary homeobox 1	5q31.1	5307	PTX1, POTX, BFT
	*PITX2*	pituitary homeobox 2	4q25	5308	PTX2, ARP1, RGS, RIEG, RIEG1
	*PITX3*	pituitary homeobox 3	10q24.32	5309	PTX3
**Prop**	*PROP1*	PROP paired-like homeobox 1	5q35.3	5626	
**Prrx**	*PRRX1*	paired related homeobox 1	1q24.3	5396	PRX1, PMX1, PHOX1
	*PRRX2*	paired related homeobox 2	9q34.11	51450	PRX2, PMX2
**Rax**	*RAX*	retina and anterior neural fold homeobox	18q21.31	30062	RX
	*RAX2*	retina and anterior neural fold homeobox 2	19p13.3	84839	QRX, RAXL1
**Rhox**	*RHOXF1*	Rhox homeobox family, member 1	Xq24	158800	PEPP1, OTEX
	*RHOXF2*	Rhox homeobox family, member 2	Xq24	84528	PEPP2
	*RHOXF2B*	Rhox homeobox family, member 2B	Xq24	727940	PEPP2L
**Sebox**	*SEBOX*	SEBOX homeobox	17q11.2	645832	OG9, OG9X
**Shox**	*SHOX*	short stature homeobox	Xp22.33/ Yp11.32	6473	SHOXY, GCFX, PHOG
	*SHOX2*	short stature homeobox 2	3q25.32	6474	SHOT, OG12, OG12X
**Tprx**	*TPRX1*	tetra-peptide repeat homeobox 1	19q13.32	284355	
	*TPRX2P*	tetra-peptide repeat homeobox 2 pseudogene	19q13.32	503627	
	*TPRX1P1*	tetra-peptide repeat homeobox 1 pseudogene 1	10q22.3	503628	
	*TPRX1P2*	tetra-peptide repeat homeobox 1 pseudogene 2	10q22.3	503629	
	*TPRXL*	tetra-peptide repeat homeobox-like	3p25.1	348825	
**Uncx**	*UNCX*	UNC homeobox	7p22.3	340260	PHD1, UNCX4.1
**Vsx**	*VSX1*	visual system homeobox 1	20p11.21	30813	KTCN, RINX
	*VSX2*	visual system homeobox 2	14q24.3	338917	RET1, HOX10, CHX10

Human PRD class homeobox genes and pseudogenes including full names, chromosomal locations, Entrez Gene IDs and previous symbols. Pax2/5/8-family genes contain a partial homeobox. *RHOXF2B *is a duplicate of *RHOXF2*. *TPRX2P *is a duplicate of *TPRX1*.

∘ Argfx, Dprx and Tprx gene families. There are no known invertebrate members of these three gene families. Therefore, these are exceptions to the rule defining gene families as dating to the base of the Bilateria. The Dprx and Tprx gene families may have arisen by duplication and very extensive divergence from *CRX*, a member of the Otx gene family, during mammalian evolution; origins of *ARGFX *are obscure [21].

∘ Dux gene family. Members of this gene family are characterized by the presence of two closely-linked homeobox motifs. Most members are intronless sequences present in multiple polymorphic copies within the 3.3 kb family of tandemly repeated elements associated with heterochromatin. These comprise the sequences known as *DUX1 *to *DUX5 *reported in previous studies [12-14] and numerous *DUX4 *copies detected in this study (see below). The absence of introns suggests that these sequences may have originated by retrotransposition from an mRNA transcript, thus they are probably non-functional. There are two noticeable exceptions; these members known as *DUXA *and *DUXB *possess introns, thus either one could be the progenitor for the large number of intronless Dux-family sequences found in the human genome. *DUXA *has spawned 10 retrotransposed pseudogenes and has been described previously [21]. *DUXB *is described here (see below).

∘ Hopx gene family. Phylogenetic analyses places this gene family, containing a single very divergent homeobox gene *HOPX *(formerly *HOP*), either within the PRD class (maximum likelihood; Additional file 1) or close to Zhx/Homez-family genes (neighbor-joining; Additional file 2). We favor placement in the PRD class for three reasons. First, the HOPX homeodomain has highest sequence identity with PRD-class homeodomains (GSC: 38% and PAX6: 36%). Second, the HOPX homeodomain possesses the same combination of residues that are invariably conserved across human PRD-class homeodomains (Additional file 6). Third, the HOPX homeodomain shares the 46/47 intron position seen in many PRD-class homeodomains. *HOPX *does not map particularly near any other homeobox genes, although the closest is *GSX2 *in the ANTP class at 4q12 (Figure 4). *HOPX *is not a typical PRD-class homeobox gene; the homeodomain has a single amino acid insertion between helix I and helix II (Additional file 6), and lacks the ability to bind DNA [41,42].

∘ Leutx gene family. This gene family contains a single gene in the human genome, *LEUTX*, and no known invertebrate members. We place *LEUTX *in the PRD class for four reasons. First, there is weak phylogenetic support for this placement (Additional files 1 and 2). Second, the LEUTX homeodomain possesses the same combination of residues that are invariably conserved across human PRD-class homeodomains (except for a leucine at position 20; Additional file 6). Third, the LEUTX homeodomain shares the 46/47 intron position seen in many PRD-class homeodomains. Fourth, the *LEUTX *gene is located close to the PRD-class genes *TPRX1*, *CRX*, *DPRX *and *DUXA *on the distal end of the long arm of chromosome 19 (Figure 4). This fourth observation leads us to hypothesize that this gene family arose by tandem duplication and extensive divergence during mammalian evolution.

∘ Nobox gene family. This gene family falls close to the division between the ANTP and PRD classes in both maximum likelihood and neighbor-joining phylogenetic analyses (Additional files 1 and 2). We favor placement within the PRD class because the NOBOX homeodomain has higher sequence identity with PRD-class homeodomains (up to 55%) than with ANTP-class homeodomains (up to 46%). Chromosomal position does not shed light on the issue, as its location at 7q35 is close to both ANTP- and PRD-class genes (Figure 4).

∘ Otx gene family. This very well known gene family was originally considered to contain human *OTX1 *and *OTX2 *(and their mouse orthologs) and the *Drosophila otd *gene [43]. Later, it was shown that the *CRX *gene is a member of the same gene family, deriving from the same ancestral gene. Thus, *CRX *could be considered the true *OTX3 *gene [44]. Unfortunately, the *OTX3 *symbol was formerly used erroneously for a gene in a different family, now called *DMBX1*, thus complicating its future use. The gene family name Otx is derived by majority rule from the constituent genes.

∘ Pax2/5/8 gene family. This gene family is also known as Pax group II; it contains *PAX2*, *PAX5 *and *PAX8*, clearly derived from a single ancestral gene [45]. These genes have partial homeoboxes.

∘ Pax3/7 gene family. This gene family is also known as Pax group III; it contains *PAX3 *and *PAX7*, clearly derived from a single ancestral gene [46].

∘ Pax4/6 gene family. This gene family is also known as Pax group IV; it contains *PAX4 *and *PAX6*. There is confusion as to whether this should be split into two gene families, because invertebrate homologs generally group with *PAX6 *in phylogenetic analyses and not as an outgroup to the two genes as might be expected. We follow the generally accepted view and group *PAX4 *and *PAX6 *into a single gene family, proposing that *PAX4 *is a divergent member, not an ancient gene [40].

∘ Rhox gene family. The mouse Rhox cluster was first described as comprising twelve X-linked homeobox genes, all selectively expressed in reproductive tissues [47]. Subsequent studies reported a total of 32 genes in the cluster, with the additional genes attributed to recent tandem duplications [48-51]. The human genome contains three homeobox genes at Xq24 that are clearly members of the Rhox gene family based on sequence identity, molecular phylogenetics, intron positions and chromosomal location. These are *RHOXF1 *(formerly *OTEX/PEPP1*), *RHOXF2 *(formerly *PEPP2*) and *RHOXF2B *(formerly *PEPP2b/PEPP3*).

Most of the 50 genes in the PRD class have been adequately named previously. However, several genes were unnamed or misnamed prior to this study. We have updated these as follows.

∘ *ALX1 *[Entrez Gene ID: 8092] is the first of three human members of the Alx gene family. This gene was previously known as *CART1*; we rename it *ALX1 *because it is related to *ALX3 *and *ALX4*; all three genes were formed by duplication from a single ancestral invertebrate gene [52].

∘ *DRGX *[Entrez Gene ID: 117065] is the only member of the newly defined Drgx gene family in the human genome. This gene was previously known as *PRRXL1 *and *DRG11*, and there is a clear mouse ortholog (*Prrxl1*). The symbol *PRRXL1 *is misleading because it infers membership of the Prrx gene family, containing *PRRX1 *and *PRRX2 *in the human genome. Several lines of evidence suggest it belongs to a different gene family. First, this gene (at 10q11.23) is not located in the same paralogon as *PRRX1 *(1q24.3) and *PRRX2 *(9q34.11) so they are not three paralogs generated during genome duplication in early vertebrate evolution. Second, it has a completely different exon-intron structure from the Prrx-family genes, and it does not contain a Prrx domain or an OAR domain (present in *PRRX1 *and *PRRX2*; [53]). Third, the homeodomain is only 73% identical to PRRX1 and PRRX2 homeodomains, much lower than the 80-100% usually encountered for members of the same gene family in humans. Finally, we have identified the *Drosophila *ortholog, *IP09201*. The homeodomains of *Drosophila *IP09201 and human DRGX form a highly supported monophyletic group in our maximum likelihood (90%; Additional file 1) and neighbor-joining (97%; Additional file 2) phylogenetic analyses. The new symbol *DRGX *(*dorsal root ganglia homeobox*) incorporates the root of the former symbol *DRG11*, referring to expression of the rodent ortholog in dorsal root ganglia neurons [54].

∘ *DUXB *[Entrez Gene ID: 100033411] is a human member of the Dux (double homeobox) gene family. As previously discussed, most members of this gene family are intronless and are probably derived by retrotransposition of an mRNA transcript from a functional intron-containing Dux gene (or duplication of such an integrant). Booth and Holland [21] described the *DUXA *gene containing five introns (including one within each homeobox), and noted the existence of a second intron-containing human Dux-family gene provisionally designated *DUXB*. The *DUXB *nomenclature is endorsed here. No cDNA or EST sequences have been reported for *DUXB*.

∘ *GSC2 *[Entrez Gene ID: 2928] is the second of two human members of the Gsc gene family. This gene was previously known as *GSCL*; we rename it *GSC2 *to remove the inadvertent implication that it is not a true gene, and also to reflect the clear orthology to chick *Gsc2 *as inferred by phylogenetic analysis and synteny.

∘ *HOPX *[Entrez Gene ID: 84525] is the only member of the newly defined Hopx gene family in the human genome. The mouse version of the gene was first identified first and named *Hop *(*homeodomain only protein*) because the encoded protein is just 73 amino acids long, with 61 of these making up the homeodomain [41,42]. The *HOP *gene symbol is not ideal as it is also used for unrelated genes, including *hopscotch *in *Drosophila *and *hop-sterile *in mouse. Therefore, we revise the gene symbol from *HOP *to *HOPX *(*HOP homeobox*) in accordance with homeobox gene nomenclature convention.

∘ *LEUTX *[Entrez Gene ID: 342900] is the only member of the newly defined Leutx gene family in the human genome. We designate this previously unnamed gene *LEUTX *(leucine twenty homeobox) to reflect the presence of a leucine residue at the otherwise highly conserved homeodomain position 20; other PRD-class homeodomains have a phenylalanine at this position (Additional file 6). Studies of mutations in other homeobox genes suggest that mutation to leucine alters transcriptional activity of a homeodomain protein [55].

∘ *RAX2 *[Entrez Gene ID: 84839] is the second of two human members of the Rax gene family. This gene was previously known as *RAXL1*; we rename it *RAX2 *to standardize nomenclature.

∘ *RHOXF1 *[Entrez Gene ID: 158800] and *RHOXF2 *[Entrez Gene ID: 84528] are two of three human members of the Rhox gene family. These genes were previously known as *OTEX/PEPP1 *and *PEPP2 *respectively. The prefix *PEPP *is not suitable as it is used for numerous aminopeptidase P-encoding genes. Thus, we replace the gene symbols *OTEX/PEPP1 *and *PEPP2 *with *RHOXF1 *and *RHOXF2 *respectively, to reflect their orthologous relationship with the mouse Rhox cluster (containing 32 genes, see above) whilst avoiding inadvertent equivalence to specific genes within the cluster.

∘ *RHOXF2B *[Entrez Gene ID: 727940] is the third human member of the Rhox gene family. This locus was referred to in previous studies as *PEPP2b *[56] and *PEPP3 *[51]. The prefix *PEPP *cannot be approved for reasons noted above. *RHOXF2B *is located very close to *RHOXF1 *and *RHOXF2 *at Xq24 and is clearly a very recent duplicate of *RHOXF2*. The genomic sequences at these two loci share 99% identity over exonic, intronic and approximately 20 kb flanking regions. Over the coding region, there are just two nucleotide substitutions (both nonsynonymous); one of these results in an unusual change within the homeodomain (arginine to cysteine at position 18). We currently list *RHOXF2B *as a functional gene, although it is possible that it is a duplicated pseudogene.

∘ *SEBOX *[Entrez Gene ID: 645832] is the only member of the Sebox gene family in the human genome. The human gene is the ortholog of mouse *Sebox *based on their locations in syntenic chromosomal regions (17q11.2 and 11B5 respectively) and presence of the same intron positions. However, sequence identity is lower than normal for orthologous genes in mouse and human (78% amino acid identity over the homeodomain) and there is evidence that the human gene has undergone divergence. Most surprisingly, the human sequence has two unusual substitutions in the homeodomain [57]. At homeodomain position 51, the human sequence codes for lysine whereas mouse has asparagine; an earlier analysis of 346 homeodomain sequences found asparagine to be invariant at this position [1,2]. Similarly, at homeodomain position 53, human has tryptophan whereas mouse has arginine; this position is almost invariably arginine [1,2]. These sequence changes in the important third helix raise the possibility that human *SEBOX *could have accumulated mutations as a non-functional pseudogene. Until this is shown more clearly we consider it to be a functional, but divergent, gene. This gene was previously known as *OG9X *with *SEBOX *as the alternative symbol; we favor *SEBOX *because the *OG *prefix was originally used for several unrelated homeobox genes.

∘ *UNCX *[Entrez Gene ID: 340260] is the only member of the Uncx gene family in the human genome. This gene was previously known as *UNCX4.1*; we remove the numerals to give *UNCX *as these do not denote a series within a gene family.

∘ *VSX2 *[Entrez Gene ID: 338917] is the second of two human members of the Vsx gene family. This gene was previously known as *CHX10*; we rename it *VSX2 *to better reflect its paralogous relationship to *VSX1*. *VSX2 *has been used as an alias for this gene in other vertebrate species and the gene symbol *CHX10 *has the disadvantage of implicitly suggesting presence of at least nine paralogs in human (*CHX1 *to *CHX9*), which do not exist.

Unlike the situation with the ANTP class, many of the pseudogenes within the PRD class have been well characterized. A previous study has described and named two pseudogenes in the Argfx gene family, seven pseudogenes in the Dprx gene family, four pseudogenes in the Tprx gene family, and 10 pseudogenes derived from the *DUXA *gene [21]. There is also a possibility that the *SEBOX *and *RHOXF2B *loci are non-functional pseudogenes, as described above. We have identified a previously undescribed pseudogene from the Otx gene family (*OTX2P1*), and argue that the majority of Dux-family sequences are pseudogenes.

∘ *OTX2P1 *[Entrez Gene ID: 100033409]. We designate this previously undescribed sequence *OTX2P1 *because it is clearly a retrotransposed pseudogene of *OTX2*. The genomic DNA sequence of *OTX2P1 *shares significant homology with *OTX2 *transcript variant 2 [RefSeq: NM_172337]. There is an Alu element (AluSx subfamily) insertion, a Made1 (Mariner derived element 1) insertion, and a 1182-nucleotide deletion in *OTX2P1 *compared to *OTX2*. The *OTX2P1 *sequence lacks introns, ends with a poly(A) tail, and harbors critical sequence alterations (including a three-nucleotide insertion introducing a stop codon into the deduced homeodomain).

∘ *DUX1 *[EMBL: AJ001481], *DUX2 *[GenBank: AF068744], *DUX3 *[GenBank: AF133130] and *DUX5 *[GenBank: AF133131]. These sequences have been cloned in previous studies [12,13]. We detected no matches with 100% identity to *DUX1*, *DUX2*, *DUX3 *or *DUX5 *in build 35.1 of the human genome sequence, which covers the euchromatic regions of each chromosome. This concurs with previous studies indicating that *DUX1*, *DUX2*, *DUX3 *and *DUX5 *are found in heterochromatin on human acrocentric chromosomes; each is apparently present in multiple copies within members of the 3.3 kb family of tandemly repeated DNA elements [12,13]. Because the majority of human heterochromatin has not been sequenced, and may be variable between individuals, the exact number of copies of *DUX1*, *DUX2*, *DUX3 *and *DUX5 *is unknown. It is also debatable whether these loci encode functional proteins. These sequences lack introns and, as discussed above, are most likely derived from intron-containing genes in the Dux family, such as *DUXA *or *DUXB*.

∘ *DUX4 *[GenBank: AF117653]. This sequence has been extensively studied as some of its multiple copies exist within the 3.3 kb repetitive elements of the D4Z4 locus at 4q35 [14]. The polymorphic D4Z4 locus is linked to facioscapulohumeral muscular dystrophy (FSHD); between 12 and 96 tandem copies of 3.3 kb elements are present in unaffected individuals and deletions leaving a maximum of eight such elements have been associated with FSHD [58]. In build 35.1 of the human genome sequence, we identified 35 loci at 10 chromosomal locations containing a total of 58 *DUX4 *(and highly similar) homeobox sequences. This should not be taken as a precise figure due to copy number polymorphism and the possibility of additional copies existing in currently unsequenced heterochromatic regions. Some of the copies are 100% identical to the previously reported *DUX4 *sequence over the homeobox regions, others have single nucleotide polymorphisms, some have critical sequence mutations, and others have just a single homeobox. Most of the copies are located in tandemly repeated arrays (for example, on chromosomes 4, 10 and 16) and others are alone in the genome (for example, a single copy resides at 3p12.3). The majority of *DUX4 *copies are unlikely to encode functional proteins as suggested by their intronless, mutated and tandemly repeated nature. The lack of introns indicates they are most likely derived from intron-containing genes in the Dux family, such as *DUXA *or *DUXB*.

### The LIM homeobox class

The LIM class encodes proteins with two LIM domains (named from the nematode *lin-11*, mammalian *Isl1 *and nematode *mec-3 *genes) N-terminal to a typical (i.e. 60-amino-acid) homeodomain. The LIM domain is a protein-protein interaction domain of approximately 55 amino acids comprising two specialised cysteine-rich zinc fingers in tandem [59]. Importantly, human genes also exist that encode LIM domains but not homeodomains. These LIM domains are divergent from the LIM domains encoded by LIM homeobox genes, and hence these genes are unlikely to be derived by loss of the homeobox. There is one exception: the human Lmo gene family encodes LIM domains that have been grouped by sequence similarity and domain arrangement with the LIM domains of the LIM homeobox gene class [59]. Thus, this gene family may have secondarily lost the homeobox, although this remains untested. Only genes encoding both LIM domains and homeodomains are included in our LIM homeobox gene count.

We have identified a total of twelve LIM-class homeobox genes in the human genome (Tables 1 and 4), consistent with previous work [60]. Phylogenetic analyses of homeodomains do not always recover the LIM class as a monophyletic group, depending on the dataset and method used (Figure 3; Additional files 1, 2 and 5), but it is likely that the class evolved from a single fusion event that brought together LIM domains and a homeodomain. Phylogenetic analyses of homeodomains divide the LIM class into six gene families (Figure 3; Additional files 1, 2 and 5), consistent with previous studies [60]. Each gene family has two human members and dates to a single ancestral gene in the most recent common ancestor of bilaterians [60]. We have not found any human LIM-class pseudogenes.

**Table 4:** Human LIM, POU, HNF, SINE, TALE, CUT, PROS, ZF AND CERS class homeobox genes and pseudogenes

**Human LIM-class homeobox genes**					
**Family**	**Gene symbol**	**Gene name**	**Location**	**Entrez gene ID**	**Previous symbols**

**Isl**	*ISL1*	ISL LIM homeobox 1	5q11.2	3670	
	*ISL2*	ISL LIM homeobox 2	15q24.3	64843	
**Lhx1/5**	*LHX1*	LIM homeobox 1	17q12	3975	LIM1
	*LHX5*	LIM homeobox 5	12q24.13	64211	
**Lhx2/9**	*LHX2*	LIM homeobox 2	9q33.3	9355	LH2
	*LHX9*	LIM homeobox 9	1q31.3	56956	
**Lhx3/4**	*LHX3*	LIM homeobox 3	9q34.3	8022	M2-LHX3
	*LHX4*	LIM homeobox 4	1q25.3	89884	GSH4
**Lhx6/8**	*LHX6*	LIM homeobox 6	9q33.2	26468	LHX6.1
	*LHX8*	LIM homeobox 8	1p31.1	431707	LHX7
**Lmx**	*LMX1A*	LMX LIM homeobox 1A	1q24.1	4009	LMX1, LMX1.1
	*LMX1B*	LMX LIM homeobox 1B	9q33.3	4010	LMX2, LMX1.2

**Human POU-class homeobox genes and pseudogenes**					

**Hdx**	*HDX*	highly divergent homeobox	Xq21.1	139324	CXorf43
**Pou1**	*POU1F1*	POU class 1 homeobox 1	3p11.2	5449	PIT1, GHF1
**Pou2**	*POU2F1*	POU class 2 homeobox 1	1q24.2	5451	OCT1, OTF1
	*POU2F2*	POU class 2 homeobox 2	19q13.2	5452	OCT2, OTF2
	*POU2F3*	POU class 2 homeobox 3	11q23.3	25833	OCT11, PLA1, EPOC1, SKN1A
**Pou3**	*POU3F1*	POU class 3 homeobox 1	1p34.3	5453	OCT6, OTF6, SCIP
	*POU3F2*	POU class 3 homeobox 2	6q16.2	5454	OCT7, OTF7, BRN2, POUF3
	*POU3F3*	POU class 3 homeobox 3	2q12.1	5455	OTF8, BRN1
	*POU3F4*	POU class 3 homeobox 4	Xq21.1	5456	OTF9, BRN4
**Pou4**	*POU4F1*	POU class 4 homeobox 1	13q31.1	5457	BRN3A, RDC1, Oct-T1
	*POU4F2*	POU class 4 homeobox 2	4q31.22	5458	BRN3B, BRN3.2
	*POU4F3*	POU class 4 homeobox 3	5q32	5459	BRN3C
**Pou5**	*POU5F1*	POU class 5 homeobox 1	6p21.33	5460	OCT3, OTF3, OCT4, OTF4
	*POU5F1P1*	POU class 5 homeobox 1 pseudogene 1	8q24.21	5462	OTF3C, OTF3P1, POU5FLC8
	*POU5F1P2*	POU class 5 homeobox 1 pseudogene 2	8q22.3	100009665	
	*POU5F1P3*	POU class 5 homeobox 1 pseudogene 3	12p13.31	642559	OTF3L, POU5F1L, POU5FLC12
	*POU5F1P4*	POU class 5 homeobox 1 pseudogene 4	1q22	645682	POU5FLC1
	*POU5F1P5*	POU class 5 homeobox 1 pseudogene 5	10q21.3	100009667	
	*POU5F1P6*	POU class 5 homeobox 1 pseudogene 6	3q21.3	100009668	
	*POU5F1P7*	POU class 5 homeobox 1 pseudogene 7	3q12.1	100009669	
	*POU5F1P8*	POU class 5 homeobox 1 pseudogene 8	17q25.3	100009670	
	*POU5F2*	POU class 5 homeobox 2	5q15	134187	SPRM1
**Pou6**	*POU6F1*	POU class 6 homeobox 1	12q13.13	5463	BRN5, MPOU, TCFB1
	*POU6F2*	POU class 6 homeobox 2	7p14.1	11281	WT5, WTSL, RPF1

**Human HNF-class homeobox genes**					

**Hmbox**	*HMBOX1*	homeobox containing 1	8p12	79618	HNF1LA, PBHNF
**Hnf1**	*HNF1A*	HNF1 homeobox A	12q24.31	6927	TCF1, HNF1, LFB1
	*HNF1B*	HNF1 homeobox B	17q12	6928	TCF2, LFB3, VHNF1

**Human SINE-class homeobox genes**					

**Six1/2**	*SIX1*	SIX homeobox 1	14q23.1	6495	
	*SIX2*	SIX homeobox 2	2p21	10736	
**Six3/6**	*SIX3*	SIX homeobox 3	2p21	6496	
	*SIX6*	SIX homeobox 6	14q23.1	4990	OPTX2, Six9
**Six4/5**	*SIX4*	SIX homeobox 4	14q23.1	51804	AREC3
	*SIX5*	SIX homeobox 5	19q13.32	147912	DMAHP

**Human TALE-class homeobox genes and pseudogenes**					

**Irx**	*IRX1*	iroquois homeobox 1	5p15.33		IRX-5
	*IRX1P1*	iroquois homeobox 1 pseudogene 1	13q12.12	79192	IRXA1
	*IRX2*	iroquois homeobox 2	5p15.33	646390	
	*IRX3*	iroquois homeobox 3	16q12.2	153572	IRX-1
	*IRX4*	iroquois homeobox 4	5p15.33	50805	
	*IRX4P1*	iroquois homeobox 4 pseudogene 1	18p11.22	100009671	
	*IRX5*	iroquois homeobox 5	16q12.2	79190	IRX2A
	*IRX6*	iroquois homeobox 6	16q12.2		IRX-3, IRX7
**Meis**	*MEIS1*	Meis homeobox 1	2p14	4211	
	*MEIS2*	Meis homeobox 2	15q14	4212	MRG1
	*MEIS3*	Meis homeobox 3	19q13.32	56917	MRG2
	*MEIS3P1*	Meis homeobox 3 pseudogene 1	17p12	4213	MRG2, MEIS3, MEIS4
	*MEIS3P2*	Meis homeobox 3 pseudogene 2	17p11.2	257468	
**Mkx**	*MKX*	mohawk homeobox	10p12.1	283078	IRXL1, IFRX, C10orf48
**Pbx**	*PBX1*	pre-B-cell leukemia homeobox 1	1q23.3	5087	
	*PBX2*	pre-B-cell leukemia homeobox 2	6p21.32	5089	G17, HOX12, PBX2MHC
	*PBX2P1*	pre-B-cell leukemia homeobox 2 pseudogene 1	3q24	5088	PBXP1, PBX2
	*PBX3*	pre-B-cell leukemia homeobox 3	9q33.3	5090	
	*PBX4*	pre-B-cell leukemia homeobox 4	19p13.11	80714	
**Pknox**	*PKNOX1*	PBX/knotted homeobox 1	21q22.3	5316	PREP1, PKNOX1C
	*PKNOX2*	PBX/knotted homeobox 2	11q24.2	63876	PREP2
**Tgif**	*TGIF1*	TGFB-induced factor homeobox 1	18p11.31	7050	TGIF, HPE4
	*TGIF1P1*	TGFB-induced factor homeobox1 pseudogene 1	19q13.32	126052	
	*TGIF2*	TGFB-induced factor homeobox 2	20q11.23	60436	
	*TGIF2P1*	TGFB-induced factor homeobox 2 pseudogene 1	1q44	126826	
	*TGIF2P2*	TGFB-induced factor homeobox 2 pseudogene 2	15q21.1	100009674	
	*TGIF2P3*	TGFB-induced factor homeobox 2 pseudogene 3	15q21.1	100009672	
	*TGIF2P4*	TGFB-induced factor homeobox 2 pseudogene 4	14q24.2	100009673	
	*TGIF2LX*	TGFB-induced factor homeobox 2-like, X-linked	Xq21.31	90316	TGIFLX (retrogene)
	*TGIF2LY*	TGFB-induced factor homeobox 2-like, Y-linked	Yp11.2	90655	TGIFLY (retrogene)

**Human CUT-class homeobox genes and pseudogenes**					

**Onecut**	*ONECUT1*	one cut homeobox 1	15q21.3	3175	HNF6, HNF6A
	*ONECUT2*	one cut homeobox 2	18q21.31	9480	OC2
	*ONECUT3*	one cut homeobox 3	19p13.3	390874	
**Cux**	*CUX1*	cut-like homeobox 1	7q22.1	1523	CUTL1, CUX, CDP, COY1
	*CUX2*	cut-like homeobox 2	12q24.12	23316	CUTL2
	*CUX2P1*	cut-like homeobox 2 pseudogene 1	10p14	-	
	*CUX2P2*	cut-like homeobox 2 pseudogene 2	4q32.1	**-**	
**Satb**	*SATB1*	SATB homeobox 1	3p24.3	6304	
	*SATB2*	SATB homeobox 2	2q33.1	23314	

**Human PROS-class homeobox genes**					

**Prox**	*PROX1*	prospero homeobox 1	1q41	5629	
	*PROX2*	prospero homeobox 2	14q24.3	283571	

**Human ZF-class homeobox genes and pseudogenes**					

**Adnp**	*ADNP*	activity-dependent neuroprotector homeobox	20q13.13	23394	ADNP1
	*ADNP2*	ADNP homeobox 2	18q23	22850	ZNF508
**Tshz**	*TSHZ1*	teashirt zinc finger homeobox 1	18q22.3	10194	TSH1
	*TSHZ2*	teashirt zinc finger homeobox 2	20q13.2	128553	TSH2, ZNF218, ZABC2, OVC10-2
	*TSHZ3*	teashirt zinc finger homeobox 3	19q12	57616	TSH3, ZNF537
**Zeb**	*ZEB1*	zinc finger E-box binding homeobox 1	10p11.22	6935	ZFHX1A, deltaEF1, TCF8, ZEB
	*ZEB2*	zinc finger E-box binding homeobox 2	2q22.3	9839	ZFHX1B, SIP1, SMADIP1
	*ZEB2P1*	zinc finger E-box binding homeobox 2 pseudogene 1	4p15.32	100033412	
**Zfhx**	*ZFHX2*	zinc finger homeobox 2	14q11.2	85446	
	*ZFHX3*	zinc finger homeobox 3	16q22.3	463	ATBT, ATBF1
	*ZFHX4*	zinc finger homeobox 4	8q21.11	79776	ZFH4
**Zhx/**	*ZHX1*	zinc fingers and homeoboxes 1	8q24.13	11244	
**Homez**	*ZHX2*	zinc fingers and homeoboxes 2	8q24.13	22882	
	*ZHX3*	zinc fingers and homeoboxes 3	20q12	23051	TIX1
	*HOMEZ*	homeobox and leucine zipper encoding	14q11.2	57594	

**Human CERS-class homeobox genes**					

**Cers**	*CERS2*	ceramide synthase 2	1p36.13-q24.1	29956	LASS2, TRH3, TMSG1
	*CERS3*	ceramide synthase 3	15q26.3	204219	LASS3
	*CERS4*	ceramide synthase 4	19p13.3	79603	LASS4, TRH1
	*CERS5*	ceramide synthase 5	12q13.12	91012	LASS5, TRH4
	*CERS6*	ceramide synthase 6	2q31	253782	LASS6

Human homeobox genes and pseudogenes, excepting the ANTP and PRD classes, including full names, chromosomal locations, Entrez Gene IDs and previous symbols. The *HOMEZ *gene is in the ZF class but encodes a protein with leucine zippers instead of zinc fingers.

### The POU homeobox class

The POU class generally encodes proteins with a POU-specific domain (named from the mammalian genes *Pit1 *(now *Pou1f1*), *OCT1 *and *OCT2 *(now *POU2F1 *and *POU2F2*), andnematode *unc-86*) N-terminal to a typical homeodomain. The POU-specific domain is a DNA-binding domain of approximately 75 amino acids; the POU-specific domain and the homeodomain are collectively known as the bipartite POU domain [61].

We have identified a total of 16 POU-class homeobox genes in the human genome (Tables 1 and 4). The genes form a distinct grouping even if the POU-specific domain is disregarded – phylogenetic analyses of homeodomains recover the POU class as a monophyletic group (Figure 3; Additional files 1, 2 and 5). There are six widely recognized gene families within the POU class (Pou1 to Pou6), and nomenclature revisions approximately 10 years ago clarified which genes belong to which gene family [62]. We have placed two additional genes (*HDX *and *POU5F2*) in the POU class on the basis of their deduced homeodomain sequences, even though one of these genes (*HDX*) does not encode a POU-specific domain. We have erected a new gene family for this gene, bringing the total number of gene families in the POU class to seven. We have also identified a total of eight POU-class pseudogenes in the human genome (Tables 1 and 4); we have named six of these (*POU5F1P2*, *POU5F1P4 *to *POU5F1P8*), and revised the nomenclature of one other (*POU5F1P3*).

∘ *HDX *[Entrez Gene ID: 139324]. This gene was previously known as *CXorf43*. The gene encodes a highly divergent atypical (68-amino-acid) homeodomain but not a POU-specific domain, and thus it is debatable whether it should be placed within the POU class. Phylogenetic analyses of homeodomains place it basally in a clade with the POU class (Figure 3; Additional files 1 and 5), or within the POU class (Additional file 2), suggesting that the HDX protein either diverged before the POU-specific domain became associated with the homeodomain or lost the POU-specific domain during evolution. Further information on this gene may allow this tentative classification to be revisited.

∘ *POU5F2 *[Entrez Gene ID: 134187]. We designate this previously unnamed gene *POU5F2 *on the basis of clear orthology to the mouse *Sprm1 *gene, which has been assigned the second member of the Pou5 gene family [63]. The symbol *POU5F2 *ensures the gene conforms with standardized nomenclature for the POU class.

∘ *POU5F1P2 *[GeneID: 100009665], *POU5F1P3 *(formerly *POUF51L*) [GeneID: 5461], *POU5F1P4 *[GeneID: 100009666], *POU5F1P5 *[GeneID: 100009667], *POU5F1P6 *[GeneID: 100009668], *POU5F1P7 *[GeneID: 100009669] and *POU5F1P8 *[GeneID: 100009670]. Prior to this study, a single retrotransposed pseudogene of the *POU5F1 *gene had been annotated and designated *POU5F1P1 *[Entrez Gene ID: 5462]. Another *POU5F1*-related sequence of unknown status had been annotated and designated *POUF5F1L *[GeneID: 5461]. We replace the gene symbol *POUF5F1L *with *POU5F1P3 *as this sequence is a retrotransposed pseudogene of *POUF51*. Our analyses of the human genome sequence identified a further six pseudogenes of *POU5F1*, which we name sequentially *POU5F1P2*, *POU5F1P4 *through to *POU5F1P8*. Each clearly aligns to the mRNA sequence of *POU5F1 *but with sequence alterations, indicating origin by retrotransposition. *POU5F1P2 *and *POU5F1P6 *have frameshift mutations in the homeobox. *POU5F1P5 *and *POU5F1P6 *have stop codons in the homeobox. *POU5F1P7 *and *POU5F1P8 *are partial integrants of *POU5F1 *mRNA excluding the homeobox – *POU5F1P7 *covers part of the 3' untranslated region and *POU5F1P8 *a short region around the start codon.

### The HNF homeobox class

The HNF class (named after the rat gene *Hnf1*) encodes proteins with a POU-like domain N-terminal to a highly atypical homeodomain. The POU-like domain, as its name indicates, is weakly similar in sequence to the POU-specific domain [64]; more importantly, it has nearly the same three-dimensional structure and mode of DNA binding as the POU-specific domain [65].

We have identified a total of three HNF-class homeobox genes in the human genome (Tables 1 and 4), consistent with previous work [66,67]. The homeodomains encoded by the human *HNF1A *and *HNF1B *genes are atypical in possessing 21 extra amino acid residues between the second and third alpha helices (Additional file 6). We place these two genes in a single gene family (Hnf1) within the HNF class, implying derivation from a single invertebrate gene. Examination of their chromosomal locations concurs with this view. *HNF1A *and *HNF1B *map to parts of the genome known to have duplicated in early vertebrate evolution, namely 12q24.31 (*HNF1A*, near *LHX5 *and on the same arm as the HOXC cluster) and 17q12 (*HNF1B*, between *LHX1 *and the HOXB cluster) (Figure 4). The use of the A and B suffixes is unfortunate, as numerals are generally used to distinguish paralogs of this age, but is retained at present due to widespread and stable use. The homeodomain encoded by the human *HMBOX1 *gene is atypical in possessing 15 extra amino acid residues between the second and third alpha helices (Additional file 6). Phylogenetic analyses confirm previous suggestions [67] that *HMBOX1 *is more distantly related to *HNF1A *and *HNF1B *(Figure 3; Additional files 1, 2 and 5). We place this gene in a separate gene family (Hmbox) within the same class. We have not found any human HNF-class pseudogenes.

### The SINE homeobox class

The SINE class (named after the *Drosophila *gene *so*: *sine oculis*) encodes proteins with a SIX domain N-terminal to a typical homeodomain. The SIX domain is a DNA-binding domain of approximately 115 amino acids; both the SIX domain and the homeodomain are required for DNA binding [68].

We have identified a total of six SINE-class homeobox genes in the human genome (Tables 1 and 4), consistent with previous work [68,69]. The genes form a distinct grouping even if the SIX domain is disregarded – phylogenetic analyses of homeodomains recover the SIX class as a monophyletic group (Figure 3; Additional files 1, 2 and 5). Phylogenetic analyses of homeodomains divide the SIX class into three gene families (Figure 3; Additional files 1, 2 and 5), consistent with previous studies [68,69]. Each gene family has two human members and dates to a single ancestral gene in the most recent common ancestor of bilaterians [68,69]. We have not found any human SINE-class pseudogenes.

### The TALE homeobox class

TALE (three amino acid loop extension) class genes are distinguished by the presence of three extra amino acids between the first and second alpha helices of the encoded homeodomain [1,2,70]. Genes belonging to the TALE class encode proteins with various domains outside of the atypical homeodomain.

We have identified a total of 20 TALE-class homeobox genes in the human genome (Tables 1 and 4). The genes form a distinct grouping in phylogenetic analyses even when the three extra homeodomain residues are excluded from the sequence alignment (Figure 3; Additional file 5). Bürglin [2] has given the TALE group the rank of 'superclass' and distinguished between several 'classes' by the presence of distinct domains outside of the homeodomain. These are the IRX domain, MKX domains, the MEIS domain, the PBC domain and TGIF domains [2,71-73]. Along with some others [4,7,24], we have given the TALE group the rank of 'class' containing several 'gene families'; this maintains consistent terminology throughout the present paper. Phylogenetic analyses of homeodomains divide the TALE class into six gene families (Figure 3; Additional files 1, 2 and 5), including an Mkx family containing the recently described *MKX *gene, which is distinguished from Irx-family genes phylogenetically and by absence of an IRX domain [73,74]. It should be noted that the established name of the Pknox gene family does not indicate orthology with Knox-family genes of plants. We have also identified a total of 10 TALE-class pseudogenes in the human genome (Tables 1 and 4); we have named six of these (*IRX4P1*, *TGIF1P1 *and *TGIF2P1 *to *TGIF2P4*), and revised the nomenclature of two others (*IRX1P1 *and *PBX2P1*).

∘ *IRX1P1 *[Entrez Gene ID: 646390]. This sequence was previously known as *IRXA1*; we rename it *IRX1P1 *because it is clearly a retrotransposed pseudogene of *IRX1 *and not a functional gene. The *IRX1P1 *sequence aligns to the mRNA of *IRX1 *but has a frameshift mutation and two stop codons in the homeobox.

∘ *IRX4P1 *[Entrez Gene ID: 100009671]. We designate this previously unannotated sequence *IRX4P1 *because it is clearly a retrotransposed pseudogene of *IRX4*. The *IRX4P1 *sequence is a partial integrant derived from a region of the *IRX4 *mRNA around the stop codon; it lacks the homeobox.

∘ *PBX2P1 *[Entrez Gene ID: 5088]. This sequence was previously known as *PBXP1*; we rename it *PBX2P1 *because it is clearly a retrotransposed pseudogene of *PBX2*. The former name of *PBXP1 *did not indicate its transcript of origin. The *PBX2P1 *sequence aligns to the mRNA of *PBX2 *but has a frameshift mutation in the coding region.

∘ *TGIF1P1 *[Entrez Gene ID: 126052]. We designate this previously unannotated sequence *TGIF1P1 *because it is clearly a retrotransposed pseudogene of *TGIF1*. The locus has many sequence alterations when compared to *TGIF1 *mRNA, including a 48 nucleotide insertion within the homeobox.

∘ *TGIF2P1 *[GeneID: 126826], *TGIF2P2 *[GeneID: 100009674], *TGIF2P3 *[GeneID: 100009672] and *TGIF2P4 *[GeneID: 100009673]. These four sequences were unannotated prior to this study. We designate them *TGIF2P1 *to *TGIF2P4 *because they are clearly pseudogenes of *TGIF2*. Each aligns to the mRNA sequence of *TGIF2 *but with sequence alterations, indicating origin by retrotransposition. *TGIF2P1 *has many sequence alterations, including a frameshift mutation in the homeobox. *TGIF2P2 *and *TGIF2P3 *are very similar neighboring loci that must have originated by tandem duplication of a retrotransposed *TGIF2 *mRNA; neither includes the homeobox. *TGIF2P4 *is a short partial integrant derived from part of the 3' untranslated region of *TGIF2 *mRNA.

### The CUT homeobox class

The CUT class (named after the *Drosophila *gene *cut*) generally encodes proteins with one or more CUT domains N-terminal to a typical homeodomain. The CUT domain is a DNA-binding domain of approximately 75 amino acids [75]. There are three widely recognized gene families within the CUT class in humans (Onecut, Cux, Satb; [76]). A fourth gene family (Cmp), lacking a CUT domain but sharing a CMP domain with the Satb gene family, is absent from vertebrates. Bürglin and Cassata [76] have proposed that the vertebrate Satb gene family evolved from the invertebrate Cmp gene family.

We have identified a total of seven CUT-class homeobox genes in the human genome (Tables 1 and 4). Although grouped together by presence of CUT domains, the homeodomains of the Onecut, Cux and Satb gene families are quite divergent and do not always form a monophyletic group in phylogenetic analyses (Additional files 2 and 5). Topologies that separate the gene families are also only weakly supported, so it is most parsimonious to assume that the class is actually monophyletic but the constituent genes underwent rapid sequence divergence following their initial duplications. We have revised the nomenclature of two CUT-class genes (*CUX1 *and *CUX2*). We have also identified a total of three CUT-class pseudogenes in the human genome (Tables 1 and 4); we have named all of these (*CUX2P1*, *CUX2P2 *and *SATB1P1*).

∘ *CUX1 *[Entrez Gene ID: 1523] and *CUX2 *[Entrez Gene ID: 23316]. These genes were previously known as *CUTL1 *and *CUTL2 *respectively. We rename them *CUX1 *and *CUX2 *in accordance with homeobox gene nomenclature convention.

∘ *CUX2P1 *and *CUX2P2*. These sequences were unannotated prior to this study. We designate them *CUX2P1 *and *CUX2P2 *because they are clearly retrotransposed pseudogenes of *CUX2*. Both are short partial integrants derived from *CUX2 *mRNA, excluding the homeobox – *CUX2P1 *covers part of the coding region at the 5' end and *CUX2P2 *part of the 3' untranslated region.

∘ *SATB1P1 *[Entrez Gene ID: 100033410]. We designate this previously unannotated sequence *SATB1P1 *because it is clearly a retrotransposed pseudogene of *SATB1*. *SATB1P1 *is a short partial integrant derived from part of the 3' untranslated region of *SATB1 *mRNA; it does not encompass the homeobox.

### The PROS homeobox class

The PROS class (named after the *Drosophila *gene *pros*) encodes proteins with a PROS domain C-terminal to an atypical homeodomain. The PROS domain is a DNA-binding domain of approximately 100 amino acids [77]. PROS-class genes encode a highly divergent homeodomain with three extra amino acids. These additional residues are inserted at a different position compared to the TALE class, being between the second and third alpha helices (Additional file 6).

We have identified a total of two PROS-class homeobox genes in the human genome (Tables 1 and 4), which we have placed in a single gene family (Prox). The highly divergent homeodomain sequence and unusual structural features provide justification for PROS being a separate gene class, despite the small number of genes. In phylogenetic analyses, PROS-class homeodomains are situated on a long branch, very distant from other classes (Figure 3; Additional files 1, 2 and 5). The human *PROX1 *gene is well characterized; we have identified and named its paralog, *PROX2*. We have not found any human PROS-class pseudogenes.

∘ *PROX2 *[Entrez Gene ID: 283571]. We designate this previously unannotated gene *PROX2 *on the basis of clear orthology to the mouse *Prox2 *gene, inferred from sequence identity and synteny. The homeobox of human *PROX2 *has two introns and unusually the splice sites of the first (5') intron (AT-AA) do not follow the GT-AG donor-acceptor rule. This has also been noted for mouse *Prox2 *[78].

### The ZF homeobox class

The ZF (zinc finger) class generally encodes proteins with zinc finger motifs, in addition to one or more homeodomains. As noted earlier, phylogenetic analyses of homeodomains does not recover the ZF class as a monophyletic group (Figure 3; Additional files 1, 2 and 5). We recognize that this suggests that zinc finger motifs and homeodomains may have been brought together on three separate occasions in evolution; nonetheless, it is convenient and informative to group these into a single class. Inclusion of the *HOMEZ *gene in the ZF class may be surprising, as this gene does not encode zinc fingers. However, as previously noted [79] and reproduced in our phylogenetic analyses (Figure 3; Additional files 1, 2 and 5), the multiple homeodomain sequences of this gene are clearly related to those encoded by the *ZHX1*, *ZHX2 *and *ZHX3 *genes.

We have identified a total of 14 ZF-class homeobox genes in the human genome (Tables 1 and 4), which we have placed in five gene families (Adnp, Tshz, Zeb, Zfhx and Zhx/Homez). We have also identified one ZF-class pseudogenes in the human genome (Tables 1 and 4). We have revised the nomenclature of five of these loci (*ADNP2*, *ZEB1*, *ZEB2*, *ZEB2P1 *and *ZFHX3*).

∘ *ADNP2 *[Entrez Gene ID 22850]. This gene was previously known as *ZNF508*; we rename it *ADNP2 *to reflect its paralogous relationship to *ADNP*.

∘ *ZEB1 *[Entrez Gene ID: 6935] and *ZEB2 *[Entrez Gene ID: 9839]. These genes were previously known as *ZFHX1A *and *ZFHX1B *respectively. We rename them *ZEB1 *and *ZEB2 *to distinguish them from genes belonging to the distantly related Zfhx gene family.

∘ *ZEB2P1 *[Entrez Gene ID: 100033412]. This retrotransposed pseudogene of *ZEB2 *has been described previously [80]. Our new nomenclature (*ZEB2P1*) reflects the origin of this locus.

∘ *ZFHX3 *[Entrez Gene ID: 463]. This gene was previously known as *ATBF1*; we rename it *ZFHX3 *to reflect its close relationship to *ZFHX2 *and *ZFHX4*; indeed *ZFHX3 *was a synonym for this gene.

### The CERS homeobox class

The highly unusual CERS (ceramide synthase) class, also known as the LASS (longevity assurance) class, comprises a single gene family that is highly conserved amongst eukaryotes and includes the yeast gene and original member *LAG1*. There are six CERS-class genes in the human genome (*CERS1 *to *CERS6*) and five of these (*CERS2 *to *CERS6*) encode proteins with a homeodomain sequence [81,82]. These are, however, extremely divergent from the homeodomains of other gene classes. Secondary structure prediction analyses suggest these sequences have the potential to encode three alpha helices in the appropriate positions (data not shown). The most surprising characteristic of these genes is that biochemical studies predict them to encode transmembrane proteins, with the homeodomain on the cytosolic side of the endoplasmic reticulum membrane, and hence they could not act as DNA-binding proteins or transcription factors [81,82]. It is possible that an ancestor of these genes gained a homeobox through exon shuffling, or alternatively this could represent convergent evolution. We include only *CERS2 *to *CERS6 *in our comprehensive compilation of human homeobox genes, as *CERS1 *lacks a homeobox motif.

### Chromosomal distribution of human homeobox genes

The chromosomal locations of genes can give clues to evolutionary ancestry, including patterns of gene duplication, and the possible existence of gene clusters. In Figure 4, we show the chromosomal locations of all human homeobox genes. We do not include probable pseudogenes on these ideograms, because most of these have originated by reverse transcription of mRNA and secondary integration into the genome, and hence give no insight into ancestral locations of genes. The highly repetitive *DUX1 *to *DUX5 *sequences are also not shown, as these have undergone secondary amplification and are also most likely non-functional (see above).

The first observation is that there are homeobox genes on every human chromosome. Even the two sex chromosomes harbor homeobox genes, with *SHOX *(*short stature homeobox*) in the PAR1 pseudoautosomal region at the tip of the short arms of X and Y being the best known. Haploinsufficiency of *SHOX *is implicated in the short stature phenotype of Turner syndrome patients who lack one copy of the X chromosome [83]. There are also nine other homeobox genes in non-pseudoautosomal regions of the X chromosome, including three tandemly-arranged members of the Rhox gene family, collectively homologous to the multiple Rhox (reproductive homeobox) genes of mouse. Only one of the homeobox genes on the X chromosome, the TALE-class gene *TGIF2LX*, has a distinct homolog on the Y chromosome, called *TGIF2LY*. These genes map to the largest homology block shared by the unique regions of the X and Y chromosomes, spanning 3.5 Mb. It has been proposed that the ancestor of these two genes arose by retrotransposition of *TGIF2 *mRNA [84].

The autosomes with the lowest number of homeobox genes are chromosomes 21 (with just *PKNOX1*) and 22 (with *GSC2 *and *ISX*). Examination of the remaining autosomes reveals that homeobox genes are quite dispersed with some interesting regional accumulations. The best known examples of close linkage between homeobox genes are the four Hox clusters on human chromosomes 2, 7, 12 and 17, comprising 9, 11, 9 and 10 genes respectively; each of these is shown as just a single line on each ideogram for simplicity (Figure 4). These should not be considered in isolation, however, because many other ANTP-class genes map in the vicinity of the Hox clusters [26,27]. These include genes very tightly linked to the Hox clusters, notably the Evx-family genes (on chromosomes 2 and 7), Dlx-family genes (on chromosomes 2 and 17), and Meox-family genes (on chromosomes 2 and 17).

There are other concentrations of ANTP-class genes away from the Hox clusters. These are the ParaHox cluster (*GSX1*, *PDX1*, *CDX2*) on chromosome 13, and four sets of NKL-subclass genes on 2p/8p (split), 4p, 5q and 10q, hypothesized to be derived from an ancestral array by duplication [26,33]. The accumulation on the distal half of the long arm of chromosome 10 is particularly striking, comprising eleven ANTP-class genes from 10 gene families. This is not a tight gene cluster, but it is compatible with ancestry by extensive tandem gene duplication followed by dispersal. Discounting the rather aberrant case of the Hox clusters, this region of the long arm of chromosome 10 is the most homeobox-rich region of the human genome.

There are additional groupings of homeobox genes outside the ANTP class. These include two TALE-class Irx clusters on chromosomes 5 and 16 homologous to the described mouse Irx clusters [19], and a set of PRD-class genes on chromosome 19 proposed to be derived from the *CRX *homeobox gene by duplication and rapid divergence [21]. Perhaps the most interesting case, however, is found on the tip of the long arm of chromosome 9, where there is a concentration of homeobox genes from disparate gene classes. Four LIM-class genes, one ANTP-class gene, one PRD-class gene and one TALE-class gene are found in this location. Although dispersed over a large region, and not forming a tight gene cluster, the linkages are nonetheless intriguing. It is possible that these linkages reflect ancestry from the very ancient gene duplications that must have generated the distinctive homeobox gene classes found within animal genomes.

## Conclusion

We identified 300 homeobox loci in the euchromatic regions of the human genome, and divide these into 235 probable functional genes and 65 probable pseudogenes. Not all of these loci possess a homeobox because for completeness we include all sequences derived from homeobox-containing genes. The number of homeobox sequences is also different from the number of loci because several genes contain multiple homeobox motifs. The figures exclude the repetitive *DUX1 *to *DUX5 *homeobox sequences of which we identified 35 probable pseudogenes, with many more expected in heterochromatic regions.

New or revised nomenclature is proposed for approximately 70 of the 300 homeobox loci in order to clarify orthologous relationships between human and mouse, to indicate evolutionary relationships within a gene family, to distinguish genes from pseudogenes, and to indicate pseudogene origins. The loci are also classified into a simple hierarchical scheme, comprising 102 gene families within eleven gene classes. The classification scheme proposed may be widely applicable to homeobox genes from other animals.

The 235 probable functional homeobox genes map to every human chromosome with some interesting regional concentrations of genes. These include a large number of ANTP-class genes on the distal end of the long arm of chromosome 10, and a combination of LIM-, ANTP-, PRD- and TALE-class genes on the distal end of the long arm of chromosome 9. These associations may be remnants of common ancestry early in animal evolution.

## Methods

The finished human genome sequence (build 35.1) was subjected to a series of tBLASTn searches [85,86] using known homeodomain sequences from the ANTP, PRD, LIM, POU, HNF, SINE, TALE, CUT, PROS and ZF classes. No arbitrary E-value cut-off was selected, but instead each list of hits was analyzed manually until true homeodomain sequences ceased to be detected. Definition of a homeodomain used a combination of CD-search for conserved protein domains implemented through BLASTp [85,86] and secondary structure prediction by JPred implemented through the Barton Group, University of Dundee [87]. Each time a new or divergent homeodomain match was found, the tBLASTn process was repeated. Six very divergent gene families were undetected by this method but found by text searching: Hopx, Adnp, Tshz, Zeb, Zhx/Homez and Cers. To ensure that every pseudogene was detected, including truncated or decayed versions lacking the homeobox, the full mRNA sequence of each gene was deduced and used in a BLASTn search of the human genome sequence [85,86]. Pseudogenes were recognized as those genomic regions with similarity to non-repetitive DNA sequences of the parent gene, even if aligning to only part of the locus. Pseudogenes undergo mutational decay and would eventually become unrecognizable, but in practice ambiguous cases were not encountered. Exon-intron structures of novel loci were deduced by comparison between genomic sequence and cDNA, EST or retrotransposed pseudogene sequences, as previously described [21]. Several unnamed human loci were identified as probable orthologs of known mouse genes; orthology was deduced by a combination of homeodomain sequence similarity and synteny, examined through the mouse genome sequence (build 34.1) and the Ensembl Genome Browser [88].

Phylogenetic analyses were performed with homeodomain sequences, after each had been edited to an alignment of 60 amino acids (Additional file 7), using the maximum likelihood [89] and neighbor-joining [90] methods. Maximum likelihood trees were constructed using PhyML [91], with a JTT model of amino acid substitution, four categories of between-site rate heterogeneity, a gamma distribution parameter estimated from the data and 500 bootstrap resamplings. Neighbor-joining trees were constructed using PHYLIP (.)[92], with a JTT model of amino acid substitution and 1000 bootstrap resamplings. For defining human gene families, all *Drosophila *homeodomains were first combined with all human homeodomains in maximum likelihood and neighbor-joining analyses to enable divergent *Drosophila *genes to be identified and removed. These include genes lost from human, as well genes known to have undergone unusually rapid evolution in *Drosophila*. For the Hox3 family the rapidly evolving *Drosophila *genes *bcd*, *zen *and *zen2 *were then replaced by an ortholog from centipede (*Sm Hox3b*), and for the Nk4 family the rapid evolving *Drosophila *gene *tin *was replaced by an ortholog from annelid (*Pd NK4*). In addition, six genes from other protostome or cnidarian genomes were added to represent gene families known to be missing from *Drosophila *(Pdx family: *Ps Xlox*; Alx family: *Nv CART1*; Dmbx family: *Hv manacle*; Pou1 family: *Nv POU1*; Hnf1 family: *Nv HNF*; Pknox family: *Am Prep*). Only 100 bootstrap resamplings were performed on this dataset because of its large size (354 homeodomains). Trees were displayed using TreeExplorer [93]. Genes encoding partial homeodomains, and probable pseudogenes, were not included in the phylogenetic analyses. With short alignments, phylogenetic trees can only be used as guides to relationships, not absolute indicators of evolutionary history, and the trees presented in this paper should be interpreted in this light.

## Authors' contributions

PWHH designed the study and contributed to gene identification and to gene nomenclature revisions. HAFB carried out database searches, annotations and phylogenetic analyses and contributed to gene nomenclature revisions. PWHH and HAFB drafted the manuscript. EB contributed to gene nomenclature revisions, discussed these with the research community and databases, and implemented the agreed changes.

All authors edited and approved the final manuscript.

## Supplementary Material

Additional file 1 **Maximum likelihood phylogenetic tree of all human plus selected protostome and cnidarian homeodomains for identification of gene families**. Arbitrarily rooted phylogenetic tree of all human plus selected protostome and cnidarian homeodomains constructed using the maximum likelihood (ML) method. Bootstrap values supporting gene family designations are shown. Homeodomain sequences derived from pseudogenes are excluded. This ML tree should be compared with the neighbor-joining (NJ) tree shown in Additional file 2. The dataset used for both ML and NJ analyses includes all human homeodomains, most *Drosophila melanogaster *homeodomains, plus selected additional homeodomains from other protostomes or cnidarians when the gene family is divergent or absent in *Drosophila*. Divergent *Drosophila *genes that do not group with human genes were identified by construction of a preliminary, non-bootstrapped ML and NJ trees, and subsequently removed from the dataset. These include genes lost from human, as well genes known to have undergone unusually rapid evolution in *Drosophila*. For the Hox3 family the rapidly evolving *Drosophila *genes *bcd*, *zen *and *zen2 *were replaced with *Sm Hox3b*, and for the Nk4 family the rapid evolving *Drosophila *gene *tin *was replaced with *Pd NK4*. In addition, six genes from other protostome or cnidarian genomes were added to represent gene families known to be missing from *Drosophila *(Pdx family: *Ps Xlox*; Alx family: *Nv CART1*; Dmbx family: *Hv manacle*; Pou1 family: *Nv POU1*; Hnf1 family: *Nv HNF*; Pknox family: *Am Prep*). Species abbreviations: *Am*, *Apis mellifera *(honeybee); *Dm*, *Drosophila melanogaster *(fruitfly); *Hv*, *Hydra vulgaris *(hydrozoan); *Nv*, *Nematostella vectensis *(starlet sea anemone); *Pd*, *Platynereis dumerilii *(annelid worm); *Ps*, *Phascolion strombus *(sipunculan worm); *Sm*, *Strigamia maritima *(centipede). ML performed more poorly than NJ in recovering several well known gene families, notably Hox4, Hox5, Nk4 and Alx. In contrast, ML did recover *PROP1 *and *CG32532 *as a true gene family; NJ did not. The invertebrate gene does not always lie as a strict outgroup to all human genes in a family; this effect is expected when using a short alignment. Instead, distinct grouping of invertebrate and human genes is taken as evidence of ancestry from a single gene. A few ambiguous cases were encountered, notably divergence of *Drosophila H2.0 *in the proposed Hlx gene family, and resolution within the Pax4/6 gene family, which is recovered as two families in NJ but one in ML. As explained in the text, several human gene families contain 'orphan' genes without invertebrate orthologs; these are Barx, Nanog, Noto, Vax, Ventx, Argfx, Dprx, Dux, Esx, Hesx, Hopx, Isx, Leutx, Mix, Nobox, Rhox, Sebox, Tprx, Hdx, Pou5, Hmbox, Satb, Adnp and Zhx/Homez. Zeb and Mkx would be placed in this category based on our ML and NJ trees, although other data suggest that *Drosophila zfh1 *and *CG11617 *respectively may be the protostome orthologs [73,94]. Tshz is only an apparent orphan family; the clear *Drosophila *ortholog simply lacks the homeobox [95,96]. Phylogenetic analysis is just one source of evidence for allocation of genes to gene families and identification of boundaries between gene families; complementary criteria used are synteny between species and paralogy within the human genome. Our ML and NJ trees should not be used to allocate gene families to gene classes, because other diagnostic characters such as insertions within the homeodomain, key amino acid residues, and several motifs outside of the homeodomain are excluded from the analysis. Indeed, artefactual mixing of the TALE and SINE classes occurs in both ML and NJ trees. Click here for file

Additional file 2 **Neighbor-joining phylogenetic tree of all human plus selected protostome and cnidarian homeodomains for identification of gene families**. Arbitrarily rooted phylogenetic tree of all human plus selected protostome and cnidarian homeodomains constructed using the neighbor-joining (NJ) method. Bootstrap values supporting gene family designations are shown. Homeodomain sequences derived from pseudogenes are excluded. Comparison of NJ and ML trees, and description of the dataset used, is given in the legend to Additional file 1. Several artefactual mixing of classes occurs in this NJ tree, notably splitting of the CUT class, mixing of the TALE and SINE classes and aberrant placement of *HOPX*. Click here for file

Additional file 3 **Neighbor-joining phylogenetic tree of human ANTP-class homeodomains, for comparison to maximum likelihood tree**. Arbitrarily rooted phylogenetic tree of human ANTP-class homeodomains constructed using the neighbor-joining method. Bootstrap values supporting internal nodes with over 70% are shown. Homeodomain sequences derived from pseudogenes are excluded. The proposed division between the HOXL and NKL subclasses is indicated. The position of *EN1 *and *EN2 *is unstable; this tree places them close to the base of the HOXL/NKL divergence, whereas maximum likelihood analysis of the same dataset places them firmly in the NKL subclass (Figure 1). Interrelationships of genes in the Nk2.2 and Nk4 families are also unstable (in this tree and Figure 1 respectively); in these cases synteny within and between genomes clearly resolves gene families. Detailed relationships between different gene families should not be inferred from this tree. Click here for file

Additional file 4 **Neighbor-joining phylogenetic tree of human PRD-class homeodomains, for comparison to maximum likelihood tree**. Arbitrarily rooted phylogenetic tree of human PRD-class homeodomains constructed using the neighbor-joining method. Bootstrap values supporting internal nodes with over 70% are shown. Homeodomain sequences derived from pseudogenes are excluded, as are the partial homeodomains of PAX2, PAX5 and PAX8, and the HOPX homeodomain because its extremely divergent sequence destabilizes the overall tree topology. Roman numeral suffixes are used to distinguish multiple homeodomains encoded by a single Dux-family gene. Detailed relationships between different gene families should not be inferred from this tree. Click here for file

Additional file 5 **Neighbor-joining phylogenetic tree of human homeodomains excluding ANTP and PRD classes, for comparison to maximum likelihood tree**. Arbitrarily rooted phylogenetic tree of human homeodomains excluding the ANTP and PRD classes constructed using the neighbor-joining method. Bootstrap values supporting internal nodes with over 70% are shown. Homeodomain sequences derived from pseudogenes are excluded. Roman numeral suffixes are used to distinguish multiple homeodomains encoded by a single gene. Classes and/or families are color coded as shown in the key. The LIM and ZF classes are not recovered as two distinct monophyletic groups, a result also found by maximum likelihood analysis (Figure 3). The multiple homeodomains of Zfhx-family proteins and Zhx/Homez-family proteins are also dispersed in the tree, presumably artefactually. Monophyly of the CUT class is not recovered in this tree, but is by maximum likelihood analysis (Figure 3). Detailed relationships between different gene families should not be inferred from this tree. Click here for file

Additional file 6 **Multiple sequence alignment of all human plus selected protostome and cnidarian homeodomains**. The consensus homeodomain sequence (shown several times for reference) was derived from a compilation of 247 human homeodomain sequences. The three horizontal lines indicate the positions of the three alpha-helices. The numbering scheme refers to amino acid position in the canonical 60-amino-acid homeodomain; insertions relative to this sequence are shown when present. Black shaded resides are invariant between all human homeodomains within each class (or family in the case of the ZF homeodomains). Sequence accession numbers are shown. For each gene family designation, maximum likelihood and neighbor-joining bootstrap support values are indicated (see Additional files 1 and 2). These values are not shown if the gene family does not form a monophyletic group in phylogenetic analyses (in which case n/a is written) or if an invertebrate homolog could not be found. Click here for file

Additional file 7 **Phylogenetic input file**. All human and invertebrate homeodomains used in phylogenetic analyses are shown, after alignment and removal of insertions to give a uniform 60-amino-acid alignment. Click here for file
