# Supplementary material for: Classification and nomenclature of all human homeobox genes
Source: BMC Biol. 2007 Oct 26;5:47. doi: 10.1186/1741-7007-5-47 (PMC2211742; doi:10.1186/1741-7007-5-47)

Additional file 1

The dataset used for both ML and NJ analyses includes all human homeodomains, most Drosophila melanogaster homeodomains, plus selected additional homeodomains from other protostomes or cnidarians when the gene family is divergent or absent in Drosophila. Divergent Drosophila genes that do not group with human genes were identified by construction of a preliminary, nonbootstrapped ML and NJ trees, and subsequently removed from the dataset. These include genes lost from human, as well genes known to have undergone unusually rapid evolution in Drosophila. For the Hox3 family the rapidly evolving Drosophila genes bcd, zen and zen2 were replaced with Sm Hox3b, and for the Nk4 family the rapid evolving Drosophila gene tin was replaced with Pd NK4. In addition, six genes from other protostome or cnidarian genomes were added to represent gene families known to be missing from Drosophila (Pdx family: Ps Xlox; Alx family: Nv CART1; Dmbx family: Hv manacle; Pou1 family: Nv POU1; Hnf1 family: Nv HNF; Pknox family: Am Prep). Species abbreviations: Am, Apis mellifera (honeybee); Dm, Drosophila melanogaster (fruitfly); Hv, Hydra vulgaris (hydrozoan); Nv, Nematostella vectensis (starlet sea anemone); Pd, Platynereis dumerilii (annelid worm); Ps, Phascolion strombus (sipunculan worm); Sm, Strigamia maritima (centipede).

ML performed more poorly than NJ in recovering several well known gene families, notably Hox4, Hox5, Nk4 and Alx. In contrast, ML did recover PROP1 and CG32532 as a true gene family; NJ did not. The invertebrate gene does not always lie as a strict outgroup to all human genes in a family; this effect is expected when using a short alignment. Instead, distinct grouping of invertebrate and human genes is taken as evidence of ancestry from a single gene. A few ambiguous cases were encountered, notably divergence of Drosophila H2.0 in the proposed Hlx gene family, and resolution within the Pax4/6 gene family, which is recovered as two families in NJ but one in ML. As explained in the text, several human gene families contain 'orphan' genes without invertebrate orthologs; these are Barx, Nanog, Noto, Vax, Ventx, Argfx, Dprx, Dux, Esx, Hesx, Hopx, Isx, Leutx, Mix, Nobox, Rhox, Sebox, Tprx, Hdx, Pou5, Hmbox, Satb, Adnp and Zhx/Homez. Zeb and Mkx would be placed in this category based on our ML and NJ trees, although other data suggest that Drosophila zfh1 and CG11617 respectively may be the protostome orthologs [73,94]. Tshz is only an apparent orphan family; the clear Drosophila ortholog simply lacks the homeobox [95,96]. Phylogenetic analysis is just one source of evidence for allocation of genes to gene families and identification of boundaries between gene families; complementary criteria used are synteny between species and paralogy within the human genome. Our ML and NJ trees should not be used to allocate gene families to gene classes, because other diagnostic characters such as insertions within the homeodomain, key amino acid residues, and several motifs outside of the homeodomain are excluded from the analysis. Indeed, artefactual mixing of the TALE and SINE classes occurs in both ML and NJ trees.

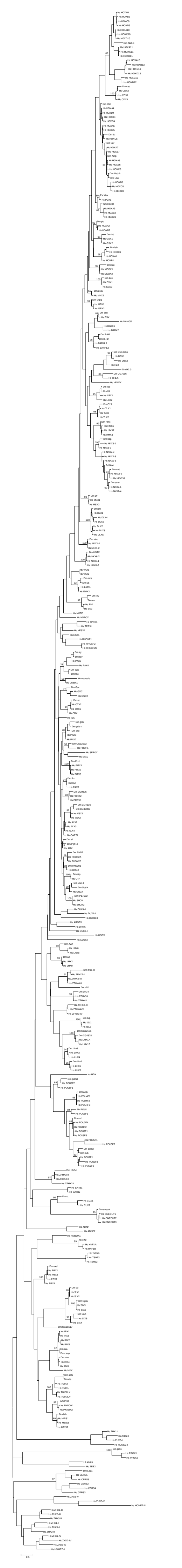

Supplement: Additional file 1 — Maximum likelihood phylogenetic tree of all human plus selected protostome and cnidarian homeodomains for identification of gene families. Arbitrarily rooted phylogenetic tree of all human plus selected protostome and cnidarian homeodomains constructed using the maximum likelihood (ML) method. Bootstrap values supporting gene family designations are shown. Homeodomain sequences derived from pseudogenes are excluded. This ML tree should be compared with the neighbor-joining (NJ) tree shown in Additional file 2. The dataset used for both ML and NJ analyses includes all human homeodomains, most Drosophila melanogaster homeodomains, plus selected additional homeodomains from other protostomes or cnidarians when the gene family is divergent or absent in Drosophila. Divergent Drosophila genes that do not group with human genes were identified by construction of a preliminary, non-bootstrapped ML and NJ trees, and subsequently removed from the dataset. These include genes lost from human, as well genes known to have undergone unusually rapid evolution in Drosophila. For the Hox3 family the rapidly evolving Drosophila genes bcd, zen and zen2 were replaced with Sm Hox3b, and for the Nk4 family the rapid evolving Drosophila gene tin was replaced with Pd NK4. In addition, six genes from other protostome or cnidarian genomes were added to represent gene families known to be missing from Drosophila (Pdx family: Ps Xlox; Alx family: Nv CART1; Dmbx family: Hv manacle; Pou1 family: Nv POU1; Hnf1 family: Nv HNF; Pknox family: Am Prep). Species abbreviations: Am, Apis mellifera (honeybee); Dm, Drosophila melanogaster (fruitfly); Hv, Hydra vulgaris (hydrozoan); Nv, Nematostella vectensis (starlet sea anemone); Pd, Platynereis dumerilii (annelid worm); Ps, Phascolion strombus (sipunculan worm); Sm, Strigamia maritima (centipede). ML performed more poorly than NJ in recovering several well known gene families, notably Hox4, Hox5, Nk4 and Alx. In contrast, ML did recover PROP1 and CG3253 [file 1741-7007-5-47-S1.pdf]
