# Supplementary figures and images for: Classification and nomenclature of all human homeobox genes
Source: BMC Biol. 2007 Oct 26;5:47. doi: 10.1186/1741-7007-5-47 (PMC2211742; doi:10.1186/1741-7007-5-47)

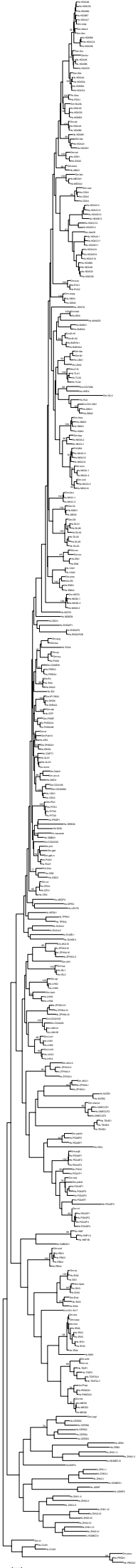

Supplement: Additional file 2 — Neighbor-joining phylogenetic tree of all human plus selected protostome and cnidarian homeodomains for identification of gene families. Arbitrarily rooted phylogenetic tree of all human plus selected protostome and cnidarian homeodomains constructed using the neighbor-joining (NJ) method. Bootstrap values supporting gene family designations are shown. Homeodomain sequences derived from pseudogenes are excluded. Comparison of NJ and ML trees, and description of the dataset used, is given in the legend to Additional file 1. Several artefactual mixing of classes occurs in this NJ tree, notably splitting of the CUT class, mixing of the TALE and SINE classes and aberrant placement of HOPX. [file 1741-7007-5-47-S2.pdf]

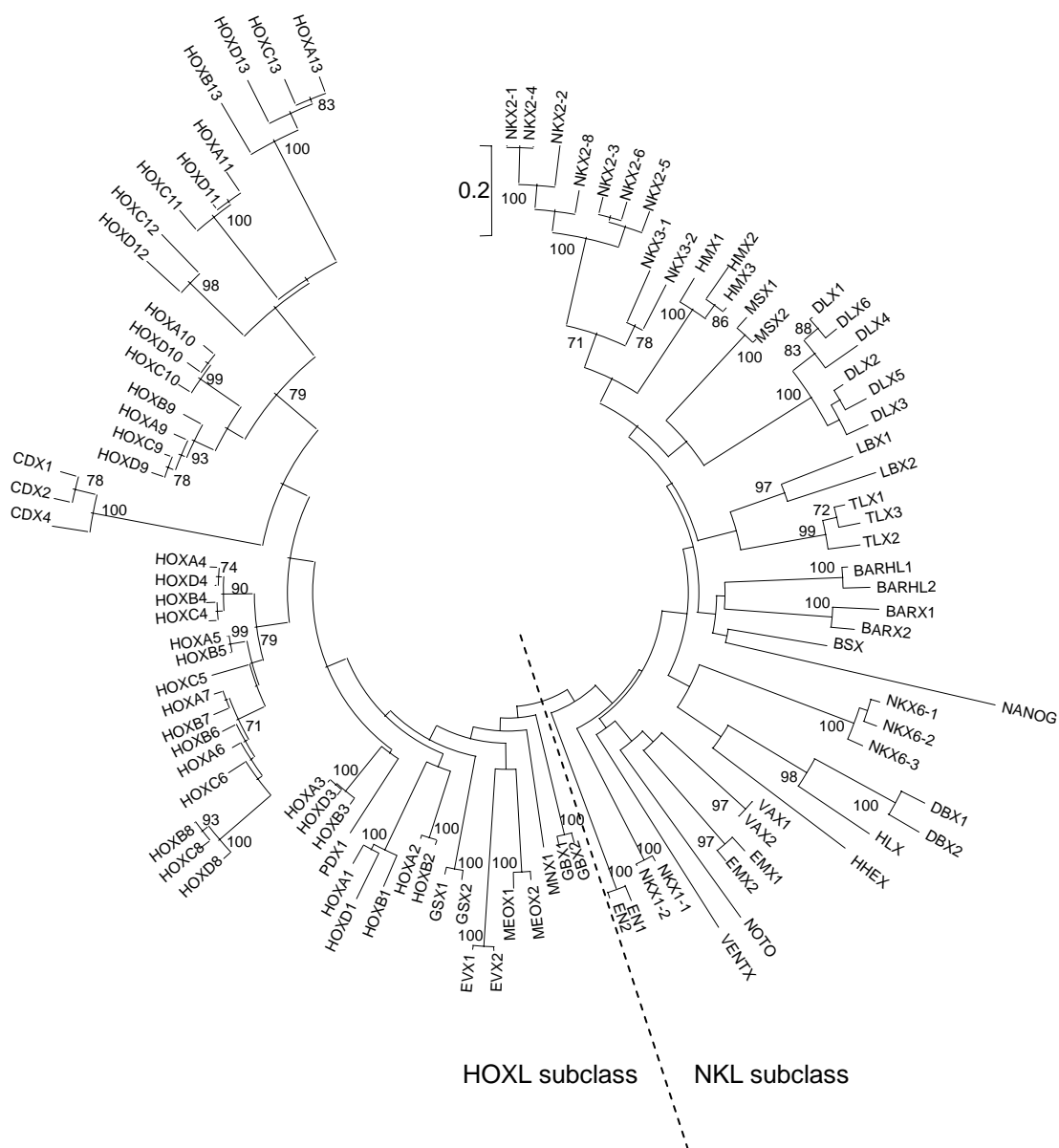

Supplement: Additional file 3 — Neighbor-joining phylogenetic tree of human ANTP-class homeodomains, for comparison to maximum likelihood tree. Arbitrarily rooted phylogenetic tree of human ANTP-class homeodomains constructed using the neighbor-joining method. Bootstrap values supporting internal nodes with over 70% are shown. Homeodomain sequences derived from pseudogenes are excluded. The proposed division between the HOXL and NKL subclasses is indicated. The position of EN1 and EN2 is unstable; this tree places them close to the base of the HOXL/NKL divergence, whereas maximum likelihood analysis of the same dataset places them firmly in the NKL subclass (Figure 1). Interrelationships of genes in the Nk2.2 and Nk4 families are also unstable (in this tree and Figure 1 respectively); in these cases synteny within and between genomes clearly resolves gene families. Detailed relationships between different gene families should not be inferred from this tree. [file 1741-7007-5-47-S3.pdf]

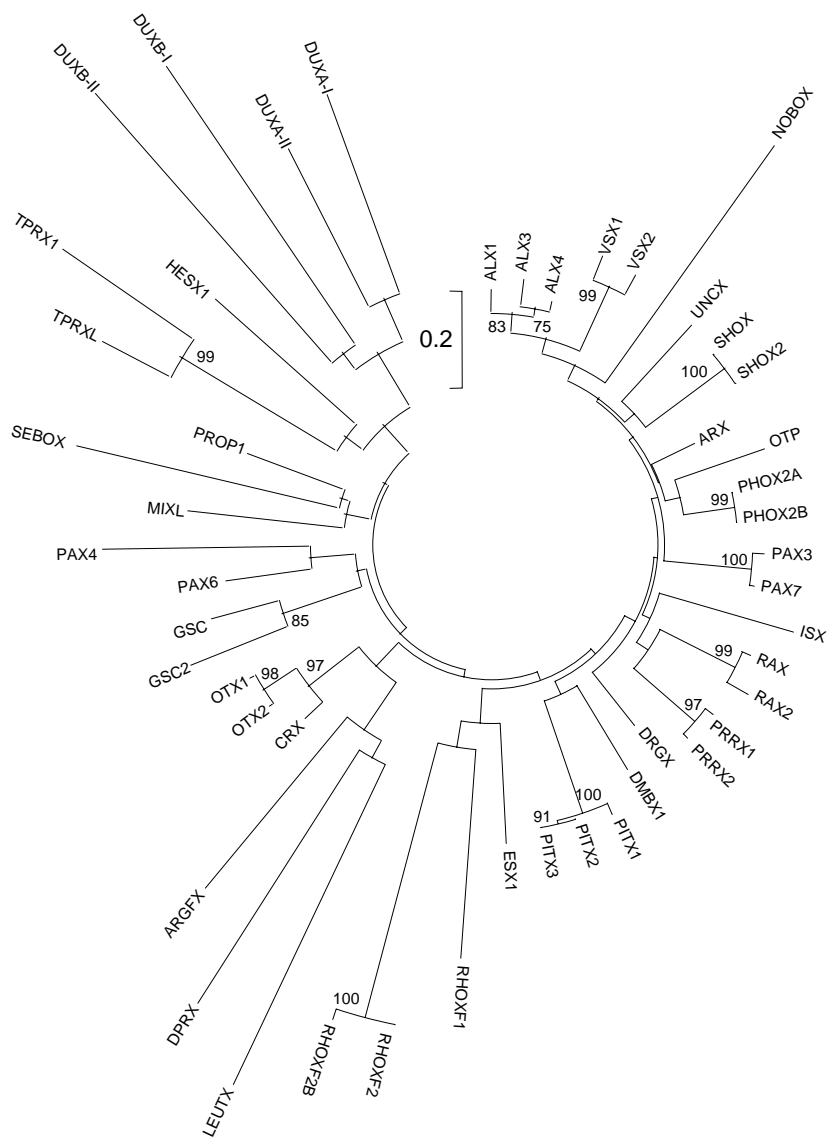

Supplement: Additional file 4 — Neighbor-joining phylogenetic tree of human PRD-class homeodomains, for comparison to maximum likelihood tree. Arbitrarily rooted phylogenetic tree of human PRD-class homeodomains constructed using the neighbor-joining method. Bootstrap values supporting internal nodes with over 70% are shown. Homeodomain sequences derived from pseudogenes are excluded, as are the partial homeodomains of PAX2, PAX5 and PAX8, and the HOPX homeodomain because its extremely divergent sequence destabilizes the overall tree topology. Roman numeral suffixes are used to distinguish multiple homeodomains encoded by a single Dux-family gene. Detailed relationships between different gene families should not be inferred from this tree. [file 1741-7007-5-47-S4.pdf]
