# Supplementary material for: Classification and nomenclature of all human homeobox genes
Source: BMC Biol. 2007 Oct 26;5:47. doi: 10.1186/1741-7007-5-47 (PMC2211742; doi:10.1186/1741-7007-5-47)
Supplement: Additional file 6 — Multiple sequence alignment of all human plus selected protostome and cnidarian homeodomains. The consensus homeodomain sequence (shown several times for reference) was derived from a compilation of 247 human homeodomain sequences. The three horizontal lines indicate the positions of the three alpha-helices. The numbering scheme refers to amino acid position in the canonical 60-amino-acid homeodomain; insertions relative to this sequence are shown when present. Black shaded resides are invariant between all human homeodomains within each class (or family in the case of the ZF homeodomains). Sequence accession numbers are shown. For each gene family designation, maximum likelihood and neighbor-joining bootstrap support values are indicated (see Additional files 1 and 2). These values are not shown if the gene family does not form a monophyletic group in phylogenetic analyses (in which case n/a is written) or if an invertebrate homolog could not be found. [file 1741-7007-5-47-S6.pdf]

ANTP class

```
                    ______I______     ____II____    _______III_______
                   10        20        30        40        50        60
          ....|....|....|....|....|....|....|....|....|....|....|....|
```

		Sequence	Accession	ML bs	NJ bs
	**Consensus**	KRRKRTTFTKEQLLELEKEFAKNPYPSREEREELAASLGLTERQVKVWFQNRRAKWKKQE			

HOXL subclass

**Cdx**	**Hs CDX1**	KDKYRVVYTDHQRLELEKEFHYSRYITIRRKSELAANLGLTERQVKIWFQNRRAKERKVN	NP_001795	100	80
	**Hs CDX2**	KDKYRVVYTDHQRLELEKEFHYSRYITIRRKAELAATLGLSERQVKIWFQNRRAKERKIN	NP_001256		
	**Hs CDX4**	KEKYRVVYTDHQRLELEKEFHCNRYITIQRKSELAVNLGLSERQVKIWFQNRRAKERKMI	NP_005184		
	**Dm cad**	KDKYRVVYTDFQRLELEKEYCTSRYITIRRKSELAQTLSLSERQVKIWFQNRRAKERKQN	NP_476954		
**Evx**	**Hs EVX1**	MRRYRTAFTREQIARLEKEFYRENYVSRPRRCELAAALNLPETTIKVWFQNRRMKDKRQR	NP_001980	100	100
	**Hs EVX2**	VRRYRTAFTREQIARLEKEFYRENYVSRPRRCELAAALNLPETTIKVWFQNRRMKDKRQR	NP_001073927		
	**Dm eve**	VRRYRTAFTRDQLGRLEKEFYKENYVSRPRRCELAAQLNLPESTIKVWFQNRRMKDKRQR	NP_523670		
**Gbx**	**Hs GBX1**	SRRRRTAFTSEQLLELEKEFHCKKYLSLTERSQIAHALKLSEVQVKIWFQNRRAKWKRIK	NP_001092304	99	100
	**Hs GBX2**	NRRRRTAFTSEQLLELEKEFHCKKYLSLTERSQIAHALKLSEVQVKIWFQNRRAKWKRVK	NP_001476		
	**Dm unpg**	SRRRRTAFTSEQLLELEREFHAKKYLSLTERSQIATSLKLSEVQVKIWFQNRRAKWKRVK	NP_477146		
**Gsx**	**Hs GSX1**	SKRMRTAFTSTQLLELEREFASNMYLSRLRRIEIATYLNLSEKQVKIWFQNRRVKHKKEG	NP_663632	98	100
	**Hs GSX2**	GKRMRTAFTSTQLLELEREFSSNMYLSRLRRIEIATYLNLSEKQVKIWFQNRRVKHKKEG	NP_573574		
	**Dm ind**	SKRIRTAFTSTQLLELEREFSHNAYLSRLRRIEIANRLRLSEKQVKIWFQNRRVKQKKGG	NP_996087		
**Hox1**	**Hs HOXA1**	PNAVRTNFTTKQLTELEKEFHFNKYLTRARRVEIAASLQLNETQVKIWFQNRRMKQKKRE	NP_005513	98	99
	**Hs HOXB1**	PSGLRTNFTTRQLTELEKEFHFNKYLSRARRVEIAATLELNETQVKIWFQNRRMKQKKRE	NP_002135		
	**Hs HOXD1**	SSAIRTNFSTKQLTELEKEFHFNKYLTRARRIEIANCLHLNDTQVKIWFQNRRMKQKKRE	NP_078777		
	**Dm lab**	NNSGRTNFTNKQLTELEKEFHFNRYLTRARRIEIANTLQLNETQVKIWFQNRRMKQKKRV	NP_476613		
**Hox2**	**Hs HOXA2**	SRRLRTAYTNTQLLELEKEFHFNKYLCRPRRVEIAALLDLTERQVKVWFQNRRMKHKRQT	NP_006726	92	100
	**Hs HOXB2**	ARRLRTAYTNTQLLELEKEFHFNKYLCRPRRVEIAALLDLTERQVKVWFQNRRMKHKRQT	NP_002136		
	**Dm pb**	PRRLRTAYTNTQLLELEKEFHFNKYLCRPRRIEIAASLDLTERQVKVWFQNRRMKHKRQT	NP_476669		
**Hox3**	**Hs HOXA3**	SKRARTAYTSAQLVELEKEFHFNRYLCRPRRVEMANLLNLTERQIKIWFQNRRMKYKKDQ	NP_109377	69	68
	**Hs HOXB3**	SKRARTAYTSAQLVELEKEFHFNRYLCRPRRVEMANLLNLSERQIKIWFQNRRMKYKKDQ	NP_002137		
	**Hs HOXD3**	SKRVRTAYTSAQLVELEKEFHFNRYLCRPRRVEMANLLNLTERQIKIWFQNRRMKYKKDQ	NP_008829		
	**Sm Hox3b**	NKRSRTAYTQSQLVELEKEFHFNRYLCRPRRVELASMLNLTERQIKIWFQNRRMKNKKIK	ABN42908		
**Hox4**	**Hs HOXA4**	PKRSRTAYTRQQVLELEKEFHFNRYLTRRRRIEIAHTLCLSERQVKIWFQNRRMKWKKDH	NP_002132	n/a	39
	**Hs HOXB4**	PKRSRTAYTRQQVLELEKEFHYNRYLTRRRRVEIAHALCLSERQIKIWFQNRRMKWKKDH	NP_076920		
	**Hs HOXC4**	PKRSRTAYTRQQVLELEKEFHYNRYLTRRRRIEIAHSLCLSERQIKIWFQNRRMKWKKDH	NP_055435		
	**Hs HOXD4**	PKRSRTAYTRQQVLELEKEFHFNRYLTRRRRIEIAHTLCLSERQIKIWFQNRRMKWKKDH	NP_055436		
	**Dm Dfd**	PKRQRTAYTRHQILELEKEFHYNRYLTRRRRIEIAHTLVLSERQIKIWFQNRRMKWKKDN	NP_477201		
**Hox5**	**Hs HOXA5**	GKRARTAYTRYQTLELEKEFHFNRYLTRRRRIEIAHALCLSERQIKIWFQNRRMKWKKDN	NP_061975	n/a	28
	**Hs HOXB5**	GKRARTAYTRYQTLELEKEFHFNRYLTRRRRIEIAHALCLSERQIKIWFQNRRMKWKKDN	NP_002138		
	**Hs HOXC5**	GKRSRTSYTRYQTLELEKEFHFNRYLTRRRRIEIANNLCLNERQIKIWFQNRRMKWKKDS	NP_061826		
	**Dm Scr**	TKRQRTSYTRYQTLELEKEFHFNRYLTRRRRIEIAHALCLTERQIKIWFQNRRMKWKKEH	NP_524248		

**Hox6-8**	**Hs HOXA6**	GRRGRQTYTRYQTLELEKEFHFNRYLTRRRRIEIANALCLTERQIKIWFQNRRMKWKKEN	NP_076919	20	39
	**Hs HOXB6**	GRRGRQTYTRYQTLELEKEFHYNRYLTRRRRIEIAHALCLTERQIKIWFQNRRMKWKKES	NP_061825		
	**Hs HOXC6**	RRRGRQIYSRYQTLELEKEFHFNRYLTRRRRIEIANALCLTERQIKIWFQNRRMKWKKES	NP_004494		
	**Hs HOXA7**	RKRGRQTYTRYQTLELEKEFHFNRYLTRRRRIEIAHALCLTERQIKIWFQNRRMKWKKEH	NP_008827		
	**Hs HOXB7**	RKRGRQTYTRYQTLELEKEFHYNRYLTRRRRIEIAHTLCLTERQIKIWFQNRRMKWKKEN	NP_004493		
	**Hs HOXB8**	RRRGRQTYSRYQTLELEKEFLFNPYLTRKRRIEVSHALGLTERQVKIWFQNRRMKWKKEN	NP_076921		
	**Hs HOXC8**	RRSGRQTYSRYQTLELEKEFLFNPYLTRKRRIEVSHALGLTERQVKIWFQNRRMKWKKEN	NP_073149		
	**Hs HOXD8**	RRRGRQTYSRFQTLELEKEFLFNPYLTRKRRIEVSHALALTERQVKIWFQNRRMKWKKEN	NP_062458		
	**Dm Antp**	RKRGRQTYTRYQTLELEKEFHFNRYLTRRRRIEIAHALCLTERQIKIWFQNRRMKWKKEN	NP_996172		
	**Dm Ubx**	RRRGRQTYTRYQTLELEKEFHTNHYLTRRRRIEMAHALCLTERQIKIWFQNRRMKLKKEI	NP_536752		
	**Dm Abd-A**	RRRGRQTYTRFQTLELEKEFHFNHYLTRRRRIEIAHALCLTERQIKIWFQNRRMKLKKEL	NP_476693		
**Hox9-13**	**Hs HOXA9**	TRKKRCPYTKHQTLELEKEFLFNMYLTRDRRYEVARLLNLTERQVKIWFQNRRMKMKKIN	NP_689952	66	61
	**Hs HOXB9**	SRKKRCPYTKYQTLELEKEFLFNMYLTRDRRHEVARLLNLSERQVKIWFQNRRMKMKKMN	NP_076922		
	**Hs HOXC9**	TRKKRCPYTKYQTLELEKEFLFNMYLTRDRRYEVARVLNLTERQVKIWFQNRRMKMKKMN	NP_008828		
	**Hs HOXD9**	TRKKRCPYTKYQTLELEKEFLFNMYLTRDRRYEVARILNLTERQVKIWFQNRRMKMKKMS	NP_055028		
	**Hs HOXA10**	GRKKRCPYTKHQTLELEKEFLFNMYLTRERRLEISRSVHLTDRQVKIWFQNRRMKLKKMN	NP_061824		
	**Hs HOXC10**	GRKKRCPYTKHQTLELEKEFLFNMYLTRERRLEISKTINLTDRQVKIWFQNRRMKLKKMN	NP_059105		
	**Hs HOXD10**	GRKKRCPYTKHQTLELEKEFLFNMYLTRERRLEISKSVNLTDRQVKIWFQNRRMKLKKMS	NP_002139		
	**Hs HOXA11**	TRKKRCPYTKYQIRELEREFFFSVYINKEKRLQLSRMLNLTDRQVKIWFQNRRMKEKKIN	NP_005514		
	**Hs HOXC11**	TRKKRCPYSKFQIRELEREFFFNVYINKEKRLQLSRMLNLTDRQVKIWFQNRRMKEKKLS	NP_055027		
	**Hs HOXD11**	SRKKRCPYTKYQIRELEREFFFNVYINKEKRLQLSRMLNLTDRQVKIWFQNRRMKEKKLN	NP_067015		
	**Hs HOXC12**	SRKKRKPYSKLQLAELEGEFLVNEFITRQRRRELSDRLNLSDQQVKIWFQNRRMKKKRLL	NP_776272		
	**Hs HOXD12**	ARKKRKPYTKQQIAELENEFLVNEFINRQKRKELSNRLNLSDQQVKIWFQNRRMKKKRVV	NP_067016		
	**Hs HOXA13**	GRKKRVPYTKVQLKELEREYATNKFITKDKRRRISATTNLSERQVTIWFQNRRVKEKKVI	NP_000513		
	**Hs HOXB13**	GRKKRIPYSKGQLRELEREYAANKFITKDKRRKISAATSLSERQITIWFQNRRVKEKKVL	NP_006352		
	**Hs HOXC13**	GRKKRVPYTKVQLKELEKEYAASKFITKEKRRRISATTNLSERQVTIWFQNRRVKEKKVV	NP_059106		
	**Hs HOXD13**	GRKKRVPYTKLQLKELENEYAINKFINKDKRRRISAATNLSERQVTIWFQNRRVKDKKIV	NP_000514		
	**Dm Abd-B**	VRKKRKPYSKFQTLELEKEFLFNAYVSKQKRWELARNLQLTERQVKIWFQNRRMKNKKNS	NP_650577		
**Mnx**	**Hs MNX1**	CRRPRTAFTSQQLLELEHQFKFNKYLSRPKRFEVATSLMLTETQVKIWFQNRRMKWKRSK	NP_005506	90	99
	**Dm exex**	TRRPRTAFTSQQLLELEKQFKQNKYLSRPKRFEVASGLMLSETQVKIWFQNRRMKWKRSK	NP_648164		
**Meox**	**Hs MEOX1**	ARKERTAFTKEQLRELEAEFAHHNYLTRLRRYEIAVNLDLSERQVKVWFQNRRMKWKRVK	NP_004518	95	100
	**Hs MEOX2**	PRKERTAFTKEQIRELEAEFAHHNYLTRLRRYEIAVNLDLTERQVKVWFQNRRMKWKRVK	NP_005915		
	**Dm btn**	NRKERTAFSKTQLKQLEAEFCYSNYLTRLRRYEIAVALELTERQVKVWFQNRRMKCKRIK	NP_732768		
**Pdx**	**Hs PDX1**	NKRTRTAYTRAQLLELEKEFLFNKYISRPRRVELAVMLNLTERHIKIWFQNRRMKWKKEE	NP_000200	89	100
	**Ps Xlox**	NKRTRTAYTRAQLLELEKEFHFNKYISRPRRIELAAMLNLTERHIKIWFQNRRMKWKKDE	AAK77134		
**NKL subclass**					
**Barhl**	**Hs BARHL1**	PRKARTAFTDHQLAQLERSFERQKYLSVQDRMELAASLNLTDTQVKTWYQNRRTKWKRQT	NP_064448	95	94
	**Hs BARHL2**	PRKARTAFSDHQLNQLERSFERQKYLSVQDRMDLAAALNLTDTQVKTWYQNRRTKWKRQT	NP_064447		
	**Dm B-H1**	QRKARTAFTDHQLQTLEKSFERQKYLSVQERQELAHKLDLSDCQVKTWYQNRRTKWKRQT	NP_523387		
	**Dm B-H2**	QRKARTAFTDHQLQTLEKSFERQKYLSVQDRMELANKLELSDCQVKTWYQNRRTKWKRQT	NP_523386		
**Barx**	**Hs BARX1**	GRRSRTVFTELQLMGLEKRFEKQKYLSTPDRIDLAESLGLSQLQVKTWYQNRRMKWKKIV	NP_067545	prot/cnid	homolog not found
	**Hs BARX2**	PRRSRTIFTELQLMGLEKKFQKQKYLSTPDRLDLAQSLGLTQLQVKTWYQNRRMKWKKMV	NP_003649		
**Bsx**	**Hs BSX**	RRKARTVFSDSQLSGLEKRFEIQRYLSTPERVELATALSLSETQVKTWFQNRRMKHKKQL	NP_001091639	89	100
	**Dm bsh**	RRKARTVFSDPQLSGLEKRFEGQRYLSTPERVELATALGLSETQVKTWFQNRRMKHKKQL	NP_477350		

**Dbx**	**Hs DBX1**	GMLRRAVFSDVQRKALEKMFQKQKYISKPDRKKLAAKLGLKDSQVKIWFQNRRMKWRNSK	EAW68342	94	96
	**Hs DBX2**	GILRRAVFSEDQRKALEKMFQKQKYISKTDRKKLAINLGLKESQVKIWFQNRRMKWRNSK	NP_001004329		
	**Dm CG12361**	GMMRRAVFSDSQRKGLEKRFQQQKYISKPDRKKLAERLGLKDSQVKIWFQNRRMKWRNSK	NP_647677		
**Dlx**	**Hs DLX1**	IRKPRTIYSSLQLQALNRRFQQTQYLALPERAELAASLGLTQTQVKIWFQNKRSKFKKLM	NP_835221	100	100
	**Hs DLX2**	VRKPRTIYSSFQLAALQRRFQKTQYLALPERAELAASLGLTQTQVKIWFQNRRSKFKKMW	NP_004396		
	**Hs DLX3**	VRKPRTIYSSYQLAALQRRFQKAQYLALPERAELAAQLGLTQTQVKIWFQNRRSKFKKLY	NP_005211		
	**Hs DLX4**	LRKPRTIYSSLQLQHLNQRFQHTQYLALPERAQLAAQLGLTQTQVKIWFQNKRSKYKKLL	NP_612138		
	**Hs DLX5**	VRKPRTIYSSFQLAALQRRFQKTQYLALPERAELAASLGLTQTQVKIWFQNKRSKIKKIM	NP_005212		
	**Hs DLX6**	IRKPRTIYSSLQLQALNHRFQQTQYLALPERAELAASLGLTQTQVKIWFQNKRSKFKKLL	NP_005213		
	**Dm Dll**	MRKPRTIYSSLQLQQLNRRFQRTQYLALPERAELAASLGLTQTQVKIWFQNRRSKYKKMM	NP_523857		
**Emx**	**Hs EMX1**	PKRIRTAFSPSQLLRLERAFEKNHYVVGAERKQLAGSLSLSETQVKVWFQNRRTKYKRQK	NP_004088	92	100
	**Hs EMX2**	PKRIRTAFSPSQLLRLEHAFEKNHYVVGAERKQLAHSLSLTETQVKVWFQNRRTKFKRQK	NP_004089		
	**Dm ems**	PKRIRTAFSPSQLLKLEHAFESNQYVVGAERKALAQNLNLSETQVKVWFQNRRTKHKRMQ	NP_731868		
	**Dm E5**	PKRVRTAFSPTQLLKLEHAFEGNHYVVGAERKQLAQGLSLTETQVKVWFQNRRTKHKRMQ	NP_524825		
**En**	**Hs EN1**	DKRPRTAFTAEQLQRLKAEFQANRYITEQRRQTLAQELSLNESQIKIWFQNKRAKIKKAT	NP_001417	97	99
	**Hs EN2**	DKRPRTAFTAEQLQRLKAEFQTNRYLTEQRRQSLAQELSLNESQIKIWFQNKRAKIKKAT	NP_001418		
	**Dm en**	EKRPRTAFSSEQLARLKREFNENRYLTERRRQQLSSELGLNEAQIKIWFQNKRAKIKKST	NP_523700		
	**Dm inv**	DKRPRTAFSGTQLARLKHEFNENRYLTEKRRQQLSGELGLNEAQIKIWFQNKRAKLKKSS	NP_725055		
**Hhex**	**Hs HHEX**	RKGGQVRFSNDQTIELEKKFETQKYLSPPERKRLAKMLQLSERQVKTWFQNRRAKWRRLK	NP_002720	88	85
	**Dm CG7056**	RKGGQIRFTSQQTKNLEARFASSKYLSPEERRHLALQLKLTDRQVKTWFQNRRAKWRRAN	NP_650938		
**Hlx**	**Hs HLX**	RSWSRAVFSNLQRKGLEKRFEIQKYVTKPDRKQLAAMLGLTDAQVKVWFQNRRMKWRHSK	NP_068777	n/a	n/a
	**Dm H2.0**	KRKRSWSRAVFSNLQRKGLEIQQKYITKPDRRKLAARLNLTDAQVKVWFQNRRMKWRHTR	NP_523488		
**Lbx**	**Hs LBX1**	RRKSRTAFTNHQIYELEKRFLYQKYLSPADRDQIAQQLGLTNAQVITWFQNRRAKLKREL	NP_006553	95	94
	**Hs LBX2**	RRKSRTAFTAQQVLELERRFVFQKYLAPSERDGLATRLGLANAQVVTWFQNRRAKLKRDV	NP_001009812		
	**Dm lbe**	KRKSRTAFTNHQIFELEKRFLYQKYLSPADRDEIAASLGLSNAQVITWFQNRRAKQKRDI	NP_524435		
	**Dm lbl**	KRKSRTAFTNQQIFELEKRFLYQKYLSPADRDEIAGGLGLSNAQVITWFQNRRAKLKRDM	NP_524434		
**Msx**	**Hs MSX1**	NRKPRTPFTTAQLLALERKFRQKQYLSIAERAEFSSSLSLTETQVKIWFQNRRAKAKRLQ	NP_002439	98	100
	**Hs MSX2**	NRKPRTPFTTSQLLALERKFRQKQYLSIAERAEFSSSLNLTETQVKIWFQNRRAKAKRLQ	NP_002440		
	**Dm Dr**	NRKPRTPFTTQQLLSLEKKFREKQYLSIAERAEFSSSLRLTETQVKIWFQNRRAKAKRLQ	NP_477324		
**Nanog**	**Hs NANOG**	KQKTRTVFSSTQLCVLNDRFQRQKYLSLQQMQELSNILNLSYKQVKTWFQNQRMKSKRWQ	NP_079141	prot/cnid	homolog not found
**Nk1**	**Hs NKX1-1**	PRRARTAFTYEQLVALENKFKATRYLSVCERLNLALSLSLTETQVKIWFQNRRTKWKKQN	XP_931434	100	100
	**Hs NKX1-2**	PRRARTAFTYEQLVALENKFRATRYLSVCERLNLALSLSLTETQVKIWFQNRRTKWKKQN	XP_372331		
	**Dm slou**	PRRARTAFTYEQLVSLENKFKTTRYLSVCERLNLALSLSLTETQVKIWFQNRRTKWKKQN	NP_476657		
**Nk2-1**	**Hs NKX2-1**	RRKRRVLFSQAQVYELERRFKQQKYLSAPEREHLASMIHLTPTQVKIWFQNHRYKMKRQA	NP_001073136	62	79
	**Hs NKX2-4**	RRKRRVLFSQAQVYELERRFKQQKYLSAPEREHLASMIHLTPTQVKIWFQNHRYKMKRQA	CAH71494		
	**Dm scro**	RRKRRVLFTQAQVYELERRFKQQRYLSAPEREHLASLIHLTPTQVKIWFQNHRYKCKRQA	NP_001015473		
**Nk2-2**	**Hs NKX2-2**	KRKRRVLFSKAQTYELERRFRQQRYLSAPEREHLASLIRLTPTQVKIWFQNHRYKMKRAR	NP_002500	84	62
	**Hs NKX2-8**	RKKRRVLFSKAQTLELERRFRQQRYLSAPEREQLASLLRLTPTQVKIWFQNHRYKLKRAR	NP_055175		
	**Dm vnd**	KRKRRVLFTKAQTYELERRFRQQRYLSAPEREHLASLIRLTPTQVKIWFQNHRYKTKRAQ	NP_476786		
**Nk3**	**Hs NKX3-1**	QKRSRAAFSHTQVIELERKFSHQKYLSAPERAHLAKNLKLTETQVKIWFQNRRYKTKRKQ	NP_006158	79	88
	**Hs NKX3-2**	KKRSRAAFSHAQVFELERRFNHQRYLSGPERADLAASLKLTETQVKIWFQNRRYKTKRRQ	NP_001180		
	**Dm bap**	KKRSRAAFSHAQVFELERRFAQQRYLSGPERSEMAKSLRLTETQVKIWFQNRRYKTKRKQ	NP_732637		

**Nk4**	**Hs NKX2-3**	RRKPRVLFSQAQVFELERRFKQQRYLSAPEREHLASSLKLTSTQVKIWFQNRRYKCKRQR	NP_660328	n/a	60
	**Hs NKX2-5**	RRKPRVLFSQAQVYELERRFKQQRYLSAPERDQLASVLKLTSTQVKIWFQNRRYKCKRQR	NP_004378		
	**Hs NKX2-6**	RRKPRVLFSQAQVLALERRFKQQRYLSAPEREHLASALQLTSTQVKIWFQNRRYKCKRQR	XP_070619		
	**Pd NK4**	KRKPRVLFSQAQVYELERRFKQQRYLSAPEREQLASMLKLTSTQVKIWFQNRRYKMKRQR	ABQ10640		
**Nk5/Hmx**	**Hs HMX1**	KKKTRTVFSRSQVFQLESTFDLKRYLSTAERAGLAASLQLTETQVKIWFQNRRNKWKRHV	NP_061815	99	100
	**Hs HMX2**	KKKTRTVFSRSQVYQLESTFDMKRYLSSSERACLASSLQLTETQVKTWFQNRRNKWKRQL	NP_005510		
	**Hs HMX3**	KKKTRTVFSRSQVFQLESTFDMKRYLSSSERAGLAASLHLTETQVKIWFQNRRNKWKRQL	XP_291716		
	**Dm Hmx**	KKKTRTVFSRAQVFQLESTFDLKRYLSSSERAGLAASLRLTETQVKIWFQNRRNKWKRQL	NP_524951		
**Nk6**	**Hs NKX6-1**	RKHTRPTFSGQQIFALEKTFEQTKYLAGPERARLAYSLGMTESQVKVWFQNRRTKWRKKH	NP_006159	100	100
	**Hs NKX6-2**	KKHSRPTFSGQQIFALEKTFEQTKYLAGPERARLAYSLGMTESQVKVWFQNRRTKWRKRH	NP_796374		
	**Hs NKX6-3**	KKHTRPTFTGHQIFALEKTFEQTKYLAGPERARLAYSLGMTESQVKVWFQNRRTKWRKKS	EAW63251		
	**Dm HGTX**	KKHTRPTFSGQQIFALEKTFEQTKYLAGPERAKLAYALGMSESQVKVWFQNRRTKWRKRH	NP_652614		
**Noto**	**Hs NOTO**	QKRVRTMFNLEQLEELEKVFAKQHNLVGKKRAQLAARLKLTENQVRVWFQNRRVKYQKQQ	XP_292889	prot/cnid homolog not found	
**Tlx**	**Hs TLX1**	KKKPRTSFTRLQICELEKRFHRQKYLASAERAALAKALKMTDAQVKTWFQNRRTKWRRQT	NP_005512	98	100
	**Hs TLX2**	RKKPRTSFSRSQVLELERRFLRQKYLASAERAALAKALRMTDAQVKTWFQNRRTKWRRQT	NP_057254		
	**Hs TLX3**	RKKPRTSFSRVQICELEKRFHRQKYLASAERAALAKSLKMTDAQVKTWFQNRRTKWRRQT	NP_066305		
	**Dm C15**	RKKPRTSFTRIQVAELEKRFHKQKYLASAERAALARGLKMTDAQVKTWFQNRRTKWRRQT	NP_476873		
**Vax**	**Hs VAX1**	PKRTRTSFTAEQLYRLEMEFQRCQYVVGRERTELARQLNLSETQVKVWFQNRRTKQKKDQ	CAI14825	prot/cnid homolog not found	
	**Hs VAX2**	PKRTRTSFTAEQLYRLEMEFQRCQYVVGRERTELARQLNLSETQVKVWFQNRRTKQKKDQ	NP_036608		
**Ventx**	**Hs VENTX**	APRVRTAFTMEQVRTLEGVFQHHQYLSPLERKRLAREMQLSEVQIKTWFQNRRMKHKRQM	NP_055283	prot/cnid homolog not found	

PRD class

```
             ______I______      ____II____    _______III_______
             10        20         30        40        50        60
....|....|....|....|... .|....|....|....|....|....|....|....|
```

	**Consensus**	KRRKRTTFTKEQLLELEKEFAKN-PYPSREEREELAASLGLTERQVKVWFQNRRAKWKKQE	**Accession**	**ML bs**	**NJ bs**
**Alx**	**Hs ALX1**	KRRHRTTFTSLQLEELEKVFQKT-HYPDVYVREQLALRTELTEARVQVWFQNRRAKWRKRE	NP_008913	n/a	79
	**Hs ALX3**	KRRNRTTFSTFQLEELEKVFQKT-HYPDVYAREQLALRTDLTEARVQVWFQNRRAKWRKRE	NP_006483		
	**Hs ALX4**	KRRNRTTFTSYQLEELEKVFQKT-HYPDVYAREQLAMRTDLTEARVQVWFQNRRAKWRKRE	NP_068745		
	**Nv CART1**	KRRNRTTFTAYQLEEMERVFQKT-HYPDVYTREQLALRCALTEARVQVWFQNRRAKWRKRE	ABB83749		
**Argfx**	**Hs ARGFX**	RHKERTSFTHQQYEELEALFSQT-MFPDRNLQEKLALRLDLPESTVKVWFRNRRFKLKKQQ	NP_001012677	prot/cnid homolog not found	
**Arx**	**Hs ARX**	QRRYRTTFTSYQLEELERAFQKT-HYPDVFTREELAMRLDLTEARVQVWFQNRRAKWRKRE	NP_620689	23	28
	**Dm al**	QRRYRTTFTSFQLEELEKAFSRT-HYPDVFTREELAMKIGLTEARIQVWFQNRRAKWRKQE	NP_722629		
	**Dm Pph13**	QRRYRTTFNTLQLQELERAFQRT-HYPDVFFREELAVRIDLTEARVQVWFQNRRAKWRKQE	NP_477330		
**Dmbx**	**Hs DMBX1**	QRRSRTAFTAQQLEALEKTFQKT-HYPDVVMRERLAMCTNLPEARVQVWFKNRRAKFRKKQ	NP_671725	50	49
	**Hv manacle**	HRRVRTAFTHHQLTTLERTFETS-HYPDVVLRERLASFTGLAESRIQVWFKNRRAKYRKHQ	AAD30998		
**Dprx**	**Hs DPRX**	SHRKRTMFTKKQLEDLNILFNEN-PYPNPSLQKEMASKIDIHPTVLQVWFKNHRAKLKKAK	NP_001012746	prot/cnid homolog not found	

**Drgx**	**Hs DRGX**	QRRNRTTFTLQQLEALEAVFAQT-HYPDVFTREELAMKINLTEARVQVWFQNRRAKWRKTE	NP_001073989	90	97
	**Dm IP09201**	QRRNRTTFTLQQLEELETAFAQT-HYPDVFTREDLAMKINLTEARVQVWFQNRRAKWRKAE	ABK30908		
**Dux**	**Hs DUXA-I**	HRRCRTKFTEEQLKILINTFNQK-PYPGYATKQKLALEINTEESRIQIWFQNRRARHGFQK	NP_001012747	prot/cnid homolog not found	
	**Hs DUXA-II**	ARRCRTTYSASQLHTLIKAFMKN-PYPGIDSREELAKEIGVPESRVQIWFQNRRSRLLLQR	NP_001012747		
	**Hs DUXB-I**	FWRNRIQYNQSQKDILQSWFQHD-PFPDKAAREQLAKEIGVPESNIQVWFKNYRVKQRKLD	unassigned		
	**Hs DUXB-II**	ARQKQTFITWTQKNRLVQAFERN-PFPDIATRKKLAEQTGLQESRIQMWFQKQRSLYLKKS	unassigned		
**Esx**	**Hs ESX1**	KRRRRTAFTQFQLQELENFFDES-QYPDVVARERLAARLNLTEDRVQVWFQNRRAKWKRNQ	NP_703149	prot/cnid homolog not found	
**Gsc**	**Hs GSC**	KRRHRTIFTDEQLEALENLFQET-KYPDVGTREQLARKVHLREEKVEVWFKNRRAKWRRQK	NP_776248	74	77
	**Hs GSC2**	TRRHRTIFSEEQLQALEALFVQN-QYPDVSTRERLAGRIRLREERVEVWFKNRRAKWRHQK	NP_005306		
	**Dm Gsc**	KRRHRTIFTEEQLEQLEATFDKT-HYPDVVLREQLALKVDLKEERVEVWFKNRRAKWRKQK	NP_476949		
**Hesx**	**Hs HESX1**	GRRPRTAFTQNQIEVLENVFRVN-CYPGIDIREDLAQKLNLEEDRIQIWFQNRRAKLKRSH	NP_003856	prot/cnid homolog not found	
**Hopx**	**Hs HOPX**	SAETASGPTEDQVEILEYNFNKVDKHPDSTTLCLIAAEAGLSEEETQKWFKQRLAKWRRSE	NP_115884	prot/cnid homolog not found	
**Isx**	**Hs ISX**	KRRVRTTFTTEQLHELEKIFHFT-HYPDVHIRSQLAARINLPEARVQIWFQNQRAKWRKQE	NP_001008494	prot/cnid homolog not found	
**Leutx**	**Hs LEUTX**	YRRPRTRFLSKQLTALRELLEKT-MHPSLATMGKLASKLQLDLSVVKIWFKNQRAKWKRQQ	XP_001129035	prot/cnid homolog not found	
**Mix**	**Hs MIXL**	QRRKRTSFSAEQLQLLELVFRRT-RYPDIHLRERLAALTLLPESRIQVWFQNRRAKSRRQS	NP_114150	prot/cnid homolog not found	
**Nobox**	**Hs NOBOX**	RKKTRTLYRSDQLEELEKIFQED-HYPDSDKRREIAQTVGVTPQRIMVWFQNRRAKWRKME	O60393	prot/cnid homolog not found	
**Otp**	**Hs OTP**	QKRHRTRFTPAQLNELERSFAKT-HYPDIFMREELALRIGLTESRVQVWFQNRRAKWKKRK	NP_115485	100	99
	**Dm otp**	QKRHRTRFTPAQLNELERCFSKT-HYPDIFMREEIAMRIGLTESRVQVWFQNRRAKWKKRK	NP_523799		
**Otx**	**Hs OTX1**	QRRERTTFTRSQLDVLEALFAKT-RYPDIFMREEVALKINLPESRVQVWFKNRRAKCRQQQ	NP_055377	91	98
	**Hs OTX2**	QRRERTTFTRAQLDVLEALFAKT-RYPDIFMREEVALKINLPESRVQVWFKNRRAKCRQQQ	NP_068374		
	**Hs CRX**	QRRERTTFTRSQLEELEALFAKT-QYPDVYAREEVALKINLPESRVQVWFKNRRAKCRQQR	NP_000545		
	**Dm oc**	QRRERTTFTRAQLDVLEALFGKT-RYPDIFMREEVALKINLPESRVQVWFKNRRAKCRQQL	NP_511091		
**Pax2/5/8**	**Hs PAX2**	KHLRADTFTQQQLEALDRVFERP-SYPDVFQASE--------------------------	NP_003981	partial homeodomain	
	**Hs PAX5**	KQMRGDLFTQQQLEVLDRVFERQ-HYSDIFTTTE--------------------------	NP_057953		
	**Hs PAX8**	KHLRTDAFSQHHLEPLECPF RQ-HYPEAYASPS--------------------------	NP_003457		
**Pax3/7**	**Hs PAX3**	QRRSRTTFTAEQLEELERAFERT-HYPDIYTREELAQRAKLTEARVQVWFSNRRARWRKQA	NP_852122	78	79
	**Hs PAX7**	QRRSRTTFTAEQLEELEKAFERT-HYPDIYTREELAQRTKLTEARVQVWFSNRRARWRKQA	NP_002575		
	**Dm prd**	QRRCRTTFSASQLDELERAFERT-QYPDIYTREELAQRTNLTEARIQVWFSNRRARLRKQH	NP_523556		
	**Dm gsb**	QRRSRTTFSNDQIDALERIFART-QYPDVYTREELAQSTGLTEARVQVWFSNRRARLRKQL	NP_523863		
	**Dm gsb-n**	QRRSRTTFTAEQLEALERAFSRT-QYPDVYTREELAQTTALTEARIQVWFSNRRARLRKHS	NP_523862		
**Pax4/6**	**Hs PAX4**	GHRNRTIFSPSQAEALEKEFQRG-QYPDSVARGKLATATSLPEDTVRVWFSNRRAKWRRQE	NP_006184	28	29
	**Hs PAX6**	LQRNRTSFTQEQIEALEKEFERT-HYPDVFARERLAAKIDLPEARIQVWFSNRRAKWRREE	NP_001595		
	**Dm ey**	LQRNRTSFTNDQIDSLEKEFERT-HYPDVFARERLAGKIGLPEARIQVWFSNRRAKWRREE	NP_524628		
	**Dm eyg**	FRRNRTTFSPEQLEELEKEFDKS-HYPCVSTRERLSSRTSLSEARVQVWFSNRRAKWRRHQ	NP_524042		
	**Dm toy**	LQRNRTSFSNEQIDSLEKEFERT-HYPDVFARERLADKIGLPEARIQVWFSNRRAKWRREE	NP_524638		
	**Dm toe**	FRRNRTTFSPEQLDELEKEFDKS-HYPCVNTREKLAARTALSEARVQVWFSNRRAKWRRHQ	NP_524041		
**Phox**	**Hs PHOX2A**	QRRIRTTFTSAQLKELERVFAET-HYPDIYTREELALKIDLTEARVQVWFQNRRAKFRKQE	NP_005160	75	71
	**Hs PHOX2B**	QRRIRTTFTSAQLKELERVFAET-HYPDIYTREELALKIDLTEARVQVWFQNRRAKFRKQE	NP_003915		
	**Dm PHDP**	QRRIRTTFTSNQLNELEKIFLET-HYPDIYTREEIASKLHLTEARVQVWFQNRRAKFRKQE	NP_523834		

**Pitx**	**Hs PITX1**	QRRQRTHFTSQQLQELEATFQRN-RYPDMSMREEIAVWTNLTEPRVRVWFKNRRAKWRKRE	NP_002644	100	100
	**Hs PITX2**	QRRQRTHFTSQQLQELEATFQRN-RYPDMSTREEIAVWTNLTEARVRVWFKNRRAKWRKRE	NP_000316		
	**Hs PITX3**	QRRQRTHFTSQQLQELEATFQRN-RYPDMSTREEIAVWTNLTEARVRVWFKNRRAKWRKRE	NP_005020		
	**Dm Ptx1**	QRRQRTHFTSQQLQELEHTFSRN-RYPDMSTREEIAMWTNLTEARVRVWFKNRRAKWRKRE	NP_733410		
**Prop**	**Hs PROP1**	RRRHRTTFSPVQLEQLESAFGRN-QYPDIWARESLARDTGLSEARIQVWFQNRRAKQRKQE	NP_006252	52	n/a
	**Dm CG32532**	RRRHRTTFTQEQLAELEAAFAKS-HYPDIYCREELARTTKLNEARIQVWFQNRRAKYRKQE	NP_608318		
**Prrx**	**Hs PRRX1**	QRRNRTTFNSSQLQALERVFERT-HYPDAFVREDLARRVNLTEARVQVWFQNRRAKFRRNE	NP_073207	92	81
	**Hs PRRX2**	QRRNRTTFNSSQLQALERVFERT-HYPDAFVREELARRVNLSEARVQVWFQNRRAKFRRNE	NP_057391		
	**Dm CG9876**	PRRNRTTFSSAQLTALEKVFERT-HYPDAFVREELATKVHLSEARVQVWFQNRRAKFRRNE	NP_611756		
**Rax**	**Hs RAX**	HRRNRTTFTTYQLHELERAFEKS-HYPDVYSREELAGKVNLPEVRVQVWFQNRRAKWRRQE	NP_038463	94	99
	**Hs RAX2**	HRRNRTTFTTYQLHQLERAFEAS-HYPDVYSREELAAKVHLPEVRVQVWFQNRRAKWRRQE	NP_116142		
	**Dm Rx**	HRRNRTTFTTYQLHELERAFEKS-HYPDVYSREELAMKVNLPEVRVQVWFQNRRAKWRRQE	NP_726006		
**Rhox**	**Hs RHOXF1**	PRTRRTKFTLLQVEELESVFRHT-QYPDVPTRRELAENLGVTEDKVRVWFKNKRARCRRHQ	NP_644811	prot/cnid homolog not found	
	**Hs RHOXF2**	QQPNVHAFTPLQLQELERIFQRE-QFPSEFLRRRLARSMNVTELAVQIWFENRRAKWRRHQ	NP_115887		
	**Hs RHOXF2B**	QQPNVHAFTPLQLQELECIFQRE-QFPSEFLRRRLARSMNVTELAVQIWFENRRAKWRRHQ	NP_001093155		
**Sebox**	**Hs SEBOX**	HRRKRTTFSKGQLLELERAFAAW-PYPNISTHEHLAWVTCLPEAKVQVWFQKRWAKIIKNR	NP_001074306	prot/cnid homolog not found	
**Shox**	**Hs SHOX**	QRRSRTNFTLEQLNELERLFDET-HYPDAFMREELSQRLGLSEARVQVWFQNRRAKCRKQE	NP_000442	100	100
	**Hs SHOX2**	QRRSRTNFTLEQLNELERLFDET-HYPDAFMREELSQRLGLSEARVQVWFQNRRAKCRKQE	NP_006875		
	**Dm IP17602**	QRRSRTNFTLDQLNELERLFEET-HYPDAFMREELSQRLGLSEARVQVWFQNRRAKCRKHE	ABM92832		
**Tprx**	**Hs TPRX1**	QRQERTVYTESQQKVLEFYFQKD-QYPNYDQRLNLAEMLSLREQQLQVWFKNRRAKLARER	EAW57514	prot/cnid homolog not found	
	**Hs TPRXL**	QRQDRTIYNWKQQEVLENHFKEE-QYPDYDTRQELAEMLNLREYQVQVWFKNRRAKRSRER	unassigned		
**Uncx**	**Hs UNCX**	RRRTRTNFTGWQLEELEKAFNES-HYPDVFMREALALRLDLVESRVQVWFQNRRAKWRKKE	NP_001073930	90	85
	**Dm unc-4**	RRRSRTNFNSWQLEELERAFSAS-HYPDIFMREALAMRLDLKESRVAVWFQNRRAKVRKRE	NP_573242		
	**Dm OdsH**	KRRGRTNFNSWQLRELERVFQGS-HYPDIFMREALATKLDLMEGRIAVWFQNRRAKWRKQE	NP_523389		
**Vsx**	**Hs VSX1**	KRRHRTVFTAHQLEELEKAFSEA-HYPDVYAREMLAVKTELPEDRIQVWFQNRRAKWRKRE	NP_055403	97	100
	**Hs VSX2**	KRRHRTIFTSYQLEELEKAFNEA-HYPDVYAREMLAMKTELPEDRIQVWFQNRRAKWRKRE	NP_878314		
	**Dm CG33980**	RRHSRTIFTSYQLEKLEEAFKEA-HYPDVYAREMLSLKTELPEDRIQVWFQNRRAKWRKTE	NP_001033832		
	**Dm CG4136**	RRHGRTIFTSSQLEELEKAFKEA-HYPDVSARELLSMKTGLAEDRIQVWYQNRRAKWRKTE	NP_572232		

LIM class

```
                  ______I______    ____II____    ________III________
                  10        20        30        40        50        60
         ....|....|....|....|....|....|....|....|....|....|....|....|
```

	**Consensus**	KRRKRTTFTKEQLLELEKEFAKNPYPSREEREELAASLGLTERQVKVWFQNRRAKWKKQE	**Accession**	**ML bs**	**NJ bs**
**Isl**	**Hs ISL1**	TTRVRTVLNEKQLHTLRTCYAANPRPDALMKEQLVEMTGLSPRVIRVWFQNKRCKDKKRS	NP_002193	100	100
	**Hs ISL2**	TTRVRTVLNEKQLHTLRTCYAANPRPDALMKEQLVEMTGLSPRVIRVWFQNKRCKDKKKS	NP_665804		
	**Dm tup**	PTRVRTVLNEKQLHTLRTCYNANPRPDALMKEQLVEMTSLSPRVIRVWFQNKRCKDKKKT	NP_476775		

**Lhx1/5**	**Hs LHX1**	RRGPRTTIKAKQLETLKAAFAATPKPTRHIREQLAQETGLNMRVIQVWFQNRRSKERRMK	NP_005559	96	89
	**Hs LHX5**	RRGPRTTIKAKQLETLKAAFAATPKPTRHIREQLAQETGLNMRVIQVWFQNRRSKERRMK	NP_071758		
	**Dm Lim1**	RRGPRTTIKAKQLEVLKTAFNQTPKPTRHIREQLAKETGLPMRVIQVWFQNKRSKERRMK	NP_572505		
**Lhx2/9**	**Hs LHX2**	TKRMRTSFKHHQLRTMKSYFAINHNPDAKDLKQLAQKTGLTKRVLQVWFQNARAKFRRNL	NP_004780	98	100
	**Hs LHX9**	TKRMRTSFKHHQLRTMKSYFAINHNPDAKDLKQLAQKTGLTKRVLQVWFQNARAKFRRNL	NP_064589		
	**Dm ap**	TKRMRTSFKHHQLRTMKSYFAINHNPDAKDLKQLSQKTGLPKRVLQVWFQNARAKWRRMM	NP_724428		
**Lhx3/4**	**Hs LHX3**	AKRPRTTITAKQLETLKSAYNTSPKPARHVREQLSSETGLDMRVVQVWFQNRRAKEKRLK	NP_055379	94	99
	**Hs LHX4**	AKRPRTTITAKQLETLKNAYKNSPKPARHVREQLSSETGLDMRVVQVWFQNRRAKEKRLK	NP_203129		
	**Dm Lim3**	NKRPRTTITAKQLETLKTAYNNSPKPARHVREQLSQDTGLDMRVVQVWFQNRRAKEKRLK	NP_476606		
**Lhx6/8**	**Hs LHX6**	AKRARTSFTAEQLQVMQAQFAQDNNPDAQTLQKLADMTGLSRRVIQVWFQNCRARHKKHT	NP_055183	58	54
	**Hs LHX8**	AKRARTSFTADQLQVMQAQFAQDNNPDAQTLQKLAERTGLSRRVIQVWFQNCRARHKKHV	NP_001001933		
	**Dm Awh**	TKRVRTTFTEEQLQVLQANFQIDSNPDGQDLERIASVTGLSKRVTQVWFQNSRARQKKHI	NP_523907		
**Lmx**	**Hs LMX1A**	PKRPRTILTTQQRRAFKASFEVSSKPCRKVRETLAAETGLSVRVVQVWFQNQRAKMKKLA	NP_796372	100	100
	**Hs LMX1B**	PKRPRTILTTQQRRAFKASFEVSSKPCRKVRETLAAETGLSVRVVQVWFQNQRAKMKKLA	NP_002307		
	**Dm CG32105**	PKRPRTILTSQQRKQFKASFDQSPKPCRKVREALAKDTGLSVRVVQVWFQNQRAKMKKIQ	NP_729801		
	**Dm CG4328**	PKRPRTILNTQQRRAFKASFEVSPKPCRKVRENLAKDTGLSLRIVQVWFQNQRAKVKKIQ	NP_648567		

POU class

```
             ______I______          ____II____     _______III_______
             10        20             30        40        50        60
....    |....|....|....|....|    ....|....|....|....|....|....|....|
```

	**Consensus**	KRRK----RTTFTKEQLLELEKEFAKNPY----PSREEREELAASLGLTERQVKVWFQNRRAKWKKQE	**Accession**	**ML bs**	**NJ bs**
**Hdx**	**Hs HDX**	SRKRALQDRTQFSDRDLATLKKYWDNGMTSLGSVCREKIEAVATELNVDCEIVRTWIGNRRRKYRLMG	NP_653258	prot/cnid	homolog not found
**Pou1**	**Hs POU1F1**	KRKR----RTTISIAAKDALERHFGEQNK----PSSQEIMRMAEELNLEKEVVRVWFCNRRQREKRVK	NP_000297	94	90
	**Nv POU1**	RRKR----RTTIGLAAKEALENHFMKQTK----PSSPEIVRIADGLRLDKEVVRVWFCNRRQREKRVK	ABB86471		
**Pou2**	**Hs POU2F1**	RRKK----RTSIETNIRVALEKSFLENQK----PTSEEITMIADQLNMEKEVIRVWFCNRRQKEKRIN	NP_002688	83	79
	**Hs POU2F2**	RRKK----RTSIETNVRFALEKSFLANQK----PTSEEILLIAEQLHMEKEVIRVWFCNRRQKEKRIN	NP_002689		
	**Hs POU2F3**	KRKK----RTSIETNIRLTLEKRFQDNPK----PSSEEISMIAEQLSMEKEVVRVWFCNRRQKEKRIN	NP_055167		
	**Dm nub**	RRKK----RTSIETTIRGALEKAFLANQK----PTSEEITQLADRLSMEKEVVRVWFCNRRQKEKRIN	NP_476659		
	**Dm pdm2**	RRKK----RTSIETTVRTTLEKAFLMNCK----PTSEEISQLSERLNMDKEVIRVWFCNRRQKEKRIN	NP_523558		
**Pou3**	**Hs POU3F1**	KRKK----RTSIEVGVKGALESHFLKCPK----PSAHEITGLADSLQLEKEVVRVWFCNRRQKEKRMT	NP_002690	83	93
	**Hs POU3F2**	KRKK----RTSIEVSVKGALESHFLKCPK----PSAQEITSLADSLQLEKEVVRVWFCNRRQKEKRMT	NP_005595		
	**Hs POU3F3**	KRKK----RTSIEVSVKGALESHFLKCPK----PSAQEITNLADSLQLEKEVVRVWFCNRRQKEKRMT	NP_006227		
	**Hs POU3F4**	KRKK----RTSIEVSVKGVLETHFLKCPK----PAAQEISSLADSLQLEKEVVRVWFCNRRQKEKRMT	NP_000298		
	**Dm vvl**	KRKK----RTSIEVSVKGALEQHFHKQPK----PSAQEITSLADSLQLEKEVVRVWFCNRRQKEKRMT	NP_523948		
**Pou4**	**Hs POU4F1**	KKRK----RTSIAAPEKRSLEAYFAVQPR----PSSEKIAAIAEKLDLKKNVVRVWFCNQRQKQKRMK	NP_006228	95	100
	**Hs POU4F2**	KKRK----RTSIAAPEKRSLEAYFAIQPR----PSSEKIAAIAEKLDLKKNVVRVWFCNQRQKQKRMK	NP_004566		
	**Hs POU4F3**	RKRK----RTSIAAPEKRSLEAYFAIQPR----PSSEKIAAIAEKLDLKKNVVRVWFCNQRQKQKRMK	NP_002691		
	**Dm acj6**	KKRK----RTSIAAPEKRSLEAYFAVQPR----PSGEKIAAIAEKLDLKKNVVRVWFCNQRQKQKRIV	NP_524876		

```
Pou5    Hs POU5F1   RKRK----RTSIENRVRGNLENLFLQCPK----PTLQQISHIAQQLGLEKDVVRVWFCNRRQKGKRSS   NP_002692   prot/cnid homolog not found
        Hs POU5F2   GKWR----RASRERRIGNSLEKFFQRCPK----PTPQQISHIAGCLQLQKDVVRVWFYNRSKMGSRPT   NP_694948

Pou6    Hs POU6F1   KRKR----RTSFTPQAIEALNAYFEKNPL----PTGQEITEIAKELNYDREVVRVWFCNRRQTLKNTS   NP_002693   89   92
        Hs POU6F2   KRKR----RTSFTPQALEILNAHFEKNTH----PSGQEMTEIAEKLNYDREVVRVWFCNKRQALKNTI   NP_009183
        Dm pdm3     KRKR----RTSFTPQALELLNAHFERNTH----PSGTEITGLAHQLGYEREVIRIWFCNKRQALKNTV   NP_610377
```

HNF class

```
                             ______I______     ____II____                               _______III_______
                             10        20        30                             40        50        60
                    ....|....|....|....|....|....|....|....                     |....|....|....|....|
            Consensus   KRRKRTTFTKEQLLELEKEFAKNPYPSREEREELAASLG--------------------LTERQVKVWFQNRRAKWKKQE   Accession   ML bs   NJ bs

Hmbox   Hs HMBOX1   RRGSRFTWRKECLAVMESYFNENQYPDEAKREEIANACN--AVIQK---PGKKLS-DLERVTSLKVYNWFANRRKEIKRRA   NP_078843   prot/cnid homolog not found

Hnf1    Hs HNF1A    GRRNRFKWGPASQQILFQAYERQKNPSKEERETLVEECNRAECIQRGVSPSQAQGLGSNLVTEVRVYNWFANRRKEEAFRH   NP_000536   99   98
        Hs HNF1B    MRRNRFKWGPASQQILYQAYDRQKNPSKEEREALVEECNRAECLQRGVSPSKAHGLGSNLVTEVRVYNWFANRRKEEAFRQ   NP_000449
        Nv HNF      SRRNRFKWGPASTNILYQSYEQQRNPSKEEREALVEACNRAECEQRGVSYNNVEGLGFNLVTESRVYNWFANRRKEETFRM   ABB86496
```

SINE class

```
                             ______I______     ____II____     _______III_______
                             10        20        30        40        50        60
                    ....|....|....|....|....|....|....|....|....|....|....|....|
            Consensus   KRRKRTTFTKEQLLELEKEFAKNPYPSREEREELAASLGLTERQVKVWFQNRRAKWKKQE   Accession   ML bs   NJ bs

Six1/2  Hs SIX1     GEETSYCFKEKSRGVLREWYAHNPYPSPREKRELAEATGLTTTQVSNWFKNRRQRDRAAE   NP_005973   81    100
        Hs SIX2     GEETSYCFKEKSRSVLREWYAHNPYPSPREKRELTEATGLTTTQVSNWFKNRRQRDRAAE   NP_058628
        Dm so       GEETSYCFKEKSRSVLRDWYSHNPYPSPREKRDLAEATGLTTTQVSNWFKNRRQRDRAAE   NP_476733

Six3/6  Hs SIX3     GEQKTHCFKERTRSLLREWYLQDPYPNPSKKRELAQATGLTPTQVGNWFKNRRQRDRAAA   NP_005404   100   99
        Hs SIX6     GEQKTHCFKERTRNLLREWYLQDPYPNPSKKRELAQATGLTPTQVGNWFKNRRQRDRAAA   NP_031400
        Dm Optix    GEQKTHCFKERTRSLLREWYLQDPYPNPTKKRELAKATGLNPTQVGNWFKNRRQRDRAAA   NP_524695

Six4/5  Hs SIX4     GEETVYCFKEKSRNALKELYKQNRYPSPAEKRHLAKITGLSLTQVSNWFKNRRQRDRNPS   NP_059116   90    91
        Hs SIX5     GEETVYCFKERSRAALKACYRGNRYPTPDEKRRLATLTGLSLTQVSNWFKNRRQRDRTGA   NP_787071
        Dm Six4     GEETVYCFKEKSRNALKDCYLTNRYPTPDEKKTLAKKTGLTLTQVSNWFKNRRQRDRTPQ   NP_649256
```

TALE class

```
                  ______I______        ____II____    ________III________
                  10        20           30        40        50        60
               ....|....|....|....|...   .|....|....|....|....|....|....|
```

	**Consensus**	KRRKRTTFTKEQLLELEKEFAKN---PYPSREEREELAASLGLTERQVKVWFQNRRAKWKKQE	**Accession**	**ML bs**	**NJ bs**
**Irx**	**Hs IRX1**	DPGRPKNATRESTSTLKAWLNEHRKNPYPTKGEKIMLAIITKMTLTQVSTWFANARRRLKKEN	NP_077313	97	38
	**Hs IRX2**	DPAYRKNATRDATATLKAWLNEHRKNPYPTKGEKIMLAIITKMTLTQVSTWFANARRRLKKEN	NP_150366		
	**Hs IRX3**	DPSRPKNATRESTSTLKAWLNEHRKNPYPTKGEKIMLAIITKMTLTQVSTWFANARRRLKKEN	NP_077312		
	**Hs IRX4**	SGTRRKNATRETTSTLKAWLQEHRKNPYPTKGEKIMLAIITKMTLTQVSTWFANARRRLKKEN	NP_057442		
	**Hs IRX5**	DPAYRKNATRDATATLKAWLNEHRKNPYPTKGEKIMLAIITKMTLTQVSTWFANARRRLKKEN	NP_005844		
	**Hs IRX6**	GAGRRKNATRETTSTLKAWLNEHRKNPYPTKGEKIMLAIITKMTLTQVSTWFANARRRLKKEN	NP_077311		
	**Dm ara**	LAARRKNATRESTATLKAWLNEHKKNPYPTKGEKIMLAIITKMTLTQVSTWFANARRRLKKEN	NP_524045		
	**Dm caup**	LAARRKNATRESTATLKAWLSEHKKNPYPTKGEKIMLAIITKMTLTQVSTWFANARRRLKKEN	NP_524046		
	**Dm mirr**	NGARRKNATRETTSTLKAWLNEHKKNPYPTKGEKIMLAIITKMTLTQVSTWFANARRRLKKEN	NP_524047		
**Meis**	**Hs MEIS1**	RHKKRGIFPKVATNIMRAWLFQHLTHPYPSEEQKKQLAQDTGLTILQVNNWFINARRRIVQPM	NP_002389	69	93
	**Hs MEIS2**	RQKKRGIFPKVATNIMRAWLFQHLTHPYPSEEQKKQLAQDTGLTILQVNNWFINARRRIVQPM	NP_733777		
	**Hs MEIS3**	RNKKRGIFPKVATNIMRAWLFQHLSHPYPSEEQKKQLAQDTGLTILQVNNWFINARRRIVQPM	NP_001009813		
	**Dm hth**	NQKKRGIFPKVATNILRAWLFQHLTHPYPSEDQKKQLAQDTGLTILQVNNWFINARRRIVQPM	NP_476578		
**Mkx**	**Hs MKX**	KVRHKRQALQDMARPLKQWLYKHRDNPYPTKTEKILLALGSQMTLVQVSNWFANARRRLKNTV	NP_775847	n/a	n/a
	**Dm CG11617**	SRATKRLFTPDIKRMLKDWLIRRRENPYPSREEKKQLAAETGLTYTQICNWFANWRRKLKNSE	NP_608502		
**Pbx**	**Hs PBX1**	ARRKRRNFNKQATEILNEYFYSHLSNPYPSEEAKEELAKKCGITVSQVSNWFGNKRIRYKKNI	NP_002576	100	100
	**Hs PBX2**	ARRKRRNFSKQATEVLNEYFYSHLSNPYPSEEAKEELAKKCGITVSQVSNWFGNKRIRYKKNI	NP_002577		
	**Hs PBX3**	ARRKRRNFSKQATEILNEYFYSHLSNPYPSEEAKEELAKKCSITVSQVSNWFGNKRIRYKKNI	NP_006186		
	**Hs PBX4**	ARRKRRNFSKQATEVLNEYFYSHLNNPYPSEEAKEELARKGGLTISQVSNWFGNKRIRYKKNM	NP_079521		
	**Dm exd**	ARRKRRNFSKQASEILNEYFYSHLSNPYPSEEAKEELARKCGITVSQVSNWFGNKRIRYKKNI	NP_523360		
**Pknox**	**Hs PKNOX1**	SKNKRGVLPKHATNVMRSWLFQHIGHPYPTEDEKKQIAAQTNLTLLQVNNWFINARRRILQPM	NP_004562	97	94
	**Hs PKNOX2**	SKNKRGVLPKHATNIMRSWLFQHLMHPYPTEDEKRQIAAQTNLTLLQVNNWFINARRRILQPM	NP_071345		
	**Am Prep**	GRQKRGVLPKQATSIMRTWLFEHLVHPYPTEDEKRQIASQTNLTLLQVNNWFINARRRILQPM	XP_001120618		
**Tgif**	**Hs TGIF1**	KRRRRGNLPKESVQILRDWLYEHRYNAYPSEQEKALLSQQTHLSTLQVCNWFINARRRLLPDM	NP_733796	100	88
	**Hs TGIF2**	KRKRRGNLPKESVKILRDWLYLHRYNAYPSEQEKLSLSGQTNLSVLQICNWFINARRRLLPDM	NP_068581		
	**Hs TGIF2LX**	KKKRKGNLPAESVKILRDWMYKHRFKAYPSEEEKQMLSEKTNLSLLQISNWFINARRRILPDM	NP_620410		
	**Hs TGIF2LY**	KKKRKGNLPAESVKILRDWMYKHRFKAYPSEEEKQMLSEKTNLSLLRISNWFINARRRILPDM	NP_631960		
	**Dm achi**	LRKRRGNLPKTSVKILKRWLYEHRYNAYPSDAEKFTLSQEANLTVLQVCNWFINARRRILPEM	NP_725182		
	**Dm vis**	LRKRRGNLPKSSVKILKRWLYEHRYNAYPSDAEKFTLSQEANLTVLQVCNWFINARRRILPEM	NP_523714		

CUT class

```
                                _______I_______      _____II_____     _________III_________
                                10        20        30        40        50        60
                            ....|....|....|....|.. ..|....|....|....|....|....|....|....|
          Consensus         KRRKRTTFTKEQLLELEKEFAK-NPYPSREEREELAASLGLTERQVKVWFQNRRAKWKKQE     Accession      ML bs    NJ bs

Cux       Hs CUX1           LKKPRVVLAPEEKEALKRAYQQ-KPYPSPKTIEDLATQLNLKTSTVINWFHNYRSRIRREL     NP_853530      90       n/a
          Hs CUX2           IKKPRVVLAPEEKEALRKAYQL-EPYPSQQTIELLSFQLNLKTNTVINWFHNYRSRMRREM     NP_056082
          Dm ct             SKKQRVLFSEEQKEALRLAFAL-DPYPNVGTIEFLANELGLATRTITNWFHNHRMRLKQQV     NP_524764

Onecut    Hs ONECUT1        PKKPRLVFTDVQRRTLHAIFKE-NKRPSKELQITISQQLGLELSTVSNFFMNARRRSLDKW     NP_004489      99       96
          Hs ONECUT2        QKKSRLVFTDLQRRTLFAIFKE-NKRPSKEMQITISQQLGLELTTVSNFFMNARRRSLEKW     NP_004843
          Hs ONECUT3        PKKQRLVFTDLQRRTLIAIFKE-NKRPSKEMQVTISQQLGLELNTVSNFFMNARRRCMNRW     NP_001073957
          Dm onecut         PKKPRLVFTDLQRRTLQAIFKE-TKRPSKEMQVTIARQLGLEPTTVGNFFMNARRRSMDKW     NP_524842

Satb      Hs SATB1          KTRPRTKISVEALGILQSFIQDVGLYPDEEAIQTLSAQLDLPKYTIIKFFQNQRYYLKHHG     NP_002962      prot/cnid homolog not found
          Hs SATB2          KPRSRTKISLEALGILQSFIHDVGLYPDQEAIHTLSAQLDLPKHTIIKFFQNQRYHVKHHG     NP_056080
```

PROS class

```
                                _______I_______     _____II_____       _________III_________
                                10        20        30        40          50        60
                            ....|....|....|....|....|....|....|....|.   ...|....|....|....|
          Consensus         KRRKRTTFTKEQLLELEKEFAKNPYPSREEREELAASLGLT---ERQVKVWFQNRRAKWKKQE     Accession      ML bs    NJ bs

Prox      Hs PROX1          GSAMQEGLSPNHLKKAKLMFFYTRYPSSNMLKTYFSDVKFNRCITSQLIKWFSNFREFYYIQM     NP_002754      100      99
          Hs PROX2          LVHIQEGLNPGHLKKAKLMFFFTRYPSSNLLKVYFPDVQFNRCITSQMIKWFSNFREFYYIQM     NP_001073877
          Dm pros           MAPTSSTLTPMHLRKAKLMFFWVRYPSSAVLKMYFPDIKFNKNNTAQLVKWFSNFREFYYIQM     NP_731565
```

ZF class

```
                                _______I_______     _____II_____     _________III_________
                                10        20        30        40        50        60
                            ....|....|....|....|....|....|....|....|....|....|....|....|
          Consensus         KRRKRTTFTKEQLLELEKEFAKNPYPSREEREELAASLGLTERQVKVWFQNRRAKWKKQE     Accession      ML bs    NJ bs

Adnp      Hs ADNP           PKGHEDDSYEARKSFLTKYFNKQPYPTRREIEKLAASLWLWKSDIASHFSNKRKKCVRDC     NP_056154      prot/cnid homolog not found
          Hs ADNP2          PKKYEGRSYEEKKQFLKDYFHKKPYPSKKEIELLSSLFWVWKIDVASFFGKRRYICMKAI     NP_055728
```

```
                          ______I______              ____II____   _______III_______
                          10        20                 30        40        50        60
                      ....|....|....|....|....|..      ..|....|....|....|....|....|....|
        Consensus     KRRKRTTFTKEQLLELEKEFAKNPYPS-----------REEREELAASLGLTERQVKVWFQNRRAKWKKQE      Accession     ML bs     NJ bs

Tshz    Hs TSHZ1      RKGRQSNWNPQHLLILQAQFASSLRETTEGKYIMSDLGPQERVHISKFTGLSMTTISHWLANVKYQLRRTG      NP_005777     no homeodomain in Dm tsh
        Hs TSHZ2      RKGRQSNWNPQHLLILQAQFASSLFQTSEGKYLLSDLGPQERMQISKFTGLSMTTISHWLANVKYQLRKTG      NP_775756
        Hs TSHZ3      RKGRQSNWNPQHLLILQAQFAASLRQTSEGKYIMSDLSPQERMHISRFTGLSMTTISHWLANVKYQLRRTG      NP_065907
```

```
                           ______I______   ____II____   _______III_______
                           10        20        30        40        50        60
                      ....|.. ..|....|....|....|....|....|....|....|....|....|
        Consensus     KRRKRTT-FTKEQLLELEKEFAKNPYPSREEREELAASLGLTERQVKVWFQNRRAKWKKQE      Accession     ML bs     NJ bs

Zeb     Hs ZEB1       GNLSPSQPPLKNLLSLLKAYYALNAQPSAEELSKIADSVNLPLDVVKKWFEKMQAGQISVQ      NP_110378     n/a       n/a
        Hs ZEB2       GMTSPIN-PYKDHMSVLKAYYAMNMEPNSDELLKISIAVGLPQEFVKEWFEQRKVYQYSNS      NP_055610
        Dm zfh1       KVRVRTA-INEEQQQLKQHYSLNARPSRDEFRMIAARLQLDPRVVQVWFQNNRSRERKMQ      NP_733402
```

```
                          ______I______   ____II____   _______III_______
                          10        20        30        40        50        60
                      ....|....|....|....|....|....|....|....|....|....|....|....|
        Consensus     KRRKRTTFTKEQLLELEKEFAKNPYPSREEREELAASLGLTERQVKVWFQNRRAKWKKQE       Accession     ML bs     NJ bs

Zfhx    Hs ZFHX3-I    NKRPRTRITDDQLRVLRQYFDINNSPSEEQIKEMADKSGLPQKVIKHWFRNTLFKERQRN       NP_008816     99        98
        Hs ZFHX4-I    FKRPRTRITDDQLKILRAYFDINNSPSEEQIQEMAEKSGLSQKVIKHWFRNTLFKERQRN       NP_078997
        Dm zfh2-I     QKRARTRITDDQLKILRAHFDINNSPSEESIMEMSQKANLPMKVVKHWFRNTLFKERQRN       NP_524623

        Hs ZFHX2-I    RRFSRTKFTEFQTQALQSFFETSAYPKDGEVERLASLLGLASRVVVVWFQNARQKARKNA       NP_207646     97        92
        Hs ZFHX3-II   KRSSRTRFTDYQLRVLQDFFDANAYPKDDEFEQLSNLLNLPTRVIVVWFQNARQKARKNY       NP_008816
        Hs ZFHX4-II   KRSSRTRFTDYQLRVLQDFFDTNAYPKDDEIEQLSTVLNLPTRVIVVWFQNARQKARKSY       NP_078997
        Dm zfh2-II    KRANRTRFTDYQIKVLQEFFENNSYPKDSDLEYLSKLLLLSPRVIVVWFQNARQKQRKIY       NP_524623

        Hs ZFHX2-II   DKRLRTTILPEQLEILYRWYMQDSNPTRKMLDCISEEVGLKKRVVQVWFQNTRARERKGQ       NP_207646     91        95
        Hs ZFHX3-III  DKRLRTTITPEQLEILYQKYLLDSNPTRKMLDHIAHEVGLKKRVVQVWFQNTRARERKGQ       NP_008816
        Hs ZFHX4-III  DKRLRTTITPEQLEILYEKYLLDSNPTRKMLDHIAREVGLKKRVVQVWFQNTRARERKGQ       NP_078997
        Dm zfh2-III   NKRLRTTILPEQLNFLYECYQSESNPSRKMLEEISKKVNLKKRVVQVWFQNSRAKDKKSR       NP_524623

        Hs ZFHX2-III  QRRYRTQMSSLQLKIMKACYEAYRTPTMQECEVLGEEIGLPKRVIQVWFQNARAKEKKAK       NP_207646
        Hs ZFHX3-IV   QKRFRTQMTNLQLKVLKSCFNDYRTPTMLECEVLGNDIGLPKRVVQVWFQNARAKEKKSK       NP_008816
        Hs ZFHX4-IV   HKRFRTQMSNLQLKVLKACFSDYRTPTMQECEMLGNEIGLPKRVVQVWFQNARAKEKKFK       NP_078997
```

```
                          ______I______      ____II____    _______III_______
                          10        20        30        40        50        60
                ....|.. ..|....|....|....|....|....|....|....|....|....|....|

         Consensus      KRRKRTT-FTKEQLLELEKEFAKNPYPSREEREELAASLGLTERQVKVWFQNRRAKWKKQE       Accession      ML bs     NJ bs

Zhx/     Hs ZHX1-I      NSIPTYN-AALDNNPLLLNTYNKFPYPTMSEITVLSAQAKYTEEQIKIWFSAQRLKHGVSW       NP_001017926   prot/cnid homolog not found
Homez    Hs ZHX2-I      LNTTKYN-SALDTNATMINSFNKFPYPTQAELSWLTAASKHPEEHIRIWFATQRLKHGISW       NP_055758
         Hs ZHX3-I      SSIPTYN-AAMDSNSFLKNSFHKFPYPTKAELCYLTVVTKYPEEQLKIWFTAQRLKQGISW       NP_055850
         Hs HOMEZ-I     WTQAAQT-SELDSNEHLLKTFSYFPYPSLADIALLCLRYGLQMEKVKTWFMAQRLRCGISW       NP_065885

         Hs ZHX1-II     SFGIRAK-KTKEQLAELKVSYLKNQFPHDSEIIRLMKITGLTKGEIKKWFSDTRYNQRNSK       NP_001017926
         Hs ZHX2-II     TPASDRK-KTKEQIAHLKASFLQSQFPDDAEVYRLIEVTGLARSEIKKWFSDHRYRCQRGI       NP_055758
         Hs ZHX3-II     ASIYKNK-KSHEQLSALKGSFCRNQFPGQSEVEHLTKVTGLSTREVRKWFSDRRYHCRNLK       NP_055850

         Hs ZHX1-III    TPQKFKE-KTAEQLRVLQASFLNSSVLTDEELNRLRAQTKLTRREIDAWFTEKKKSKALKE       NP_001017926
         Hs ZHX2-III    APQKFKE-KTQGQVKILEDSFLKSSFPTQAELDRLRVETKLSRREIDSWFSERRKLRDSME       NP_055758
         Hs ZHX3-III    TPTKYKE-RAPEQLRALESSFAQNPLPLDEELDRLRSETKMTRREIDSWFSERRKKVNAEE       NP_055850

         Hs ZHX1-IV     STGKICK-KTPEQLHMLKSAFVRTQWPSPEEYDKLAKESGLARTDIVSWFGDTRYAWKNGN       NP_001017926
         Hs ZHX2-IV     SPSPAIA-KSQEQVHLLRSTFARTQWPTPQEYDQLAAKTGLVRTEIVRWFKENRCLLKTGT       NP_055758
         Hs ZHX3-IV     PGKVSCK-KTAQQRHLLRQLFVQTQWPSNQDYDSIMAQTGLPRPEVVRWFGDSRYALKNGQ       NP_055850
         Hs HOMEZ-II    RQRKTKR-KTKEQLAILKSFFLQCQWARREDYQKLEQITGLPRPEIIQWFGDTRYALKHGQ       NP_065885

         Hs ZHX1-V      DRGPSLI-KFKTGTAILKDYYLKHKFLNEQDLDELVNKSHMGYEQVREWFAERQRRSELGI       NP_001017926
         Hs ZHX3-V      FPPGLLV-IAPGNRELLQDYYMTHKMLYEEDLQNLCDKTQMSSQQVKQWFAEKMGEETRAV       NP_055850
         Hs HOMEZ-III   TPPLPIP-PPPPDIQPLERYWAAHQQLRETDIPQLSQASRLSTQQVLDWFDSRLPQPAEVV       NP_065885
```

CERS class

```
                         ______I______      ____II____    _______III_______
                         10        20        30        40        50        60
                ....|....|....|....|.. ..|....|....|....|....|....|....|....|

         Consensus      KRRKRTTFTKEQLLELEKEFAK-NPYPSREEREELAASLGLTERQVKVWFQNRRAKWKKQE       Accession      ML bs     NJ bs

Cers     Hs CERS2       NIKEKTRLRAPPNATLEHFYLTSGKQPKQVEVELLSRQSGLSGRQVERWFRRRRNQDRPSL       NP_859530      87        78
         Hs CERS3       GIKETV-RKVTPNTVLENFFKHSTRQPLQTDIYGLAKKCNLTERQVERWFRSRRNQERPSR       NP_849164
         Hs CERS4       GVRDQTRRQVKPNATLEKHFLTEGHRPKEPQLSLLAAQCGLTLQQTQRWFRRRRNQDRPQL       NP_078828
         Hs CERS5       GIEDSGPYQAQPNAILEKVFISITKYPDKKRLEGLSKQLDWNVRKIQCWFRHRRNQDKPPT       NP_671723
         Hs CERS6       NIQANGPQIAPPNAILEKVFTAITKHPDEKRLEGLSKQLDWDVRSIQRWFRQRRNQEKPST       NP_982288
         Dm Lag1        GIRSSRPKKAANVPILEKTYAKSTRLDKKK-LVPLSKQTDMSEREIERWWRLRRAQDKPST       NP_727075
```
