# Supplementary material for: Classification and nomenclature of all human homeobox genes
Source: BMC Biol. 2007 Oct 26;5:47. doi: 10.1186/1741-7007-5-47 (PMC2211742; doi:10.1186/1741-7007-5-47)
Supplement: Additional file 7 — Phylogenetic input file. All human and invertebrate homeodomains used in phylogenetic analyses are shown, after alignment and removal of insertions to give a uniform 60-amino-acid alignment. [file 1741-7007-5-47-S7.htm]

>HsCDX1


>HsCDX1

KDKYRVVYTDHQRLELEKEFHYSRYITIRRKSELAANLGLTERQVKIWFQNRRAKERKVN

>HsCDX2

KDKYRVVYTDHQRLELEKEFHYSRYITIRRKAELAATLGLSERQVKIWFQNRRAKERKIN

>HsCDX4

KEKYRVVYTDHQRLELEKEFHCNRYITIQRKSELAVNLGLSERQVKIWFQNRRAKERKMI

>HsEN1

DKRPRTAFTAEQLQRLKAEFQANRYITEQRRQTLAQELSLNESQIKIWFQNKRAKIKKAT

>HsEN2

DKRPRTAFTAEQLQRLKAEFQTNRYLTEQRRQSLAQELSLNESQIKIWFQNKRAKIKKAT

>HsEVX1

MRRYRTAFTREQIARLEKEFYRENYVSRPRRCELAAALNLPETTIKVWFQNRRMKDKRQR

>HsEVX2

VRRYRTAFTREQIARLEKEFYRENYVSRPRRCELAAALNLPETTIKVWFQNRRMKDKRQR

>HsGBX1

SRRRRTAFTSEQLLELEKEFHCKKYLSLTERSQIAHALKLSEVQVKIWFQNRRAKWKRIK

>HsGBX2

NRRRRTAFTSEQLLELEKEFHCKKYLSLTERSQIAHALKLSEVQVKIWFQNRRAKWKRVK

>HsGSX1

SKRMRTAFTSTQLLELEREFASNMYLSRLRRIEIATYLNLSEKQVKIWFQNRRVKHKKEG

>HsGSX2

GKRMRTAFTSTQLLELEREFSSNMYLSRLRRIEIATYLNLSEKQVKIWFQNRRVKHKKEG

>HsHOXA1

PNAVRTNFTTKQLTELEKEFHFNKYLTRARRVEIAASLQLNETQVKIWFQNRRMKQKKRE

>HsHOXB1

PSGLRTNFTTRQLTELEKEFHFNKYLSRARRVEIAATLELNETQVKIWFQNRRMKQKKRE

>HsHOXD1

SSAIRTNFSTKQLTELEKEFHFNKYLTRARRIEIANCLHLNDTQVKIWFQNRRMKQKKRE

>HsHOXA2

SRRLRTAYTNTQLLELEKEFHFNKYLCRPRRVEIAALLDLTERQVKVWFQNRRMKHKRQT

>HsHOXB2

ARRLRTAYTNTQLLELEKEFHFNKYLCRPRRVEIAALLDLTERQVKVWFQNRRMKHKRQT

>HsHOXA3

SKRARTAYTSAQLVELEKEFHFNRYLCRPRRVEMANLLNLTERQIKIWFQNRRMKYKKDQ

>HsHOXB3

SKRARTAYTSAQLVELEKEFHFNRYLCRPRRVEMANLLNLSERQIKIWFQNRRMKYKKDQ

>HsHOXD3

SKRVRTAYTSAQLVELEKEFHFNRYLCRPRRVEMANLLNLTERQIKIWFQNRRMKYKKDQ

>HsHOXA4

PKRSRTAYTRQQVLELEKEFHFNRYLTRRRRIEIAHTLCLSERQVKIWFQNRRMKWKKDH

>HsHOXB4

PKRSRTAYTRQQVLELEKEFHYNRYLTRRRRVEIAHALCLSERQIKIWFQNRRMKWKKDH

>HsHOXC4

PKRSRTAYTRQQVLELEKEFHYNRYLTRRRRIEIAHSLCLSERQIKIWFQNRRMKWKKDH

>HsHOXD4

PKRSRTAYTRQQVLELEKEFHFNRYLTRRRRIEIAHTLCLSERQIKIWFQNRRMKWKKDH

>HsHOXA5

GKRARTAYTRYQTLELEKEFHFNRYLTRRRRIEIAHALCLSERQIKIWFQNRRMKWKKDN

>HsHOXB5

GKRARTAYTRYQTLELEKEFHFNRYLTRRRRIEIAHALCLSERQIKIWFQNRRMKWKKDN

>HsHOXC5

GKRSRTSYTRYQTLELEKEFHFNRYLTRRRRIEIANNLCLNERQIKIWFQNRRMKWKKDS

>HsHOXA6

GRRGRQTYTRYQTLELEKEFHFNRYLTRRRRIEIANALCLTERQIKIWFQNRRMKWKKEN

>HsHOXB6

GRRGRQTYTRYQTLELEKEFHYNRYLTRRRRIEIAHALCLTERQIKIWFQNRRMKWKKES

>HsHOXC6

RRRGRQIYSRYQTLELEKEFHFNRYLTRRRRIEIANALCLTERQIKIWFQNRRMKWKKES

>HsHOXA7

RKRGRQTYTRYQTLELEKEFHFNRYLTRRRRIEIAHALCLTERQIKIWFQNRRMKWKKEH

>HsHOXB7

RKRGRQTYTRYQTLELEKEFHYNRYLTRRRRIEIAHTLCLTERQIKIWFQNRRMKWKKEN

>HsHOXB8

RRRGRQTYSRYQTLELEKEFLFNPYLTRKRRIEVSHALGLTERQVKIWFQNRRMKWKKEN

>HsHOXC8

RRSGRQTYSRYQTLELEKEFLFNPYLTRKRRIEVSHALGLTERQVKIWFQNRRMKWKKEN

>HsHOXD8

RRRGRQTYSRFQTLELEKEFLFNPYLTRKRRIEVSHALALTERQVKIWFQNRRMKWKKEN

>HsHOXA9

TRKKRCPYTKHQTLELEKEFLFNMYLTRDRRYEVARLLNLTERQVKIWFQNRRMKMKKIN

>HsHOXB9

SRKKRCPYTKYQTLELEKEFLFNMYLTRDRRHEVARLLNLSERQVKIWFQNRRMKMKKMN

>HsHOXC9

TRKKRCPYTKYQTLELEKEFLFNMYLTRDRRYEVARVLNLTERQVKIWFQNRRMKMKKMN

>HsHOXD9

TRKKRCPYTKYQTLELEKEFLFNMYLTRDRRYEVARILNLTERQVKIWFQNRRMKMKKMS

>HsHOXA10

GRKKRCPYTKHQTLELEKEFLFNMYLTRERRLEISRSVHLTDRQVKIWFQNRRMKLKKMN

>HsHOXC10

GRKKRCPYTKHQTLELEKEFLFNMYLTRERRLEISKTINLTDRQVKIWFQNRRMKLKKMN

>HsHOXD10

GRKKRCPYTKHQTLELEKEFLFNMYLTRERRLEISKSVNLTDRQVKIWFQNRRMKLKKMS

>HsHOXA11

TRKKRCPYTKYQIRELEREFFFSVYINKEKRLQLSRMLNLTDRQVKIWFQNRRMKEKKIN

>HsHOXC11

TRKKRCPYSKFQIRELEREFFFNVYINKEKRLQLSRMLNLTDRQVKIWFQNRRMKEKKLS

>HsHOXD11

SRKKRCPYTKYQIRELEREFFFNVYINKEKRLQLSRMLNLTDRQVKIWFQNRRMKEKKLN

>HsHOXC12

SRKKRKPYSKLQLAELEGEFLVNEFITRQRRRELSDRLNLSDQQVKIWFQNRRMKKKRLL

>HsHOXD12

ARKKRKPYTKQQIAELENEFLVNEFINRQKRKELSNRLNLSDQQVKIWFQNRRMKKKRVV

>HsHOXA13

GRKKRVPYTKVQLKELEREYATNKFITKDKRRRISATTNLSERQVTIWFQNRRVKEKKVI

>HsHOXB13

GRKKRIPYSKGQLRELEREYAANKFITKDKRRKISAATSLSERQITIWFQNRRVKEKKVL

>HsHOXC13

GRKKRVPYTKVQLKELEKEYAASKFITKEKRRRISATTNLSERQVTIWFQNRRVKEKKVV

>HsHOXD13

GRKKRVPYTKLQLKELENEYAINKFINKDKRRRISAATNLSERQVTIWFQNRRVKDKKIV

>HsMNX1

CRRPRTAFTSQQLLELEHQFKFNKYLSRPKRFEVATSLMLTETQVKIWFQNRRMKWKRSK

>HsMEOX1

ARKERTAFTKEQLRELEAEFAHHNYLTRLRRYEIAVNLDLSERQVKVWFQNRRMKWKRVK

>HsMEOX2

PRKERTAFTKEQIRELEAEFAHHNYLTRLRRYEIAVNLDLTERQVKVWFQNRRMKWKRVK

>HsPDX1

NKRTRTAYTRAQLLELEKEFLFNKYISRPRRVELAVMLNLTERHIKIWFQNRRMKWKKEE

>HsBARHL1

PRKARTAFTDHQLAQLERSFERQKYLSVQDRMELAASLNLTDTQVKTWYQNRRTKWKRQT

>HsBARHL2

PRKARTAFSDHQLNQLERSFERQKYLSVQDRMDLAAALNLTDTQVKTWYQNRRTKWKRQT

>HsBARX1�

GRRSRTVFTELQLMGLEKRFEKQKYLSTPDRIDLAESLGLSQLQVKTWYQNRRMKWKKIV

>HsBARX2

PRRSRTIFTELQLMGLEKKFQKQKYLSTPDRLDLAQSLGLTQLQVKTWYQNRRMKWKKMV

>HsBSX

RRKARTVFSDSQLSGLEKRFEIQRYLSTPERVELATALSLSETQVKTWFQNRRMKHKKQL

>HsDBX1

GMLRRAVFSDVQRKALEKMFQKQKYISKPDRKKLAAKLGLKDSQVKIWFQNRRMKWRNSK

>HsDBX2

GILRRAVFSEDQRKALEKMFQKQKYISKTDRKKLAINLGLKESQVKIWFQNRRMKWRNSK

>HsDLX1

IRKPRTIYSSLQLQALNRRFQQTQYLALPERAELAASLGLTQTQVKIWFQNKRSKFKKLM

>HsDLX2

VRKPRTIYSSFQLAALQRRFQKTQYLALPERAELAASLGLTQTQVKIWFQNRRSKFKKMW

>HsDLX3

VRKPRTIYSSYQLAALQRRFQKAQYLALPERAELAAQLGLTQTQVKIWFQNRRSKFKKLY

>HsDLX4

LRKPRTIYSSLQLQHLNQRFQHTQYLALPERAQLAAQLGLTQTQVKIWFQNKRSKYKKLL

>HsDLX5

VRKPRTIYSSFQLAALQRRFQKTQYLALPERAELAASLGLTQTQVKIWFQNKRSKIKKIM

>HsDLX6

IRKPRTIYSSLQLQALNHRFQQTQYLALPERAELAASLGLTQTQVKIWFQNKRSKFKKLL

>HsEMX1

PKRIRTAFSPSQLLRLERAFEKNHYVVGAERKQLAGSLSLSETQVKVWFQNRRTKYKRQK

>HsEMX2

PKRIRTAFSPSQLLRLEHAFEKNHYVVGAERKQLAHSLSLTETQVKVWFQNRRTKFKRQK

>HsHHEX

RKGGQVRFSNDQTIELEKKFETQKYLSPPERKRLAKMLQLSERQVKTWFQNRRAKWRRLK

>HsHLX

RSWSRAVFSNLQRKGLEKRFEIQKYVTKPDRKQLAAMLGLTDAQVKVWFQNRRMKWRHSK

>HsLBX1

RRKSRTAFTNHQIYELEKRFLYQKYLSPADRDQIAQQLGLTNAQVITWFQNRRAKLKREL

>HsLBX2

RRKSRTAFTAQQVLELERRFVFQKYLAPSERDGLATRLGLANAQVVTWFQNRRAKLKRDV

>HsMSX1

NRKPRTPFTTAQLLALERKFRQKQYLSIAERAEFSSSLSLTETQVKIWFQNRRAKAKRLQ

>HsMSX2

NRKPRTPFTTSQLLALERKFRQKQYLSIAERAEFSSSLNLTETQVKIWFQNRRAKAKRLQ

>HsNANOG

KQKTRTVFSSTQLCVLNDRFQRQKYLSLQQMQELSNILNLSYKQVKTWFQNQRMKSKRWQ

>HsNKX1.1

PRRARTAFTYEQLVALENKFKATRYLSVCERLNLALSLSLTETQVKIWFQNRRTKWKKQN

>HsNKX1.2

PRRARTAFTYEQLVALENKFRATRYLSVCERLNLALSLSLTETQVKIWFQNRRTKWKKQN

>HsNKX2.1

RRKRRVLFSQAQVYELERRFKQQKYLSAPEREHLASMIHLTPTQVKIWFQNHRYKMKRQA

>HsNKX2.4

RRKRRVLFSQAQVYELERRFKQQKYLSAPEREHLASMIHLTPTQVKIWFQNHRYKMKRQA

>HsNKX2.2

KRKRRVLFSKAQTYELERRFRQQRYLSAPEREHLASLIRLTPTQVKIWFQNHRYKMKRAR

>HsNKX2.8

RKKRRVLFSKAQTLELERRFRQQRYLSAPEREQLASLLRLTPTQVKIWFQNHRYKLKRAR

>HsNKX3.1

QKRSRAAFSHTQVIELERKFSHQKYLSAPERAHLAKNLKLTETQVKIWFQNRRYKTKRKQ

>HsNKX3.2

KKRSRAAFSHAQVFELERRFNHQRYLSGPERADLAASLKLTETQVKIWFQNRRYKTKRRQ

>HsNKX2.3

RRKPRVLFSQAQVFELERRFKQQRYLSAPEREHLASSLKLTSTQVKIWFQNRRYKCKRQR

>HsNKX2.5

RRKPRVLFSQAQVYELERRFKQQRYLSAPERDQLASVLKLTSTQVKIWFQNRRYKCKRQR

>HsNKX2.6

RRKPRVLFSQAQVLALERRFKQQRYLSAPEREHLASALQLTSTQVKIWFQNRRYKCKRQR

>HsHMX1

KKKTRTVFSRSQVFQLESTFDLKRYLSTAERAGLAASLQLTETQVKIWFQNRRNKWKRHV

>HsHMX2

KKKTRTVFSRSQVYQLESTFDMKRYLSSSERACLASSLQLTETQVKTWFQNRRNKWKRQL

>HsHMX3

KKKTRTVFSRSQVFQLESTFDMKRYLSSSERAGLAASLHLTETQVKIWFQNRRNKWKRQL

>HsNKX6.1

RKHTRPTFSGQQIFALEKTFEQTKYLAGPERARLAYSLGMTESQVKVWFQNRRTKWRKKH

>HsNKX6.2

KKHSRPTFSGQQIFALEKTFEQTKYLAGPERARLAYSLGMTESQVKVWFQNRRTKWRKRH

>HsNKX6.3

KKHTRPTFTGHQIFALEKTFEQTKYLAGPERARLAYSLGMTESQVKVWFQNRRTKWRKKS

>HsNOTO

QKRVRTMFNLEQLEELEKVFAKQHNLVGKKRAQLAARLKLTENQVRVWFQNRRVKYQKQQ

>HsTLX1

KKKPRTSFTRLQICELEKRFHRQKYLASAERAALAKALKMTDAQVKTWFQNRRTKWRRQT

>HsTLX2

RKKPRTSFSRSQVLELERRFLRQKYLASAERAALAKALRMTDAQVKTWFQNRRTKWRRQT

>HsTLX3

RKKPRTSFSRVQICELEKRFHRQKYLASAERAALAKSLKMTDAQVKTWFQNRRTKWRRQT

>HsVAX1

PKRTRTSFTAEQLYRLEMEFQRCQYVVGRERTELARQLNLSETQVKVWFQNRRTKQKKDQ

>HsVAX2

PKRTRTSFTAEQLYRLEMEFQRCQYVVGRERTELARQLNLSETQVKVWFQNRRTKQKKDQ

>HsVENTX

APRVRTAFTMEQVRTLEGVFQHHQYLSPLERKRLAREMQLSEVQIKTWFQNRRMKHKRQM

>HsALX1

KRRHRTTFTSLQLEELEKVFQKTHYPDVYVREQLALRTELTEARVQVWFQNRRAKWRKRE

>HsALX3

KRRNRTTFSTFQLEELEKVFQKTHYPDVYAREQLALRTDLTEARVQVWFQNRRAKWRKRE

>HsALX4

KRRNRTTFTSYQLEELEKVFQKTHYPDVYAREQLAMRTDLTEARVQVWFQNRRAKWRKRE

>HsARGFX

RHKERTSFTHQQYEELEALFSQTMFPDRNLQEKLALRLDLPESTVKVWFRNRRFKLKKQQ

>HsARX

QRRYRTTFTSYQLEELERAFQKTHYPDVFTREELAMRLDLTEARVQVWFQNRRAKWRKRE

>HsDMBX1

QRRSRTAFTAQQLEALEKTFQKTHYPDVVMRERLAMCTNLPEARVQVWFKNRRAKFRKKQ

>HsDPRX

SHRKRTMFTKKQLEDLNILFNENPYPNPSLQKEMASKIDIHPTVLQVWFKNHRAKLKKAK

>HsDRGX

QRRNRTTFTLQQLEALEAVFAQTHYPDVFTREELAMKINLTEARVQVWFQNRRAKWRKTE

>HsDUXAI

HRRCRTKFTEEQLKILINTFNQKPYPGYATKQKLALEINTEESRIQIWFQNRRARHGFQK

>HsDUXAII

ARRCRTTYSASQLHTLIKAFMKNPYPGIDSREELAKEIGVPESRVQIWFQNRRSRLLLQR

>HsDUXBI

FWRNRIQYNQSQKDILQSWFQHDPFPDKAAREQLAKEIGVPESNIQVWFKNYRVKQRKLD

>HsDUXBII

ARQKQTFITWTQKNRLVQAFERNPFPDIATRKKLAEQTGLQESRIQMWFQKQRSLYLKKS

>HsESX1

KRRRRTAFTQFQLQELENFFDESQYPDVVARERLAARLNLTEDRVQVWFQNRRAKWKRNQ

>HsGSC

KRRHRTIFTDEQLEALENLFQETKYPDVGTREQLARKVHLREEKVEVWFKNRRAKWRRQK

>HsGSC2

TRRHRTIFSEEQLQALEALFVQNQYPDVSTRERLAGRIRLREERVEVWFKNRRAKWRHQK

>HsHESX1

GRRPRTAFTQNQIEVLENVFRVNCYPGIDIREDLAQKLNLEEDRIQIWFQNRRAKLKRSH

>HsHOPX

SAETASGPTEDQVEILEYNFNKVKHPDSTTLCLIAAEAGLSEEETQKWFKQRLAKWRRSE

>HsISX

KRRVRTTFTTEQLHELEKIFHFTHYPDVHIRSQLAARINLPEARVQIWFQNQRAKWRKQE

>HsMIXL

QRRKRTSFSAEQLQLLELVFRRTRYPDIHLRERLAALTLLPESRIQVWFQNRRAKSRRQS

>HsNOBOX

RKKTRTLYRSDQLEELEKIFQEDHYPDSDKRREIAQTVGVTPQRIMVWFQNRRAKWRKME

>HsOTP

QKRHRTRFTPAQLNELERSFAKTHYPDIFMREELALRIGLTESRVQVWFQNRRAKWKKRK

>HsOTX1

QRRERTTFTRSQLDVLEALFAKTRYPDIFMREEVALKINLPESRVQVWFKNRRAKCRQQQ

>HsOTX2

QRRERTTFTRAQLDVLEALFAKTRYPDIFMREEVALKINLPESRVQVWFKNRRAKCRQQQ

>HsCRX

QRRERTTFTRSQLEELEALFAKTQYPDVYAREEVALKINLPESRVQVWFKNRRAKCRQQR

>HsPAX3

QRRSRTTFTAEQLEELERAFERTHYPDIYTREELAQRAKLTEARVQVWFSNRRARWRKQA

>HsPAX7

QRRSRTTFTAEQLEELEKAFERTHYPDIYTREELAQRTKLTEARVQVWFSNRRARWRKQA

>HsPAX4

GHRNRTIFSPSQAEALEKEFQRGQYPDSVARGKLATATSLPEDTVRVWFSNRRAKWRRQE

>HsPAX6

LQRNRTSFTQEQIEALEKEFERTHYPDVFARERLAAKIDLPEARIQVWFSNRRAKWRREE

>HsPHOX2A

QRRIRTTFTSAQLKELERVFAETHYPDIYTREELALKIDLTEARVQVWFQNRRAKFRKQE

>HsPHOX2B

QRRIRTTFTSAQLKELERVFAETHYPDIYTREELALKIDLTEARVQVWFQNRRAKFRKQE

>HsPITX1

QRRQRTHFTSQQLQELEATFQRNRYPDMSMREEIAVWTNLTEPRVRVWFKNRRAKWRKRE

>HsPITX2

QRRQRTHFTSQQLQELEATFQRNRYPDMSTREEIAVWTNLTEARVRVWFKNRRAKWRKRE

>HsPITX3

QRRQRTHFTSQQLQELEATFQRNRYPDMSTREEIAVWTNLTEARVRVWFKNRRAKWRKRE

>HsPROP1

RRRHRTTFSPVQLEQLESAFGRNQYPDIWARESLARDTGLSEARIQVWFQNRRAKQRKQE

>HsPRRX1

QRRNRTTFNSSQLQALERVFERTHYPDAFVREDLARRVNLTEARVQVWFQNRRAKFRRNE

>HsPRRX2

QRRNRTTFNSSQLQALERVFERTHYPDAFVREELARRVNLSEARVQVWFQNRRAKFRRNE

>HsRAX

HRRNRTTFTTYQLHELERAFEKSHYPDVYSREELAGKVNLPEVRVQVWFQNRRAKWRRQE

>HsRAX2

HRRNRTTFTTYQLHQLERAFEASHYPDVYSREELAAKVHLPEVRVQVWFQNRRAKWRRQE

>HsRHOXF1

PRTRRTKFTLLQVEELESVFRHTQYPDVPTRRELAENLGVTEDKVRVWFKNKRARCRRHQ

>HsRHOXF2

QQPNVHAFTPLQLQELERIFQREQFPSEFLRRRLARSMNVTELAVQIWFENRRAKWRRHQ

>HsRHOXF2B

QQPNVHAFTPLQLQELECIFQREQFPSEFLRRRLARSMNVTELAVQIWFENRRAKWRRHQ

>HsSEBOX

HRRKRTTFSKGQLLELERAFAAWPYPNISTHEHLAWVTCLPEAKVQVWFQKRWAKIIKNR

>HsSHOX

QRRSRTNFTLEQLNELERLFDETHYPDAFMREELSQRLGLSEARVQVWFQNRRAKCRKQE

>HsSHOX2

QRRSRTNFTLEQLNELERLFDETHYPDAFMREELSQRLGLSEARVQVWFQNRRAKCRKQE

>HsTPRX1

QRQERTVYTESQQKVLEFYFQKDQYPNYDQRLNLAEMLSLREQQLQVWFKNRRAKLARER

>HsTPRXL

QRQDRTIYNWKQQEVLENHFKEEQYPDYDTRQELAEMLNLREYQVQVWFKNRRAKRSRER

>HsUNCX

RRRTRTNFTGWQLEELEKAFNESHYPDVFMREALALRLDLVESRVQVWFQNRRAKWRKKE

>HsVSX1

KRRHRTVFTAHQLEELEKAFSEAHYPDVYAREMLAVKTELPEDRIQVWFQNRRAKWRKRE

>HsVSX2

KRRHRTIFTSYQLEELEKAFNEAHYPDVYAREMLAMKTELPEDRIQVWFQNRRAKWRKRE

>HsLEUTX

YRRPRTRFLSKQLTALRELLEKTMHPSLATMGKLASKLQLDLSVVKIWFKNQRAKWKRQQ

>HsISL1

TTRVRTVLNEKQLHTLRTCYAANPRPDALMKEQLVEMTGLSPRVIRVWFQNKRCKDKKRS

>HsISL2

TTRVRTVLNEKQLHTLRTCYAANPRPDALMKEQLVEMTGLSPRVIRVWFQNKRCKDKKKS

>HsLHX1

RRGPRTTIKAKQLETLKAAFAATPKPTRHIREQLAQETGLNMRVIQVWFQNRRSKERRMK

>HsLHX5

RRGPRTTIKAKQLETLKAAFAATPKPTRHIREQLAQETGLNMRVIQVWFQNRRSKERRMK

>HsLHX2

TKRMRTSFKHHQLRTMKSYFAINHNPDAKDLKQLAQKTGLTKRVLQVWFQNARAKFRRNL

>HsLHX9

TKRMRTSFKHHQLRTMKSYFAINHNPDAKDLKQLAQKTGLTKRVLQVWFQNARAKFRRNL

>HsLHX3

AKRPRTTITAKQLETLKSAYNTSPKPARHVREQLSSETGLDMRVVQVWFQNRRAKEKRLK

>HsLHX4

AKRPRTTITAKQLETLKNAYKNSPKPARHVREQLSSETGLDMRVVQVWFQNRRAKEKRLK

>HsLHX6

AKRARTSFTAEQLQVMQAQFAQDNNPDAQTLQKLADMTGLSRRVIQVWFQNCRARHKKHT

>HsLHX8

AKRARTSFTADQLQVMQAQFAQDNNPDAQTLQKLAERTGLSRRVIQVWFQNCRARHKKHV

>HsLMX1A

PKRPRTILTTQQRRAFKASFEVSSKPCRKVRETLAAETGLSVRVVQVWFQNQRAKMKKLA

>HsLMX1B

PKRPRTILTTQQRRAFKASFEVSSKPCRKVRETLAAETGLSVRVVQVWFQNQRAKMKKLA

>HsHDX

SRKRRTQFSDRDLATLKKYWDNGMTVCREKIEAVATELNVDCEIVRTWIGNRRRKYRLMG

>HsPOU1F1

KRKRRTTISIAAKDALERHFGEQNKPSSQEIMRMAEELNLEKEVVRVWFCNRRQREKRVK

>HsPOU2F1

RRKKRTSIETNIRVALEKSFLENQKPTSEEITMIADQLNMEKEVIRVWFCNRRQKEKRIN

>HsPOU2F2

RRKKRTSIETNVRFALEKSFLANQKPTSEEILLIAEQLHMEKEVIRVWFCNRRQKEKRIN

>HsPOU2F3

KRKKRTSIETNIRLTLEKRFQDNPKPSSEEISMIAEQLSMEKEVVRVWFCNRRQKEKRIN

>HsPOU3F1

KRKKRTSIEVGVKGALESHFLKCPKPSAHEITGLADSLQLEKEVVRVWFCNRRQKEKRMT

>HsPOU3F2

KRKKRTSIEVSVKGALESHFLKCPKPSAQEITSLADSLQLEKEVVRVWFCNRRQKEKRMT

>HsPOU3F3

KRKKRTSIEVSVKGALESHFLKCPKPSAQEITNLADSLQLEKEVVRVWFCNRRQKEKRMT

>HsPOU3F4

KRKKRTSIEVSVKGVLETHFLKCPKPAAQEISSLADSLQLEKEVVRVWFCNRRQKEKRMT

>HsPOU4F1

KKRKRTSIAAPEKRSLEAYFAVQPRPSSEKIAAIAEKLDLKKNVVRVWFCNQRQKQKRMK

>HsPOU4F2

KKRKRTSIAAPEKRSLEAYFAIQPRPSSEKIAAIAEKLDLKKNVVRVWFCNQRQKQKRMK

>HsPOU4F3

RKRKRTSIAAPEKRSLEAYFAIQPRPSSEKIAAIAEKLDLKKNVVRVWFCNQRQKQKRMK

>HsPOU5F1

RKRKRTSIENRVRGNLENLFLQCPKPTLQQISHIAQQLGLEKDVVRVWFCNRRQKGKRSS

>HsPOU5F2

GKWRRASRERRIGNSLEKFFQRCPKPTPQQISHIAGCLQLQKDVVRVWFYNRSKMGSRPT

>HsPOU6F1

KRKRRTSFTPQAIEALNAYFEKNPLPTGQEITEIAKELNYDREVVRVWFCNRRQTLKNTS

>HsPOU6F2

KRKRRTSFTPQALEILNAHFEKNTHPSGQEMTEIAEKLNYDREVVRVWFCNKRQALKNTI

>HsHMBOX1

RRGSRFTWRKECLAVMESYFNENQYPDEAKREEIANACNVTSLKVYNWFANRRKEIKRRA

>HsHNF1A

GRRNRFKWGPASQQILFQAYERQKNPSKEERETLVEECNVTEVRVYNWFANRRKEEAFRH

>HsHNF1B

MRRNRFKWGPASQQILYQAYDRQKNPSKEEREALVEECNVTEVRVYNWFANRRKEEAFRQ

>HsSIX1

GEETSYCFKEKSRGVLREWYAHNPYPSPREKRELAEATGLTTTQVSNWFKNRRQRDRAAE

>HsSIX2

GEETSYCFKEKSRSVLREWYAHNPYPSPREKRELTEATGLTTTQVSNWFKNRRQRDRAAE

>HsSIX3

GEQKTHCFKERTRSLLREWYLQDPYPNPSKKRELAQATGLTPTQVGNWFKNRRQRDRAAA

>HsSIX6

GEQKTHCFKERTRNLLREWYLQDPYPNPSKKRELAQATGLTPTQVGNWFKNRRQRDRAAA

>HsSIX4

GEETVYCFKEKSRNALKELYKQNRYPSPAEKRHLAKITGLSLTQVSNWFKNRRQRDRNPS

>HsSIX5

GEETVYCFKERSRAALKACYRGNRYPTPDEKRRLATLTGLSLTQVSNWFKNRRQRDRTGA

>HsIRX1

DPGRPKNATRESTSTLKAWLNEHPYPTKGEKIMLAIITKMTLTQVSTWFANARRRLKKEN

>HsIRX2

DPAYRKNATRDATATLKAWLNEHPYPTKGEKIMLAIITKMTLTQVSTWFANARRRLKKEN

>HsIRX3

DPSRPKNATRESTSTLKAWLNEHPYPTKGEKIMLAIITKMTLTQVSTWFANARRRLKKEN

>HsIRX4

SGTRRKNATRETTSTLKAWLQEHPYPTKGEKIMLAIITKMTLTQVSTWFANARRRLKKEN

>HsIRX5

DPAYRKNATRDATATLKAWLNEHPYPTKGEKIMLAIITKMTLTQVSTWFANARRRLKKEN

>HsIRX6

GAGRRKNATRETTSTLKAWLNEHPYPTKGEKIMLAIITKMTLTQVSTWFANARRRLKKEN

>HsMEIS1

RHKKRGIFPKVATNIMRAWLFQHPYPSEEQKKQLAQDTGLTILQVNNWFINARRRIVQPM

>HsMEIS2

RQKKRGIFPKVATNIMRAWLFQHPYPSEEQKKQLAQDTGLTILQVNNWFINARRRIVQPM

>HsMEIS3

RNKKRGIFPKVATNIMRAWLFQHPYPSEEQKKQLAQDTGLTILQVNNWFINARRRIVQPM

>HsMKX

KVRHKRQALQDMARPLKQWLYKHPYPTKTEKILLALGSQMTLVQVSNWFANARRRLKNTV

>HsPBX1

ARRKRRNFNKQATEILNEYFYSHPYPSEEAKEELAKKCGITVSQVSNWFGNKRIRYKKNI

>HsPBX2

ARRKRRNFSKQATEVLNEYFYSHPYPSEEAKEELAKKCGITVSQVSNWFGNKRIRYKKNI

>HsPBX3

ARRKRRNFSKQATEILNEYFYSHPYPSEEAKEELAKKCSITVSQVSNWFGNKRIRYKKNI

>HsPBX4

ARRKRRNFSKQATEVLNEYFYSHPYPSEEAKEELARKGGLTISQVSNWFGNKRIRYKKNM

>HsPKNOX1

SKNKRGVLPKHATNVMRSWLFQHPYPTEDEKKQIAAQTNLTLLQVNNWFINARRRILQPM

>HsPKNOX2

SKNKRGVLPKHATNIMRSWLFQHPYPTEDEKRQIAAQTNLTLLQVNNWFINARRRILQPM

>HsTGIF1

KRRRRGNLPKESVQILRDWLYEHAYPSEQEKALLSQQTHLSTLQVCNWFINARRRLLPDM

>HsTGIF2

KRKRRGNLPKESVKILRDWLYLHAYPSEQEKLSLSGQTNLSVLQICNWFINARRRLLPDM

>HsTGIF2LX

KKKRKGNLPAESVKILRDWMYKHAYPSEEEKQMLSEKTNLSLLQISNWFINARRRILPDM

>HsTGIF2LY

KKKRKGNLPAESVKILRDWMYKHAYPSEEEKQMLSEKTNLSLLRISNWFINARRRILPDM

>HsCUX1

LKKPRVVLAPEEKEALKRAYQQKPYPSPKTIEDLATQLNLKTSTVINWFHNYRSRIRREL

>HsCUX2

IKKPRVVLAPEEKEALRKAYQLEPYPSQQTIELLSFQLNLKTNTVINWFHNYRSRMRREM

>HsONECUT1

PKKPRLVFTDVQRRTLHAIFKENKRPSKELQITISQQLGLELSTVSNFFMNARRRSLDKW

>HsONECUT2

QKKSRLVFTDLQRRTLFAIFKENKRPSKEMQITISQQLGLELTTVSNFFMNARRRSLEKW

>HsONECUT3

PKKQRLVFTDLQRRTLIAIFKENKRPSKEMQVTISQQLGLELNTVSNFFMNARRRCMNRW

>HsSATB1

KTRPRTKISVEALGILQSFIQDGLYPDEEAIQTLSAQLDLPKYTIIKFFQNQRYYLKHHG

>HsSATB2

KPRSRTKISLEALGILQSFIHDGLYPDQEAIHTLSAQLDLPKHTIIKFFQNQRYHVKHHG

>HsPROX1

GSAMQEGLSPNHLKKAKLMFFYTRYPSSNMLKTYFSDVKFNTSQLIKWFSNFREFYYIQM

>HsPROX2

LVHIQEGLNPGHLKKAKLMFFFTRYPSSNLLKVYFPDVQFNTSQMIKWFSNFREFYYIQM

>HsADNP

PKGHEDDSYEARKSFLTKYFNKQPYPTRREIEKLAASLWLWKSDIASHFSNKRKKCVRDC

>HsADNP2

PKKYEGRSYEEKKQFLKDYFHKKPYPSKKEIELLSSLFWVWKIDVASFFGKRRYICMKAI

>HsTSHZ1

RKGRQSNWNPQHLLILQAQFASSLRETPQERVHISKFTGLSMTTISHWLANVKYQLRRTG

>HsTSHZ2

RKGRQSNWNPQHLLILQAQFASSLFQTPQERMQISKFTGLSMTTISHWLANVKYQLRKTG

>HsTSHZ3

RKGRQSNWNPQHLLILQAQFAASLRQTPQERMHISRFTGLSMTTISHWLANVKYQLRRTG

>HsZEB1

GNLSPSQPLKNLLSLLKAYYALNAQPSAEELSKIADSVNLPLDVVKKWFEKMQAGQISVQ

>HsZEB2

GMTSPINPYKDHMSVLKAYYAMNMEPNSDELLKISIAVGLPQEFVKEWFEQRKVYQYSNS

>HsZFHX2I

RRFSRTKFTEFQTQALQSFFETSAYPKDGEVERLASLLGLASRVVVVWFQNARQKARKNA

>HsZFHX2II

DKRLRTTILPEQLEILYRWYMQDSNPTRKMLDCISEEVGLKKRVVQVWFQNTRARERKGQ

>HsZFHX2III

QRRYRTQMSSLQLKIMKACYEAYRTPTMQECEVLGEEIGLPKRVIQVWFQNARAKEKKAK

>HsZFHX3I

NKRPRTRITDDQLRVLRQYFDINNSPSEEQIKEMADKSGLPQKVIKHWFRNTLFKERQRN

>HsZFHX3II

KRSSRTRFTDYQLRVLQDFFDANAYPKDDEFEQLSNLLNLPTRVIVVWFQNARQKARKNY

>HsZFHX3III

DKRLRTTITPEQLEILYQKYLLDSNPTRKMLDHIAHEVGLKKRVVQVWFQNTRARERKGQ

>HsZFHX3IV

QKRFRTQMTNLQLKVLKSCFNDYRTPTMLECEVLGNDIGLPKRVVQVWFQNARAKEKKSK

>HsZFHX4I

FKRPRTRITDDQLKILRAYFDINNSPSEEQIQEMAEKSGLSQKVIKHWFRNTLFKERQRN

>HsZFHX4II

KRSSRTRFTDYQLRVLQDFFDTNAYPKDDEIEQLSTVLNLPTRVIVVWFQNARQKARKSY

>HsZFHX4III

DKRLRTTITPEQLEILYEKYLLDSNPTRKMLDHIAREVGLKKRVVQVWFQNTRARERKGQ

>HsZFHX4IV

HKRFRTQMSNLQLKVLKACFSDYRTPTMQECEMLGNEIGLPKRVVQVWFQNARAKEKKFK

>HsZHX1I

NSIPTYNAALDNNPLLLNTYNKFPYPTMSEITVLSAQAKYTEEQIKIWFSAQRLKHGVSW

>HsZHX1II

SFGIRAKKTKEQLAELKVSYLKNQFPHDSEIIRLMKITGLTKGEIKKWFSDTRYNQRNSK

>HsZHX1III

TPQKFKEKTAEQLRVLQASFLNSSVLTDEELNRLRAQTKLTRREIDAWFTEKKKSKALKE

>HsZHX1IV

STGKICKKTPEQLHMLKSAFVRTQWPSPEEYDKLAKESGLARTDIVSWFGDTRYAWKNGN

>HsZHX1V

DRGPSLIKFKTGTAILKDYYLKHKFLNEQDLDELVNKSHMGYEQVREWFAERQRRSELGI

>HsZHX2I

LNTTKYNSALDTNATMINSFNKFPYPTQAELSWLTAASKHPEEHIRIWFATQRLKHGISW

>HsZHX2II

TPASDRKKTKEQIAHLKASFLQSQFPDDAEVYRLIEVTGLARSEIKKWFSDHRYRCQRGI

>HsZHX2III

APQKFKEKTQGQVKILEDSFLKSSFPTQAELDRLRVETKLSRREIDSWFSERRKLRDSME

>HsZHX2IV

SPSPAIAKSQEQVHLLRSTFARTQWPTPQEYDQLAAKTGLVRTEIVRWFKENRCLLKTGT

>HsZHX3I

SSIPTYNAAMDSNSFLKNSFHKFPYPTKAELCYLTVVTKYPEEQLKIWFTAQRLKQGISW

>HsZHX3II

ASIYKNKKSHEQLSALKGSFCRNQFPGQSEVEHLTKVTGLSTREVRKWFSDRRYHCRNLK

>HsZHX3III

TPTKYKERAPEQLRALESSFAQNPLPLDEELDRLRSETKMTRREIDSWFSERRKKVNAEE

>HsZHX3IV

PGKVSCKKTAQQRHLLRQLFVQTQWPSNQDYDSIMAQTGLPRPEVVRWFGDSRYALKNGQ

>HsZHX3V

FPPGLLVIAPGNRELLQDYYMTHKMLYEEDLQNLCDKTQMSSQQVKQWFAEKMGEETRAV

>HsHOMEZI

WTQAAQTSELDSNEHLLKTFSYFPYPSLADIALLCLRYGLQMEKVKTWFMAQRLRCGISW

>HsHOMEZII

RQRKTKRKTKEQLAILKSFFLQCQWARREDYQKLEQITGLPRPEIIQWFGDTRYALKHGQ

>HsHOMEZIII

TPPLPIPPPPPDIQPLERYWAAHQQLRETDIPQLSQASRLSTQQVLDWFDSRLPQPAEVV

>HsCERS2

NIKEKTRLRAPPNATLEHFYLTGKQPKQVEVELLSRQSGLSGRQVERWFRRRRNQDRPSL

>HsCERS3

GIKETVRRKVTPNTVLENFFKHTRQPLQTDIYGLAKKCNLTERQVERWFRSRRNQERPSR

>HsCERS4

GVRDQTRRQVKPNATLEKHFLTGHRPKEPQLSLLAAQCGLTLQQTQRWFRRRRNQDRPQL

>HsCERS5

GIEDSGPYQAQPNAILEKVFISTKYPDKKRLEGLSKQLDWNVRKIQCWFRHRRNQDKPPT

>HsCERS6

NIQANGPQIAPPNAILEKVFTATKHPDEKRLEGLSKQLDWDVRSIQRWFRQRRNQEKPST

>DmAbdA

RRRGRQTYTRFQTLELEKEFHFNHYLTRRRRIEIAHALCLTERQIKIWFQNRRMKLKKEL

>DmAbdB

VRKKRKPYSKFQTLELEKEFLFNAYVSKQKRWELARNLQLTERQVKIWFQNRRMKNKKNS

>Dmachi

LRKRRGNLPKTSVKILKRWLYENAYPSDAEKFTLSQEANLTVLQVCNWFINARRRILPEM

>Dmacj6

KKRKRTSIAAPEKRSLEAYFAVQPRPSGEKIAAIAEKLDLKKNVVRVWFCNQRQKQKRIV

>Dmal

QRRYRTTFTSFQLEELEKAFSRTHYPDVFTREELAMKIGLTEARIQVWFQNRRAKWRKQE

>DmAntp

RKRGRQTYTRYQTLELEKEFHFNRYLTRRRRIEIAHALCLTERQIKIWFQNRRMKWKKEN

>Dmap

TKRMRTSFKHHQLRTMKSYFAINHNPDAKDLKQLSQKTGLPKRVLQVWFQNARAKWRRMM

>Dmara

LAARRKNATRESTATLKAWLNENPYPTKGEKIMLAIITKMTLTQVSTWFANARRRLKKEN

>DmAwh

TKRVRTTFTEEQLQVLQANFQIDSNPDGQDLERIASVTGLSKRVTQVWFQNSRARQKKHI

>DmBH1

QRKARTAFTDHQLQTLEKSFERQKYLSVQERQELAHKLDLSDCQVKTWYQNRRTKWKRQT

>DmBH2

QRKARTAFTDHQLQTLEKSFERQKYLSVQDRMELANKLELSDCQVKTWYQNRRTKWKRQT

>Dmbap

KKRSRAAFSHAQVFELERRFAQQRYLSGPERSEMAKSLRLTETQVKIWFQNRRYKTKRKQ

>Dmbsh

RRKARTVFSDPQLSGLEKRFEGQRYLSTPERVELATALGLSETQVKTWFQNRRMKHKKQL

>Dmbtn

NRKERTAFSKTQLKQLEAEFCYSNYLTRLRRYEIAVALELTERQVKVWFQNRRMKCKRIK

>DmC15

RKKPRTSFTRIQVAELEKRFHKQKYLASAERAALARGLKMTDAQVKTWFQNRRTKWRRQT

>Dmcad

KDKYRVVYTDFQRLELEKEYCTSRYITIRRKSELAQTLSLSERQVKIWFQNRRAKERKQN

>Dmcaup

LAARRKNATRESTATLKAWLSENPYPTKGEKIMLAIITKMTLTQVSTWFANARRRLKKEN

>DmCG11617

SRATKRLFTPDIKRMLKDWLIRNPYPSREEKKQLAAETGLTYTQICNWFANWRRKLKNSE

>DmCG12361

GMMRRAVFSDSQRKGLEKRFQQQKYISKPDRKKLAERLGLKDSQVKIWFQNRRMKWRNSK

>DmCG32105

PKRPRTILTSQQRKQFKASFDQSPKPCRKVREALAKDTGLSVRVVQVWFQNQRAKMKKIQ

>DmCG32532

RRRHRTTFTQEQLAELEAAFAKSHYPDIYCREELARTTKLNEARIQVWFQNRRAKYRKQE

>DmCG33980

RRHSRTIFTSYQLEKLEEAFKEAHYPDVYAREMLSLKTELPEDRIQVWFQNRRAKWRKTE

>DmCG4136

RRHGRTIFTSSQLEELEKAFKEAHYPDVSARELLSMKTGLAEDRIQVWYQNRRAKWRKTE

>DmCG4328

PKRPRTILNTQQRRAFKASFEVSPKPCRKVRENLAKDTGLSLRIVQVWFQNQRAKVKKIQ

>DmCG7056

RKGGQIRFTSQQTKNLEARFASSKYLSPEERRHLALQLKLTDRQVKTWFQNRRAKWRRAN

>DmCG9876

PRRNRTTFSSAQLTALEKVFERTHYPDAFVREELATKVHLSEARVQVWFQNRRAKFRRNE

>Dmct

SKKQRVLFSEEQKEALRLAFALDPYPNVGTIEFLANELGLATRTITNWFHNHRMRLKQQV

>DmDfd

PKRQRTAYTRHQILELEKEFHYNRYLTRRRRIEIAHTLVLSERQIKIWFQNRRMKWKKDN

>DmDll

MRKPRTIYSSLQLQQLNRRFQRTQYLALPERAELAASLGLTQTQVKIWFQNRRSKYKKMM

>DmDr

NRKPRTPFTTQQLLSLEKKFREKQYLSIAERAEFSSSLRLTETQVKIWFQNRRAKAKRLQ

>DmE5

PKRVRTAFSPTQLLKLEHAFEGNHYVVGAERKQLAQGLSLTETQVKVWFQNRRTKHKRMQ

>Dmems

PKRIRTAFSPSQLLKLEHAFESNQYVVGAERKALAQNLNLSETQVKVWFQNRRTKHKRMQ

>Dmen

EKRPRTAFSSEQLARLKREFNENRYLTERRRQQLSSELGLNEAQIKIWFQNKRAKIKKST

>Dmeve

VRRYRTAFTRDQLGRLEKEFYKENYVSRPRRCELAAQLNLPESTIKVWFQNRRMKDKRQR

>Dmexd

ARRKRRNFSKQASEILNEYFYSNPYPSEEAKEELARKCGITVSQVSNWFGNKRIRYKKNI

>Dmexex

TRRPRTAFTSQQLLELEKQFKQNKYLSRPKRFEVASGLMLSETQVKIWFQNRRMKWKRSK

>Dmey

LQRNRTSFTNDQIDSLEKEFERTHYPDVFARERLAGKIGLPEARIQVWFSNRRAKWRREE

>Dmeyg

FRRNRTTFSPEQLEELEKEFDKSHYPCVSTRERLSSRTSLSEARVQVWFSNRRAKWRRHQ

>Dmftz

SKRTRQTYTRYQTLELEKEFHFNRYITRRRRIDIANALSLSERQIKIWFQNRRMKSKKDR

>Dmgsb

QRRSRTTFSNDQIDALERIFARTQYPDVYTREELAQSTGLTEARVQVWFSNRRARLRKQL

>Dmgsbn

QRRSRTTFTAEQLEALERAFSRTQYPDVYTREELAQTTALTEARIQVWFSNRRARLRKHS

>DmGsc

KRRHRTIFTEEQLEQLEATFDKTHYPDVVLREQLALKVDLKEERVEVWFKNRRAKWRKQK

>DmH2.0

KRKRSWSRAVFSNLQRKGLEIQQKYITKPDRRKLAARLNLTDAQVKVWFQNRRMKWRHTR

>DmHGTX

KKHTRPTFSGQQIFALEKTFEQTKYLAGPERAKLAYALGMSESQVKVWFQNRRTKWRKRH

>DmHmx

KKKTRTVFSRAQVFQLESTFDLKRYLSSSERAGLAASLRLTETQVKIWFQNRRNKWKRQL

>Dmhth

NQKKRGIFPKVATNILRAWLFQHPYPSEDQKKQLAQDTGLTILQVNNWFINARRRIVQPM

>Dmind

SKRIRTAFTSTQLLELEREFSHNAYLSRLRRIEIANRLRLSEKQVKIWFQNRRVKQKKGG

>Dminv

DKRPRTAFSGTQLARLKHEFNENRYLTEKRRQQLSGELGLNEAQIKIWFQNKRAKLKKSS

>DmIP09201

QRRNRTTFTLQQLEELETAFAQTHYPDVFTREDLAMKINLTEARVQVWFQNRRAKWRKAE

>DmIP17602

QRRSRTNFTLDQLNELERLFEETHYPDAFMREELSQRLGLSEARVQVWFQNRRAKCRKHE

>Dmlab

NNSGRTNFTNKQLTELEKEFHFNRYLTRARRIEIANTLQLNETQVKIWFQNRRMKQKKRV

>DmLag1

GIRSSRPKKAANVPILEKTYAKSTRLDKKKLVPLSKQTDMSEREIERWWRLRRAQDKPST

>Dmlbe

KRKSRTAFTNHQIFELEKRFLYQKYLSPADRDEIAASLGLSNAQVITWFQNRRAKQKRDI

>Dmlbl

KRKSRTAFTNQQIFELEKRFLYQKYLSPADRDEIAGGLGLSNAQVITWFQNRRAKLKRDM

>DmLim1

RRGPRTTIKAKQLEVLKTAFNQTPKPTRHIREQLAKETGLPMRVIQVWFQNKRSKERRMK

>DmLim3

NKRPRTTITAKQLETLKTAYNNSPKPARHVREQLSQDTGLDMRVVQVWFQNRRAKEKRLK

>Dmmirr

NGARRKNATRETTSTLKAWLNENPYPTKGEKIMLAIITKMTLTQVSTWFANARRRLKKEN

>Dmnub

RRKKRTSIETTIRGALEKAFLANQKPTSEEITQLADRLSMEKEVVRVWFCNRRQKEKRIN

>Dmoc

QRRERTTFTRAQLDVLEALFGKTRYPDIFMREEVALKINLPESRVQVWFKNRRAKCRQQL

>DmOdsH

KRRGRTNFNSWQLRELERVFQGSHYPDIFMREALATKLDLMEGRIAVWFQNRRAKWRKQE

>Dmonecut

PKKPRLVFTDLQRRTLQAIFKETKRPSKEMQVTIARQLGLEPTTVGNFFMNARRRSMDKW

>DmOptix

GEQKTHCFKERTRSLLREWYLQDPYPNPTKKRELAKATGLNPTQVGNWFKNRRQRDRAAA

>Dmotp

QKRHRTRFTPAQLNELERCFSKTHYPDIFMREEIAMRIGLTESRVQVWFQNRRAKWKKRK

>Dmpb

PRRLRTAYTNTQLLELEKEFHFNKYLCRPRRIEIAASLDLTERQVKVWFQNRRMKHKRQT

>Dmpdm2

RRKKRTSIETTVRTTLEKAFLMNCKPTSEEISQLSERLNMDKEVIRVWFCNRRQKEKRIN

>Dmpdm3

KRKRRTSFTPQALELLNAHFERNTHPSGTEITGLAHQLGYEREVIRIWFCNKRQALKNTV

>DmPHDP

QRRIRTTFTSNQLNELEKIFLETHYPDIYTREEIASKLHLTEARVQVWFQNRRAKFRKQE

>DmPph13

QRRYRTTFNTLQLQELERAFQRTHYPDVFFREELAVRIDLTEARVQVWFQNRRAKWRKQE

>Dmprd

QRRCRTTFSASQLDELERAFERTQYPDIYTREELAQRTNLTEARIQVWFSNRRARLRKQH

>Dmpros

MAPTSSTLTPMHLRKAKLMFFWVRYPSSAVLKMYFPDIKFNTAQLVKWFSNFREFYYIQM

>DmPtx1

QRRQRTHFTSQQLQELEHTFSRNRYPDMSTREEIAMWTNLTEARVRVWFKNRRAKWRKRE

>DmRx

HRRNRTTFTTYQLHELERAFEKSHYPDVYSREELAMKVNLPEVRVQVWFQNRRAKWRRQE

>DmScr

TKRQRTSYTRYQTLELEKEFHFNRYLTRRRRIEIAHALCLTERQIKIWFQNRRMKWKKEH

>Dmscro

RRKRRVLFTQAQVYELERRFKQQRYLSAPEREHLASLIHLTPTQVKIWFQNHRYKCKRQA

>DmSix4

GEETVYCFKEKSRNALKDCYLTNRYPTPDEKKTLAKKTGLTLTQVSNWFKNRRQRDRTPQ

>Dmslou

PRRARTAFTYEQLVSLENKFKTTRYLSVCERLNLALSLSLTETQVKIWFQNRRTKWKKQN

>Dmso

GEETSYCFKEKSRSVLRDWYSHNPYPSPREKRDLAEATGLTTTQVSNWFKNRRQRDRAAE

>Dmtoe

FRRNRTTFSPEQLDELEKEFDKSHYPCVNTREKLAARTALSEARVQVWFSNRRAKWRRHQ

>Dmtoy

LQRNRTSFSNEQIDSLEKEFERTHYPDVFARERLADKIGLPEARIQVWFSNRRAKWRREE

>Dmtup

PTRVRTVLNEKQLHTLRTCYNANPRPDALMKEQLVEMTSLSPRVIRVWFQNKRCKDKKKT

>DmUbx

RRRGRQTYTRYQTLELEKEFHTNHYLTRRRRIEMAHALCLTERQIKIWFQNRRMKLKKEI

>Dmunc4

RRRSRTNFNSWQLEELERAFSASHYPDIFMREALAMRLDLKESRVAVWFQNRRAKVRKRE

>Dmunpg

SRRRRTAFTSEQLLELEREFHAKKYLSLTERSQIATSLKLSEVQVKIWFQNRRAKWKRVK

>Dmvis

LRKRRGNLPKSSVKILKRWLYENAYPSDAEKFTLSQEANLTVLQVCNWFINARRRILPEM

>Dmvnd

KRKRRVLFTKAQTYELERRFRQQRYLSAPEREHLASLIRLTPTQVKIWFQNHRYKTKRAQ

>Dmvvl

KRKKRTSIEVSVKGALEQHFHKQPKPSAQEITSLADSLQLEKEVVRVWFCNRRQKEKRMT

>Dmzfh1

KVRVRTAINEEQQQQLKQHYSLNARPSRDEFRMIAARLQLDPRVVQVWFQNNRSRERKMQ

>Dmzfh2I

QKRARTRITDDQLKILRAHFDINNSPSEESIMEMSQKANLPMKVVKHWFRNTLFKERQRN

>Dmzfh2II

KRANRTRFTDYQIKVLQEFFENNSYPKDSDLEYLSKLLLLSPRVIVVWFQNARQKQRKIY

>Dmzfh2III

NKRLRTTILPEQLNFLYECYQSESNPSRKMLEEISKKVNLKKRVVQVWFQNSRAKDKKSR

>PsXlox

NKRTRTAYTRAQLLELEKEFHFNKYISRPRRIELAAMLNLTERHIKIWFQNRRMKWKKDE

>AmPrep

GRQKRGVLPKQATSIMRTWLFEHPYPTEDEKRQIASQTNLTLLQVNNWFINARRRILQPM

>NvPOU1

RRKRRTTIGLAAKEALENHFMKQTKPSSPEIVRIADGLRLDKEVVRVWFCNRRQREKRVK

>NvHNF

SRRNRFKWGPASTNILYQSYEQQRNPSKEEREALVEACNVTESRVYNWFANRRKEETFRM

>NvCART1

KRRNRTTFTAYQLEEMERVFQKTHYPDVYTREQLALRCALTEARVQVWFQNRRAKWRKRE

>Hvmanacle

HRRVRTAFTHHQLTTLERTFETSHYPDVVLRERLASFTGLAESRIQVWFKNRRAKYRKHQ

>SmHox3b

NKRSRTAYTQSQLVELEKEFHFNRYLCRPRRVELASMLNLTERQIKIWFQNRRMKNKKIK

>PdNK4

KRKPRVLFSQAQVYELERRFKQQRYLSAPEREQLASMLKLTSTQVKIWFQNRRYKMKRQR
