# Supplementary material for: Classification and nomenclature of all human homeobox genes
Source: BMC Biol. 2007 Oct 26;5:47. doi: 10.1186/1741-7007-5-47 (PMC2211742; doi:10.1186/1741-7007-5-47)
Supplement: Additional file 5 — Neighbor-joining phylogenetic tree of human homeodomains excluding ANTP and PRD classes, for comparison to maximum likelihood tree. Arbitrarily rooted phylogenetic tree of human homeodomains excluding the ANTP and PRD classes constructed using the neighbor-joining method. Bootstrap values supporting internal nodes with over 70% are shown. Homeodomain sequences derived from pseudogenes are excluded. Roman numeral suffixes are used to distinguish multiple homeodomains encoded by a single gene. Classes and/or families are color coded as shown in the key. The LIM and ZF classes are not recovered as two distinct monophyletic groups, a result also found by maximum likelihood analysis (Figure 3). The multiple homeodomains of Zfhx-family proteins and Zhx/Homez-family proteins are also dispersed in the tree, presumably artefactually. Monophyly of the CUT class is not recovered in this tree, but is by maximum likelihood analysis (Figure 3). Detailed relationships between different gene families should not be inferred from this tree. [file 1741-7007-5-47-S5.pdf]

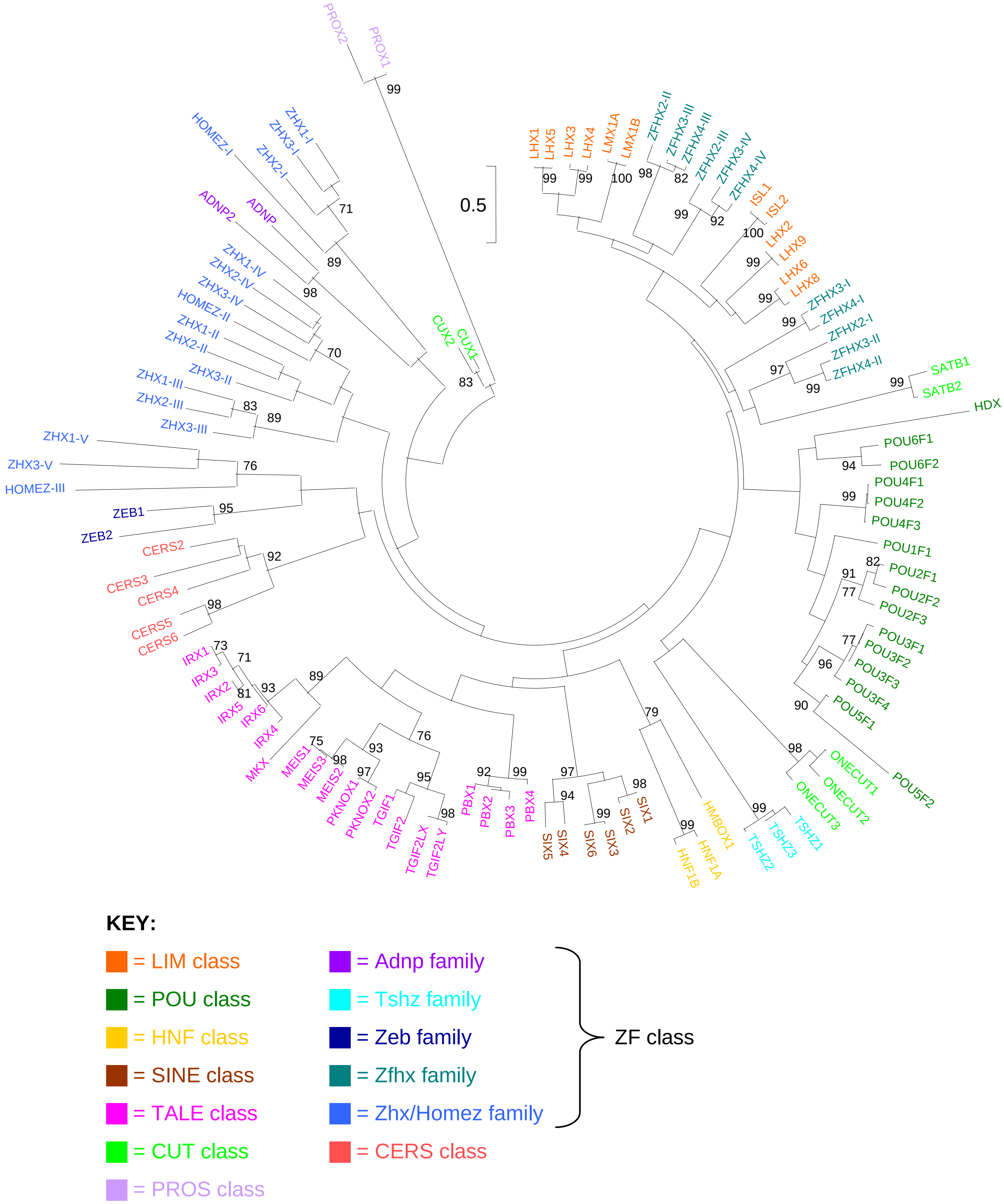
0.5
PROX2
PROX1
LHX1
LHX5
LHX3
LHX4
LMX1A
LMX1B
ZFHX2-II
ZFHX3-III
ZFHX4-III
ZFHX2-III
ZFHX3-IV
ZFHX4-IV
ISL1
ISL2
LHX2
LHX9
LHX6
LHX8
ZFHX3-I
ZFHX4-I
ZFHX2-I
ZFHX3-II
ZFHX4-II
SATB1
SATB2
HDX
POU6F1
POU6F2
POU4F1
POU4F2
POU4F3
POU1F1
POU2F1
POU2F2
POU2F3
POU3F1
POU3F2
POU3F3
POU3F4
POU5F1
POU5F2
ONECUT1
ONECUT2
ONECUT3
TSHZ1
TSHZ3
TSHZ2
HMBOX1
HNF1A
HNF1B
SIX1
SIX2
SIX3
SIX6
SIX4
SIX5
PBX4
PBX3
PBX2
PBX1
TGIF2LY
TGIF2LX
TGIF2
TGIF1
PKNOX2
PKNOX1
MEIS2
MEIS3
MEIS1
MKX
IRX4
IRX6
IRX5
IRX2
IRX3
IRX1
CERS6
CERS5
CERS4
CERS3
CERS2
ZEB2
ZEB1
HOMEZ-III
ZHX3-V
ZHX1-V
ZHX3-III
ZHX2-III
ZHX1-III
ZHX3-II
ZHX2-II
ZHX1-II
HOMEZ-II
ZHX3-IV
ZHX2-IV
ZHX1-IV
ADNP2
ADNP
HOMEZ-I
ZHX2-I
ZHX3-I
ZHX1-I
CUX2
CUX1
KEY:
= LIM class
= POU class
= HNF class
= SINE class
= TALE class
= CUT class
= PROS class
= Adnp family
= Tshz family
= Zeb family
= Zfhx family
= Zhx/Homez family
= CERS class
ZF class
